# Effects of the ligand linkers on stability of mixed-valence Cu(I)Cu(II) and catalytic aerobic alcohol oxidation activity

**DOI:** 10.1038/s41598-024-66227-2

**Published:** 2024-07-05

**Authors:** Attawit Jehdaramarn, Teera Chantarojsiri, Thanapat Worakul, Panida Surawatanawong, Kittipong Chainok, Preeyanuch Sangtrirutnugul

**Affiliations:** 1 Center of Excellence for Innovation in Chemistry (PERCH-CIC), Department of Chemistry, Faculty of Science, Mahidol University, Bangkok, Thailand; 2 Center of Sustainable Energy and Green Materials, Mahidol University, Salaya, 73170 Nakhon Pathom Thailand; 3 Thammasat University Research Unit in Multifunctional Crystalline Materials and Applications (TU-MCMA), Faculty of Science and Technology, Thammasat University, Pathum Thani, 12121 Thailand

**Keywords:** Chemistry, Materials science

## Abstract

We synthesized a class of ligands that feature single (**L1**) and dual amine-bis(triazole) chelates (**L2** with a 1,3-phenylene linker and **L3** with a 1,5-naphthalene linker). Our findings which were derived from UV–Vis titrations, crystal structure analysis of relevant copper complexes, and DFT calculations indicate the formation of both mononuclear CuBr(**L1**) and dinuclear (μ-**Ln**)(CuBr)_2_ (**Ln** = **L2** and **L3**) complexes. The catalytic activities of CuBr/**Ln**, in combination with TEMPO (2,2,6,6-tetramethylpiperidin-1-yl)oxyl) co-catalyst and NMI (*N*-methylimidazole) for aerobic alcohol oxidation, reveal the following activity trend: CuBr/**L3** > CuBr/**L2** > CuBr/**L1**. Furthermore, electrochemical data from in-situ generated CuBr complexes suggest that the higher catalytic performance of CuBr/**L3** is attributed to the presence of less stable mixed-valence and more reducible Cu(I)-**L3**-Cu(II) species compared to Cu(I)-**L2**-Cu(II). This difference is a result of weaker σ interactions between Cu–N_amine_, larger bridging π systems, and a longer Cu···Cu distance in the presence of **L3**. Additionally, the catalyst system, CuBr/**L3**/TEMPO/NMI, efficiently promotes the aerobic oxidation of benzyl alcohol to benzaldehyde at room temperature in CH_3_CN with a high turnover frequency (TOF) of 38 h^−1^ at 1 h.

## Introduction

Copper-catalyzed aerobic alcohol oxidation is an efficient and sustainable method for converting primary alcohols to aldehydes. This process utilizes environmentally friendly molecular oxygen as the oxidant, eliminating the need for traditional stoichiometric oxidants such as NaOCl, MnO_2_, Cr(IV), Swern, or the Dess–Martin reagent^1–4^. Over the years, various copper catalysts have been explored for aerobic alcohol oxidation, particularly mononuclear copper complexes supported by N-based ligands such as 2,2′-bipyridine^5^, triazole^6–8^, and terpyridine^9^. These copper catalysts are usually combined with a nitroxyl radical such as 2,2,6,6-tetramethylpiperidine-1-oxyl radical (TEMPO), which serves as the co-catalyst, and a base, e.g., N-methylimidazole (NMI), DMAP, and K_2_CO_3_, to promote the aerobic alcohol oxidation^10,11^.

**Figure 1:**
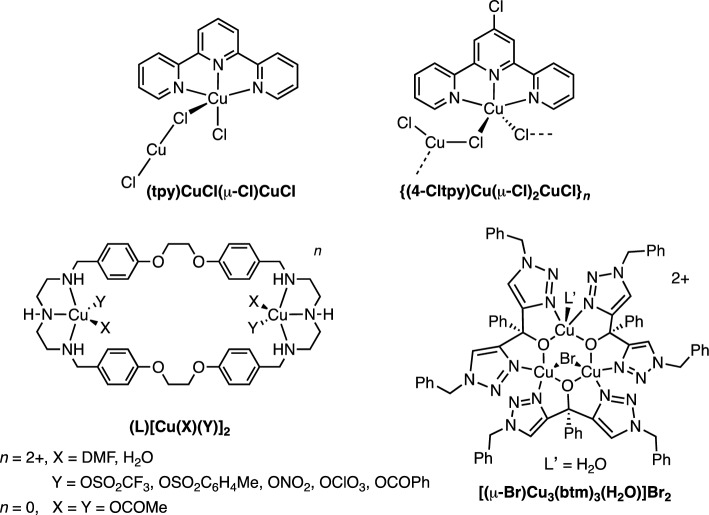
Examples of multinuclear copper catalysts for aerobic alcohol oxidation.

Recently, multinuclear copper catalysts, inspired by metalloprotein oxidase enzymes possessing multiple metal active sites such as catechol oxidase, laccases, and superoxide dismutase, have gained attention due to their ability to facilitate multi-electron transfer^12,13^. In 2015, Zhang and colleagues isolated the mixed-valence Cu(I)Cu(II) complexes (tpy)CuCl(μ-Cl)CuCl and (4-Cltpy)Cu(μ-Cl)_2_CuCl, as depicted in Fig. 1, by reacting CuCl_2_ (1.0 equiv) with terpyridine derivatives (tpy or 4-Cltpy; 1.0 equiv) and TEMPO (0.5 equiv) in a CH_2_Cl_2_/MeOH solution. These mixed-valence complexes exhibited superior catalytic oxidation activities compared to related mononuclear Cu complexes. Specifically, 1 mol% of (tpy)CuCl(μ-Cl)CuCl or (4-Cltpy)Cu(μ-Cl)_2_CuCl with 5 mol% TEMPO/DMAP afforded quantitative yields of benzaldehyde at room temperature after 5 h. Meanwhile, the related mononuclear complexes (tpy)CuCl and (4-Cltpy)Cu(μ-Cl)_2_ (1 mol%) with 5 mol% TEMPO required 15 mol% DMAP and the reaction temperature of 70 °C to achieve high yields of the oxidized product (90–95%)^9^. These results suggest that both Cu(I)Cu(II) cores and higher copper nuclearities may enhance catalytic oxidation activities. Additionally, Ma et al. reported a series of catalyst systems consisting of dinuclear Cu(II) complexes based on a N_6_O_4_ macrocyclic ligand (L) and TEMPO for aerobic alcohol oxidation in an alkaline solution (Fig. 1)^14^. The high catalytic activities of (L)[Cu(X)(Y)]_2_ with the TON value reaching up to 232 (for benzaldehyde) after 20 h at 70 °C were attributed to hydrophobic internal environments surrounding the copper centers, consequently promoting the binding of O_2_ and alcohol substrate molecules to the copper active sites. Furthermore, our group has recently shown that catalytic activities of trinuclear copper(II) catalysts supported by bis(triazolyl) ligands [(μ-Br)Cu_3_(btm)_3_(H_2_O)]Br_2_ (5 mol% Cu), in the presence of TEMPO (5 mol%) and NMI (10 mol%) in CH_3_CN, toward aerobic oxidation of benzyl alcohol were better than that of the related mononuclear Cu(II) complex containing triazolylphenylmethanol ligands^8^. However, the catalytic performances of these catalysts were moderate with the highest TOF value of 9.8 h^−1^ for benzaldehyde at room temperature after 3 h. It is possible that structural rigidity around Cu centers of the trinuclear Cu cluster impedes a geometrical rearrangement from pseudo-square pyramidal Cu(II) to tetrahedral Cu(I), a key mechanistic step in the alcohol oxidation catalysis, which leads to slower reactions.

**Figure 2:**
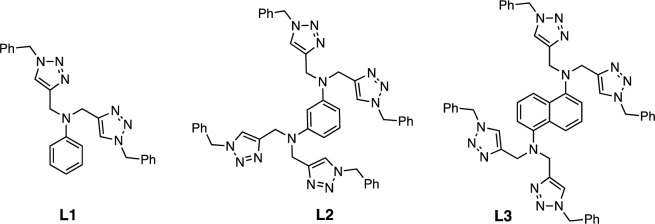
Structures of the amine-bis(triazolyl) ligands **L1**–**L3.**

More recently, we have reported exceptional activities of the copper catalyst systems featuring amine-triazole ligands functionalized with poly(ethylene glycol) substituents for aerobic oxidation of activated 1° alcohols to the corresponding aldehydes in water^15^. Given these promising results and the potential oxidation activities exhibited by multinuclear copper catalysts, we developed a class of amine-bis(triazolyl) ligands **L1**–**L3** which possess either a single (**L1**) and dual copper chelating sites (**L2** and **L3**), as shown in Fig. 2. With **L2** and **L3** ligands, structures of the multinuclear copper complexes are not rigid and copper centers are expected to electronically interact through the inductive effect and MLCT (i.e., *d* electrons (Cu) → π* (LUMO) of the ligands)^16^, potentially resulting in improved catalytic oxidation activities. Herein, we aim to assess the effects of aromatic amine-triazole linkers on aerobic alcohol oxidation activities of multinuclear copper catalysts. In particular, the role of aromatic linkers, specifically 1,3-phenylene and 1,5-naphthalene, in shaping the structures of copper complexes and influencing the degree of Cu···Cu interactions were investigated using various techniques including single crystal X-ray crystallography, computational studies, and cyclic voltammetry analysis.

## Experimental section

### Materials

All air-sensitive reactions were carried out in dry glassware with dry solvents under N_2_ atmosphere in a glovebox or using standard Schlenk techniques. All reagents including propargyl bromide (> 97% stabilized in MgO) were purchased from TCI and Sigma-Aldrich and used without further purifications. Reagent-grade solvents were purchased from LabScan. Benzyl azide^8^ and *N*,*N*-dipropargylaniline^17^ were prepared following literature methods.

NMR spectra were acquired in deuterated solvents at room temperature using a Bruker-Ascend™ 400 high-resolution magnetic resonance spectrometer for ^1^H (400 MHz) and ^13^C{^1^H} (100 MHz) nuclei with chemical shifts referenced to residual solvent peaks. Fourier transform-infrared (FT-IR) spectra were collected on a Bruker model Alpha spectrometer from solid samples. Electrospray Ionization mass spectra (ESI–MS) of CH_3_CN solutions of **TP2**, **TP3**, **L2**, and **L3** were obtained in positive-ion mode on a Bruker micrOTOF II. Elemental analyses were performed using Perkin Elmer 2400 CHN. A Shimadzu UV-2600 UV–Vis spectrophotometer was used to record the absorption data of aqueous solutions of CuBr/**L1–L3** in the range of 200–800 nm. Computational details of CuBr/**L1–L3** were described in the Electronic Supporting Information. The Bruker D8 QUEST CMOS PHOTON II diffractometer (λ_Mo_ = 0.71073 Å) was employed to obtain X-ray diffraction data for **1** and **4** at a temperature of 296 K, while the Bruker D8 Venture CMOS PHOTON I diffractometer (λ_Cu_ = 1.54178 Å) was utilized at temperatures of 100 K and 296 K for complexes **2** and **3**, respectively. Product conversions obtained from catalytic experiments were analyzed by GLC on a 6890N Agilent Technologies gas chromatograph equipped with a 5973N Agilent Technologies quadrupole mass detector with anisole as an internal standard.

## Synthesis and characterization

### Synthesis of tetrapropargyl diamine compounds TP2 and TP3.

The synthesis of **TP2** and **TP3** follows the literature method for *N*,*N*-dipropargylaniline (**DP1**)^17^ with slight modifications. To a mixture of the amine substrate and K_2_CO_3_ was added 10 ml of DMF. After stirring until the homogeneous solution was obtained, propargyl bromide was added and the reaction mixture was stirred at room temperature for 2 days. Then, the solution was extracted with 3 × 50 mL CH_2_Cl_2_ and the combined filtrate was washed with 3 × 50 mL DI water, followed by 50 mL of saturated brine solution. The CH_2_Cl_2_ solution was dried with anhydrous Na_2_SO_4_ and all volatiles were removed under vacuum to afford the spectroscopically clean product.

#### *N*,*N*,*N'*,*N'*-tetrapropargyl-1,3-benzenediamine (TP2)

The substrates and reagents are 1,3-diaminobenzene (1.00 g, 9.25 mmol), K_2_CO_3_ (5.2 g, 38 mmol), and propargyl bromide (5.0 mL, 55.5 mmol). The product was obtained as dark brown oil in 82% yield (1.98 g, 7.61 mmol). ^1^H NMR (400 MHz, CDCl_3_): δ 7.19 (t *J* = 8 Hz, 1H, Ar*H*), 6.61 (d *J* = 4 Hz 1H, Ar*H*), 6.54 (t *J* = 4 Hz, 2H, Ar*H*), 4.10 (s, 8H, NC*H*_2_), 2.26 (s, 4H, ≡C*H*). ^13^C{^1^H} NMR (100 MHz, CDCl_3_): δ 146.8, 127.7, 106.4, 102.2, (aromatic *C*s), 79.4 (*C*≡CH), 72.9 (C≡*C*H) 40.8 (*C*H_2_). FT-IR (cm^−1^): 3289 (s, C≡*C-H*), 2118 (w, *C≡C*). HRMS (*m*/*z*): Calcd. for C_18_H_17_N_2_ [M + H]^+^ 261.1391, Found 261.1388.

#### *N*,*N*,*N'*,*N'*-tetrapropargyl-1,5-naphthalenediamine (TP3)

The substrates and reagents are 1,5-diaminonaphthalene (2.00 g, 12.6 mmol), K_2_CO_3_ (8.0 g, 75.6 mmol), and propargyl bromide (7.0 mL, 75.6 mmol). The product was obtained as a dark brown solid in 61% yield (2.39 g, 7.70 mmol). ^1^H NMR (400 MHz, CDCl_3_): δ 8.04 (d *J* = 8 Hz, 2H, Ar*H*), 7.45 (t *J* = 8 Hz, 2H, Ar*H*), 7.34 (d *J* = 4 Hz 2H, Ar*H*), 4.10 (s, 8H, NC*H*_2_), 2.31 (s, 4H, ≡C*H*). ^13^C{^1^H} NMR (100 MHz, CDCl_3_): δ 144.5, 128.5, 123.2, 118.0, 115.8 (aromatic *C*s), 79.4, (*C*≡CH) 73.5 (C≡*C*H), 42.7 (*C*H_2_). HRMS (*m*/*z*): Calcd. for C_22_H_19_N_2_ [M + H]^+^ 311.1548, Found 311.1540. FT-IR (cm^−1^): 3267 (s, C≡*C-H*), 2112 (w, *C≡C*). Anal. Calcd. for C_22_H_18_N_2_·1.6H_2_O: C, 77.89; H, 6.30; N, 8.26 Found: C, 77.68; H, 5.92; N, 8.30.

### Synthesis of bis- and tetra(triazolyl) ligands L1–L3

The syntheses of **L1**–**L3** follow that of **L1**^18^ with some modifications. Under N_2_, to a mixture of dipropargylamine (for **L1**) or tetrapropargylamine (for **L2** and **L3**) and PhCH_2_N_3_ was added 10 mL of CH_2_Cl_2_ followed by NEt_3_. The reaction solution was stirred at room temperature until homogeneous. Then, the reaction flask was wrapped with an aluminum foil before CuI was added. After 24 h, the reaction mixture was stirred with EDTA in 10% aqueous NH_4_OH at room temperature for 8 h, after which the solution was extracted with 3 × 50 mL CH_2_Cl_2_ and the organic filtrate was washed with 50 mL of saturated brine solution. The combined organic layers were dried with anhydrous Na_2_SO_4_, filtered, and the solvent was evaporated under vacuum and crystallized in a 1:1 CH_2_Cl_2_/diethyl ether solution to afford the product.

#### *N*,*N*-bis((1-benzyl-1*H*-1,2,3-triazol-4-yl)methyl)aniline (L1)^18^

Compound **DP1** (0.62 g, 3.66 mmol), PhCH_2_N_3_ (1.08 g, 7.32 mmol), NEt_3_ (1.2 mL, 8.7 mmol), CuI (0.35 g, 1.9 mmol), EDTA (0.6 g, 1.9 mmol). **L1** was obtained as a light orange solid in 74% yield (1.1 g, 2.66 mmol). ^1^H NMR (400 MHz, DMSO-*d*_6_): 8.00 (s, 2H, trz*H*), 7.37–7.28 (m, 6H, Ar*H*), 7.25–7.22 (m, 4H, Ar*H*), 7.14–7.08 (m, 2H, Ar*H*), 6.87–6.84 (m, 2H, Ar*H*), 6.60 (m, 1H, Ar*H*), 5.53 (s, 4H, C*H*_2_Ph), 4.61(s, 4H, NC*H*_2_).

#### *N*,*N*,*N'*,*N'*-tetra((1-benzyl-1*H*-1,2,3-triazol-4-yl)methyl)phenylene-1,3-diamine (L2)

Compound **TP2** (0.60 g, 2.3 mmol), PhCH_2_N_3_ (1.4 g, 10.1 mmol), NEt_3_ (1.0 mL, 10.1 mmol), CuI (0.44 g, 2.3 mmol), EDTA (0.67 g, 2.3 mmol). **L2** was obtained as light brown powder in 81% yield (1.3 g, 1.4 mmol). ^1^H NMR (400 MHz, DMSO-*d*_6_): δ 8.00 (s, 4 H, trz*H*), 7.33–7.26 (m, 20H, Ph*H*), 6.84 (t *J* = 8 Hz, 1H, Ar*H*), 6.43 (m, 1H, Ar*H*), 6.16 (m, 2H, Ar*H*), 5.53 (s, 8H, C*H*_2_Ph), 4.51 (s, 8H, NC*H*_2_). ^13^C{^1^H} NMR (100 MHz, DMSO-*d*_*6*_): δ 148.7, 145.0, 136.1, 129.3, 128.7, 128.0, 127.8, 123.2, 102.4, 97.9 (aromatic *C*s), 52.7, 45.5 (N*C*H_2_). HRMS: Calcd. for C_46_H_45_N_14_ [M + H]^+^ 793.3952, Found 793.3949. Anal. Calcd. for C_46_H_44_N_14_·0.2C_6_H_6_·0.5CHCl_3_·0.3H_2_O: C, 65.57; H, 5.34; N, 22.45 Found: C, 65.32; H, 4.89; N, 22.16.

#### *N*,*N*,*N'*,*N'*-tetra((1-benzyl-1*H*-1,2,3-triazol-4-yl)methyl)napthalene-1,5-diamine (L3)

Compound **TP3** (0.61 g, 1.9 mmol), PhCH_2_N_3_ (1.1 g, 8.5 mmol), NEt_3_ (1.4 mL, 10.0 mmol), CuI (0.37 g, 1.9 mmol), EDTA (0.61 g, 1.9 mmol). **L3** was obtained as a light brown solid in 41% yield (0.67 g, 0.80 mmol). ^1^H NMR (400 MHz, DMSO-*d*_6_): δ 8.10 (d *J* = 8 Hz, 2H, Ar*H*), 7.86 (s, 4H, trz*H*), 7.32–7.24 (m, 14H, Ar*H*), 7.14–7.10 (m, 8H, Ar*H*), 7.00 (d *J* = 4 Hz, 2H, Ar*H*), 5.50 (s, 8H, NC*H*_2_Ph), 4.32 (s, 8H, NC*H*_2_trz). ^13^C{^1^H} NMR (100 MHz, DMSO-*d*_*6*_): δ 146.6, 144.0, 143.8, 136.2, 130.8, 128.7, 127.9, 127.5, 124.9, 123.9, 118.3 (aromatic *C*s), 52.6 48.3 (N*C*H_2_). HRMS: Calcd. for C_50_H_47_N_14_ [M + H]^+^ 843.4108, Found 843.4105. Anal. Calcd. for C_50_H_46_N_14_·CH_2_Cl_2_·2CH_3_CN·0.33C_6_H_6_: C, 66.07; H, 5.45; N, 21.64 Found: C, 66.38; H, 5.52; N, 21.98.

### Synthesis of copper(II) nitrate complexes

To a 10 mL CH_2_Cl_2_ solution of the triazolyl ligand was added a 10 mL EtOH solution of Cu(NO_3_)_2_·3H_2_O. The reaction mixture was stirred at room temperature for 24 h, after which all volatiles were removed in vacuo. The product was isolated via crystallization.

#### [Cu(L1)_2_][(NO_3_)]_2_ (1)

**L1** (0.20 g, 0.46 mmol) and Cu(NO_3_)_2_·3H_2_O (0.11 g, 0.47 mmol) were used. Slow evaporation from the 1:1 CH_3_OH:Et_2_O solution of the complex afforded green crystals in 50% yield (0.13 g, 0.12 mmol). X-ray quality crystals of **1** was obtained from a layer diffusion of diethyl ether onto the methanol solution of **1**. Anal. Calcd. for C_52_H_50_CuN_16_O_6_·2H_2_O·HNO_3_: C, 53.95; H, 4.79; N, 20.57 Found: C, 53.75; H, 4.41; N, 20.53.

#### {[Cu(L2)][Cu(NO_3_)_4_]}_*n*_ (2)

**L2** (0.19 g, 0.24 mmol) and Cu(NO_3_)_2_·3H_2_O (0.060 g, 0.25 mmol) was used. A layer diffusion of CH_3_OH onto a CH_3_CN solution afforded an X-ray quality, light brown microcrystalline solid in 78% yield (0.12 g, 0.10 mmol). Anal. Calcd. for C_46_H_44_Cu_2_N_18_O_12_·1.5CH_3_OH·0.66C_6_H_6_: C, 48.77; H, 4.29; N, 19.89 Found: C, 48.97; H, 3.99; N, 20.07.

#### {[Cu(L3)(NO_3_)](NO_3_)}_*n*_ (3)

**L3** (0.20 g, 0.24 mmol) and Cu(NO_3_)_2_·3H_2_O (0.060 g, 0.25 mmol) were used. A layer diffusion of CH_3_OH onto a CH_3_CN solution afforded an X-ray quality, dark brown microcrystalline solid in 53% yield (0.16 g, 0.13 mmol). Anal. Calcd. for C_50_H_46_CuN_16_O_6_·CH_2_Cl_2_·2.5HNO_3_·0.5H_2_O: C, 47.78; H, 4.05; N, 20.22 Found: C, 47.39; H, 3.90; N, 20.16.

#### Synthesis of (CuBr_2_)_2_(L3) complex

A mixture of CuBr (0.037 g, 0.26 mmol) and **L3** (0.20 g, 0.24 mmol) was stirred in 7 mL CH_3_CN at room temperature for 12 h, after which the reaction mixture was filtered. Slow evaporation from the CH_3_CN solution afforded X-ray quality, brown crystals in 14% yield (0.026 g, 0.018 mmol) after 4 d. Anal. Calcd. for C_50_H_46_Cu_2_N_14_Br_4_·H_2_O: C, 41.01; H, 3.30; N, 13.40 Found: C, 40.84; H, 3.14; N, 13.73.

### General procedure for alcohol oxidation

The stock solution of the CuBr/**Ln** (**Ln** = **L1**–**L3**) catalyst was prepared from stirring a mixture of CuBr (7.2 mg, 0.050 mmol) and 1.0 equiv **L1** (0.050 mmol) or 2.0 equiv **L2**, **L3** (0.10 mmol) in 5 mL of CH_3_CN at room temperature for 15 min, after which the CH_3_CN solution was filtered. The volume of the resulting filtrate was adjusted to 10.0 mL with CH_3_CN.

For a typical catalytic reaction, 1.0 mmol of alcohol was dissolved in the 5.0 mL CH_3_CN stock solution of CuBr/**Ln** (**Ln** = **L1**–**L3**) followed by an addition of TEMPO (7.9 mg, 0.050 mmol), NMI (8.0 μL, 0.10 mmol), and anisole (10.9 μL, 0.010 mmol). The reaction solution was allowed to stir at room temperature for a given time, after which it was filtered through a short silica column using EtOAc as an eluent. The percentage conversions were determined using GC–MS methods with anisole as an internal standard.

For the reusability study, benzyl alcohol (1.0 mmol) was dissolved in a 10.0 mL CH_3_CN stock solution of CuBr/**L3**, followed by an addition of TEMPO (7.9 mg, 0.050 mmol) and NMI (8.0 µL, 0.10 mmol). After 2 h, 1.00 mL of the reaction mixture was collected and filtered through a short silica column. A 100 µL aliquot of the filtrate was sampled and diluted to 1.5 mL with EtOAc, to which 0.010 mmol of anisole was added as an internal standard. The percent conversion of the resulting solution was determined via GC–MS. To initiate the subsequent catalytic run, benzyl alcohol (1.0 mmol), TEMPO (0.050 mmol), and NMI (0.10 mmol) were added to the remaining reaction mixture. After another 2 h, the reaction mixture was sampled, filtered, and diluted following the same workup procedure described above. The percent conversions for the five subsequent runs were also determined using GC–MS with anisole as an internal standard.

### Cyclic voltammetry

Voltammograms were recorded at ambient temperatures with CH Instruments CHI 620E with CH Instrument software. The CuBr/**Ln** complexes (**Ln** = **L1**–**L3**) (5.0 mM) was dissolved in 0.1 M of [Bu_4_N][PF_6_] in CH_3_CN as the supporting electrolyte. Electrochemical measurements were performed in air at a scanning rate of 0.1 V s^−1^ with a glassy carbon working electrode, a platinum wire counter electrode, and a Ag/Ag^+^ reference electrode. Ferrocene/ferrocenium redox couple was used as an internal standard.

## Results and discussion

### Synthesis of ligands and copper complexes

**Figure 3:**
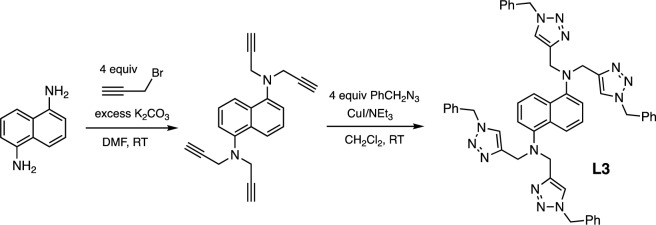
Synthetic pathway of **L3.**

**Figure 4:**
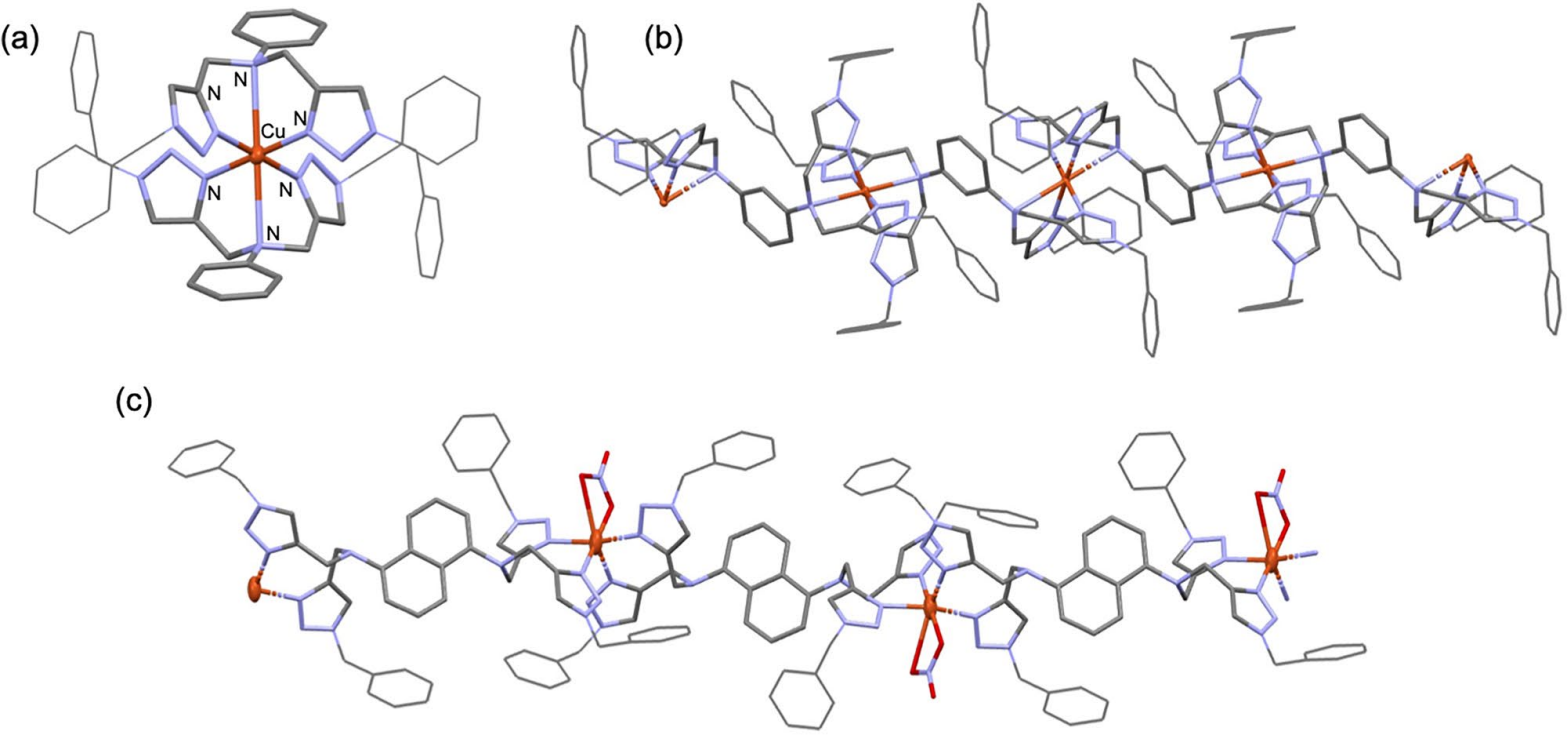
Crystal structures of **1**–**3**. All H atoms are omitted for clarity. Color code: Cu, orange; N, light purple; C, gray; O, red. (a) complex **1** [Cu (**L1**)_2_][NO_3_]_2_. Outer-sphere NO_3_^−^ ions and a CH_3_OH molecule are not shown. (b) complex **2** {[Cu(**L2**)_2_][Cu(NO_3_)_4_]}_*n*_. [Cu(NO_3_)_4_]^2−^ and CH_3_CN molecules are not shown. (c) complex **3** {[Cu(**L3**)(NO_3_)][NO_3_]}_*n*_. Outer-sphere NO_3_^−^ ions are not shown.

The *N*,*N*-bis(triazolylmethyl)aniline ligand **L1** and the *N*,*N*,*N'*,*N'*-tetra(triazolylmethyl)amine ligands **L2** and **L3** (Fig. 2) were synthesized using a multi-step process starting with the propargylation of the corresponding aryl amine substrates. Subsequently, the Cu-catalyzed azide-alkyne cycloaddition (CuAAC) between the resulting alkynes and benzyl azide, promoted by CuI/NEt_3_ in CH_2_Cl_2_ afforded **L1** and **L2** in 74% and 81% yields, respectively. However, under the same conditions, the yield of the naphthalene-containing ligand **L3** was notably lower at 41% (Fig. 3). The reaction of **L1**, which possesses a single metal binding site, with Cu(NO_3_)_2_·3H_2_O in a mixture of CH_3_OH/CH_2_Cl_2_ solvent led to the formation of the copper(II) complex **1**. Single crystals obtained from a layer diffusion of CH_3_OH onto the diethyl ether solution of **1** crystallize in the *P*$\overline{1}$ space group, with the Cu atom occupying the centrosymmetric position. X-ray crystallographic analysis reveals that each asymmetric unit of **1** contains a mononuclear bis-chelate Cu(II) complex, displaying tetragonally distorted octahedral geometry at Cu (Fig. 4a). The complex is accompanied by two outer-sphere NO_3_^−^ counterions and a CH_3_OH molecule. The **L1** ligand coordinates to Cu(II) in a tridentate, facial coordination mode. Notably, the Cu–N_amine_ bond (2.722 Å) is significantly longer compared to the Cu–N_trz_ bonds (1.948(2) Å and 2.022(2) Å), as a result of Jahn–Teller distortion.

On the other hand, crystal structures of **2** and **3** reveal one-dimensional polymeric structures, where **L2** and **L3** ligands act as organic linkers between Cu(II) ions (Fig. 4b,c). The crystal structure of **2** contains bis-chelate Cu(II) ions, which are linked by 1,3-phenylene moieties, and Cu(NO_3_)_4_^2−^ as the counterions. In the {Cu(**L2**)_2_}_*n*_ complexes, each Cu atom is coordinated with two tridentate **L2** ligands in a facial coordination mode. Comparable to **1**, the Cu–N_amine_ bond of **2** is approximately 0.69 Å longer than the Cu–N_trz_ bonds (2.687 cf. 1.970(2) and 2.002(2) Å). However, for **3**, due to the more electron delocalized nature of naphthalene, the N_amine_ atom becomes a weaker Lewis base. Consequently, **L3** binds to Cu(II) ions in a bidentate fashion with only Cu–N_trz_ coordination present. The solid-state structure of **3** shows distorted octahedral bis-chelate Cu(II) complexes containing two bidentate N_trz_ chelates occupying *cis* positions (Fig. 4c). In the mean time, *k*^2^-*O*,*O*-NO_3_^–^ serves as an inner-sphere ligand occupying the two remaining coordination sites, while the other NO_3_^–^ ion acts as a counterion to balance the charges. It should be noted that the Cu···Cu distances separated by aromatic 1,3-phenylene and 1,5-naphthalene linkers are 8.172 Å and 13.970 Å, respectively.

**Figure 5:**
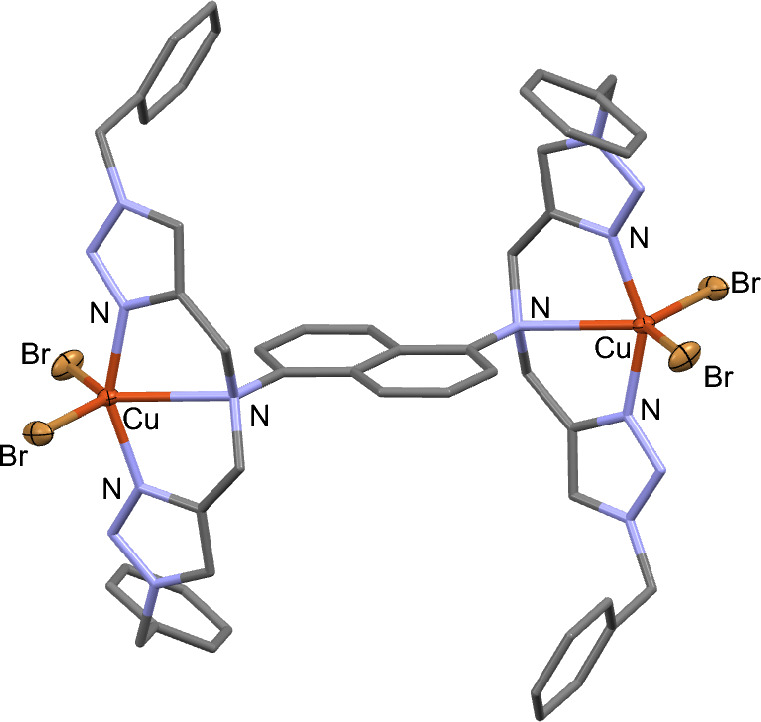
Crystal structure of (μ-**L3**)(Cu^II^Br_2_)_2_ (**4**). All H atoms were omitted for clarity while Cu and Br atoms were shown as thermal ellipsoids drawn at the 30% probability level.

We investigated the structure of CuBr/**Ln** by mixing CuBr with an equimolar amount of **L3** in CH_3_CN at room temperature overnight followed by slow evaporation, which resulted in single crystals of **4** in 14% yield after 4 d. However, crystallographic analysis revealed the dinuclear Cu(II) complexes, as shown in Fig. 5. It is plausible that, under aerobic conditions, Cu(I)/**L3** undergoes disproportionation,^19^ leading to the formation of Cu(II)/**L3** and Cu^0^. Complex **4** is centrosymmetric, where both Cu(II) centers are related by inversion symmetry. The five-coordinate Cu center assumes a geometry intermediate between a trigonal bipyramid and a square-base pyramid (τ = 0.4), featuring an elongated Cu–N_amine_ bond compared to the average Cu–N_trz_ bond length (2.610(3) *vs*. 1.958(3) Å). With the tridentate coordination of **L3**, the Cu···Cu distance of 10.827 Å is closer than that observed in complex **3** (13.970 Å). Drawing from the crystal structure of (μ-**L3**)(Cu^II^Br_2_)_2_, we propose that the in-situ generated Cu^I^Br complex of **L2** and **L3** also adopts a comparable dinuclear structure. Our attempts to obtain single crystals from a mixture of CuBr and **L1** under similar conditions have not been successful. Consequently, the nature of the CuBr/**L1** structure, whether mono- or bis-chelate, remains uncertain.

**Figure 6:**
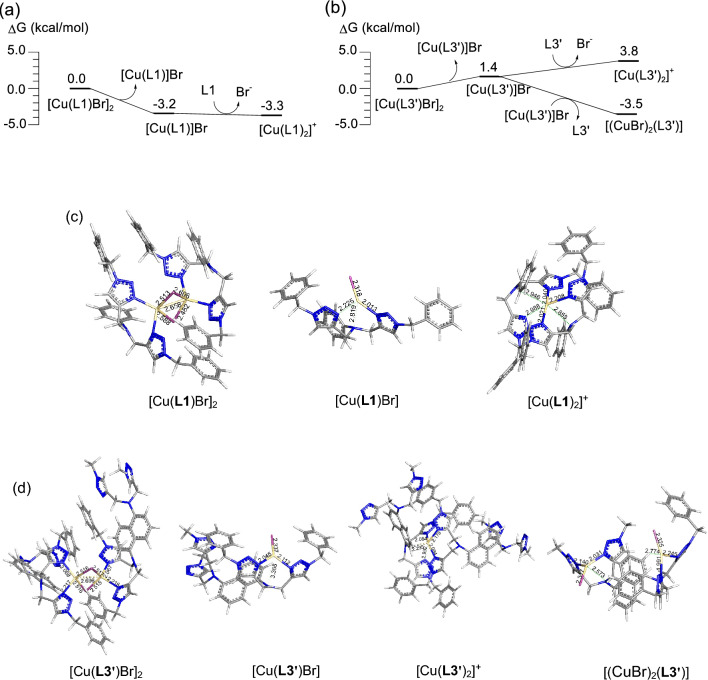
Solvent-corrected relative free energies of possible complexes for (**a**) CuBr/**L1** and (**b**) CuBr/**L3**′. Optimized geometries of possible complexes of (**c**) CuBr/**L1** and (**d**) CuBr/**L3**′.

To further elucidate the structures of Cu^I^Br complexes of **L1** and **L3**, computational studies were carried out using ωB97XD^20^ functional and def2-SVP^21^ basis set for geometry optimizations and using ωB97XD/def2-TZVPP for single-point energy correction in DMSO solvent. Two phenyl rings of **L3** were replaced with hydrogen atoms to simplify the structure (**L3**′). According to the calculations, solvent-corrected relative free energies of the mono- and bis-chelate complexes Cu(**L1**)Br and [Cu(**L1**)_2_]^+^ are comparable. Consistent with our hypothesis, with the **L3** ligand, the dinuclear Cu(I) complex supported by **L3**′ i.e., [(CuBr)_2_(**L3**′)] is the most thermodynamically stable structure and ca. 7.3 kcal/mol more stable than the bis-chelate complex [Cu(**L3**′)_2_]^+^ (Fig. 6).

**Figure 7:**
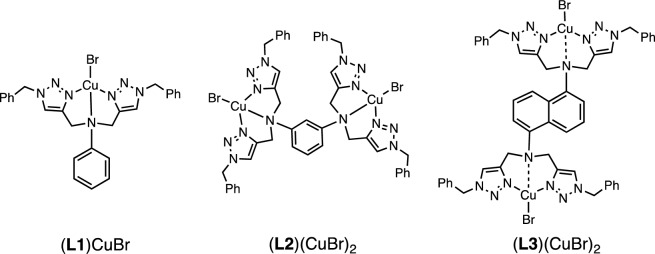
Proposed structures of CuBr/**Ln.**

The stoichiometric ratios between CuBr and **L1**–**L3** were also determined via UV–vis spectrophotometric titrations. For these measurements, DMSO was selected as the solvent due to better solubility of CuBr/**Ln** complexes (**Ln** = **L2** and **L3**) in DMSO compared to CH_3_CN. Absorption data at wavelengths 260, 255, and 258 nm were collected and analyzed using the BindFit program^22–24^ to obtain the binding constant(s) for the CuBr complexes of **L1**, **L2**, and **L3**, respectively. The analyses suggest stoichiometric ratios of CuBr:**Ln** as 1:1 for **L1**, and 2:1 for CuBr:**L2** and CuBr:**L3**, leading to the determination of the binding constants (*K*) as shown in Table 1. Unsurprisingly, the ligand **L3**, which possesses a more electron-delocalized naphthalene-based framework, exhibited the weakest binding strength with CuBr, most likely a result of weak Cu–N_amine_ interactions in DMSO. By integrating information from crystal structures, computational study, and UV–vis titrations, proposed structures for CuBr/**Ln** are illustrated in Fig. 7.

**Table 1:** Binding constants.

CuBr/Ln	Ratio	Mode	*K* (M^−1^)
CuBr/**L1**	1:1	–	900
CuBr/**L2**	2:1	Statistical	5277, 1319
CuBr/**L3**	2:1	Statistical	496, 124

## Catalytic aerobic alcohol oxidation activities

The impact of ligands with single and dual metal binding sites on catalytic alcohol oxidation activities were investigated using benzyl alcohol as the model substrate. We observed that the isolated Cu(II) complex **3** (2.5 mol%) from a reaction between Cu(NO_3_)_2_ and **L3**, combined with TEMPO (5 mol%) and NMI (10 mol%), exhibited low catalytic activities towards aerobic oxidation of benzyl alcohol in CH_3_CN at room temperature, leading to 70% conversion to benzaldehyde at 24 h (entry 1, Table 2). In contrast, in-situ generated CuBr complexes of **L1**–**L3** were more active catalysts and promoted complete oxidation to benzaldehyde at 2 h, under the same conditions (entries 2–4). The ligand **L3**, which features two copper binding sites and a naphthalene linker, afforded the most active copper catalyst system with 71% conversion of benzyl alcohol at 0.5 h (TOF = 57 h^−1^), followed by **L2** (58%) and **L1** (32%), respectively. It should be noted that a mixture of CuBr:**L3** resulted in a small amount of insoluble, off-white solids, which were filtered out to maintain a homogeneous catalyst system. We characterized the insoluble white solids by ^1^H NMR, FT-IR, and ESI–MS, which revealed the presence of the ligand **L3**, which has low solubility in CH_3_CN, along with a trace amount of CuBr (Figs. S21–S23).

**Table 2:** Catalyst comparison for aerobic alcohol oxidation^a,b^


Entry	Cu	Ligand	mol% Cu	Cu:Ln ratio	Time (h)	% Conversion^c^	TOF^d^
1	**3**	–	2.5	–	24	70	1
2	CuBr	**L1**	2.5	1:1	0.5	32	26
					1	68	27
					2	> 99	46
3	CuBr	**L2**	2.5	2:1	0.5	58	46
					1	90	36
					2	> 99	20
4	CuBr	**L3**	2.5	2:1	0.5	71	57
					1	96	38
					2	> 99	20
5	CuBr	**L3**	1.0	2:1	2	49	25
6	CuBr	–	2.5	–	1	63	25
					2	> 99	20
7	–	**L3**^e^	–	–	2	0	0
8^f^	CuBr	**bpy**	2.5	1:1	0.5	51	41
					1	92	37
					2	> 99	20
9	CuBr_2_	**L3**	2.5	2:1	2	4	1
					24	> 99	2

^a^Reaction conditions: benzyl alcohol (1.0 mmol), CuBr (0.025 mmol), **Ln** (0.025 or 0.0125 mmol), TEMPO (0.050 mmol), NMI (0.10 mmol) in CH_3_CN (5 ml) under aerobic conditions with 0.010 mmol of anisole as an internal standard, room temperature. ^b^% conversion determined by GC-MS, average of at least two runs. ^c^% Selectivity > 99%. ^d^TOF (h^−1^) = [% conversion/(mol% Cu × time)]. ^e^**L3** (0.0125 mmol). ^f^CuBr (0.025 mmol), bpy (0.025 mmol).

**Figure 8:**
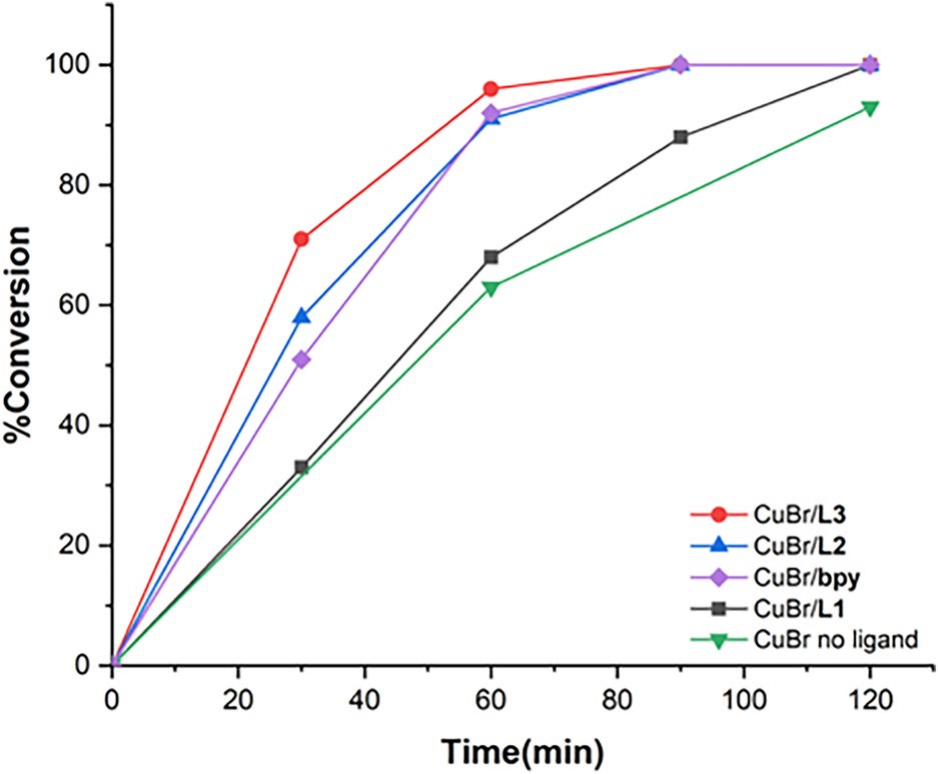
Reactivity profile comparing catalytic oxidation activities of CuBr/**Ln** (**Ln** = **L1**–**L3**, bpy, and no ligand).

The catalytic reaction utilized 2.5 mol% CuBr with 2.5 mol% **L1** (1:1 ratio of CuBr:**L1**) or 1.25 mol% **L2** and **L3** (2:1 ratio of CuBr:**L2** and **L3**). Notably, changing the CuBr:**L3** ratio from 2:1 to 1:1 had no discernible effect on catalytic activity as both ratios provided similar conversions of benzyl alcohol to benzaldehyde after 1 h. Decreasing the amount of CuBr/**L3** to 1.0 mol% resulted in 49% conversion of benzyl alcohol to benzaldehyde after 2 h (entry 5, Table 2). In the absence of **Ln**, the CuBr catalyst stabilized by NMI showed moderate activity, leading to a 63% conversion at 1 h (TOF = 25 h^−1^, entry 6). On the other hand, without CuBr, the ligand **L3** alone did not exhibit catalytic oxidation activity at 2 h under the same conditions (entry 7). Meanwhile, the well-known catalyst system CuBr/bpy (bpy = 2,2′-bipyridine), generated from a 1:1 mixture of CuBr:bpy, resulted in 51% conversion of benzyl alcohol after 0.5 h (TOF = 41 h^−1^, entry 8). Based on catalytic studies, the activity for Cu-catalyzed aerobic oxidation of benzyl alcohol follows this trend: **L3** > **L2** ~ **bpy** > **L1** > no ligand, as illustrated in the reactivity profile (Fig. 8). Furthermore, replacing CuBr/**L3** with CuBr_2_/**L3** led to only 4% conversion at 2 h although complete aerobic oxidation of benzyl alcohol was achieved within 24 h (entry 9 and Fig. S14). Reusability study of the CuBr/**L3** catalyst was also conducted where each catalytic run involved the addition of benzyl alcohol substrate, 5 mol% TEMPO, and 10 mol% NMI at the onset. After 2 h, the catalyst maintained excellent conversions (> 98%) for at least 6 reaction cycles without any loss in catalytic performance.

**Scheme 1:**
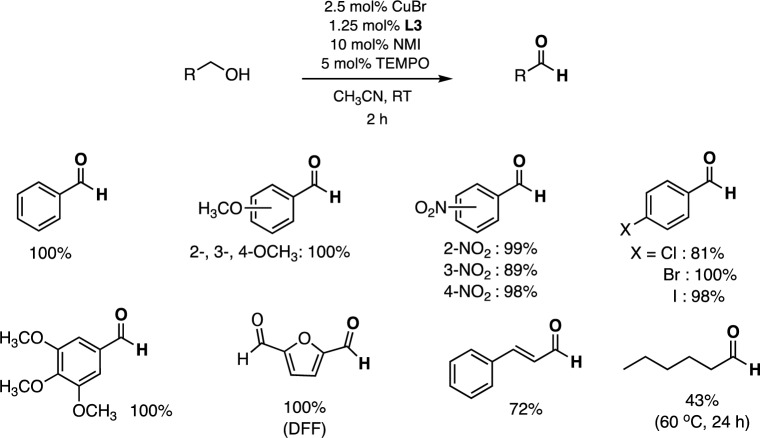
Substrate Scope^a,b^. ^a^Reaction conditions: alcohol substrate (1.0 mmol), CuBr (0.025 mmol), **L3** (0.0125 mmol), NMI (0.10 mmol) and TEMPO (0.050 mmol) in MeCN (5 mL) under aerobic conditions at room temperature with 0.010 mmol of anisole as an internal standard. ^b^% conversion based on GC analysis, average of at least two runs.

The aerobic oxidation activity of the dinuclear catalyst CuBr/**L3** was also compared to those of other multinuclear copper-based catalysts previously reported in the literatures, as shown in Table 3. For aerobic oxidation of benzyl alcohol to benzaldehyde, the catalyst CuBr/**L3**/TEMPO/NMI showed the highest TOF value of 38 h^−1^ under mild conditions. Given its superior catalytic performance, CuBr/**L3** was selected for further substrate scope studies. The catalyst system CuBr/**L3**/TEMPO/NMI in CH_3_CN exhibited high activity for activated primary alcohols at room temperature, resulting in 72–100% conversions and exclusive formation of the corresponding aldehyde products within 2 h (Table 3). Interestingly, under these conditions, the biomass-derived compound 5-hydroxymethyl-2-furfural (HMF) was also completely oxidized to give exclusively 2,5-diformylfuran (DFF) at 2 h, based on GC–MS. It should be noted that electron-deficient activated alcohols such as *m*-NO_2_-C_6_H_4_CH_2_OH, *p*-ClC_6_H_4_CH_2_OH, and cinnamyl alcohol are slower to get oxidized to the aldehyde products, resulting in 89%, 81%, and 72% conversions, respectively^25,26^. On the other hand, aliphatic alcohol such as cyclohexanol and 2-methyl-1-pentanol did not yield any oxidized products. However, 1-hexanol showed low conversion (43%) after 24 h at 60 °C (Scheme 1). Some examples of GC–MS data of the oxidized products were illustrated in Table S4.

**Table 3:** Activity comparison of various multinuclear copper catalysts toward aerobic oxidation of benzyl alcohol^a^*.*

Cu catalyst	Base/solvent	Temp (°C)	Time (h)	% Conversion	TOF (h^−1^)	References
Cu_2_(µ-H_2_tea)_2_{µ_3_-Na_2_(H_2_O_4_)}(µ_6_-pma)]_n_	K_2_CO_3_/H_2_O	50	48	> 99%	2.06	^27^
(tpy)CuCl(µ-Cl)CuCl^b^	DMAP/H_2_O	25	5	99%	19.8	^9^
(µ-Cl)_2_[Cu(bzm)Cl]_2_	–/H_2_O	RT	10	99%	1.98	^28^
(µ-Cl)_2_[Cu(Hpyet)Cl]_2_	Na_2_CO_3_/H_2_O	RT	16	93%	1.16	^29^
(L)[Cu(OCOMe)]_2_^*b*^	K_2_CO_3_/H_2_O	70	20	99%	12.0	^14^
[(µ-Br)Cu_3_(btm)_3_(H_2_O)]Br_2_^*b*^	NMI/EtOH:H_2_O	RT	8	99%	2.48	^8^
Cu_2_-CatPOP-2	–/MeCN	RT	2	80%	4.0	^30^
CuBr/**L3**	NMI/MeCN	RT	1	96%	38.0	This work

^a^The co-catalyst is TEMPO. ^b^Structures were shown in Fig. 1. *Note*: H_3_tea = triethanolamine, H_4_pma = pyromellitic acid, bzm = 1-methylbenzimidazole, Hpyet = 2-N-(picolinylidene)ethanol, Hbtm = bis(1-benzyl-1*H*-1,2,3-triazol-4-yl)phenylmethanol

### Electrochemical properties

**Figure 9:**
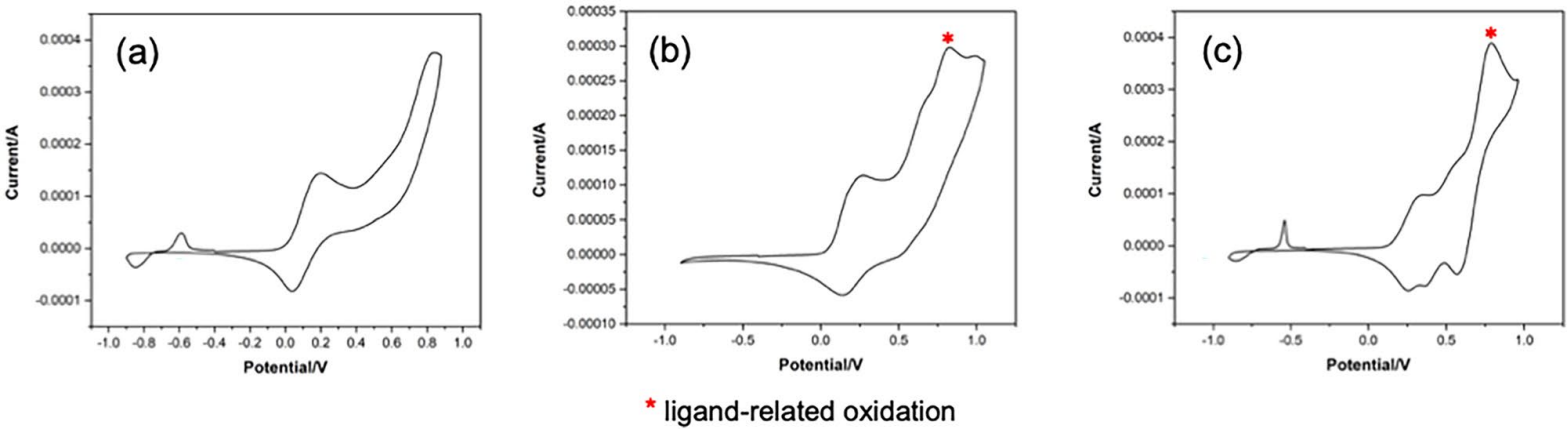
CV data of in-situ generated (**a**) CuBr/**L1**, (**b**) CuBr/**L2**, (**c**) CuBr/**L3** in CH_3_CN under aerobic conditions. Scan rate = 100 mV/s.

**Figure 10:**
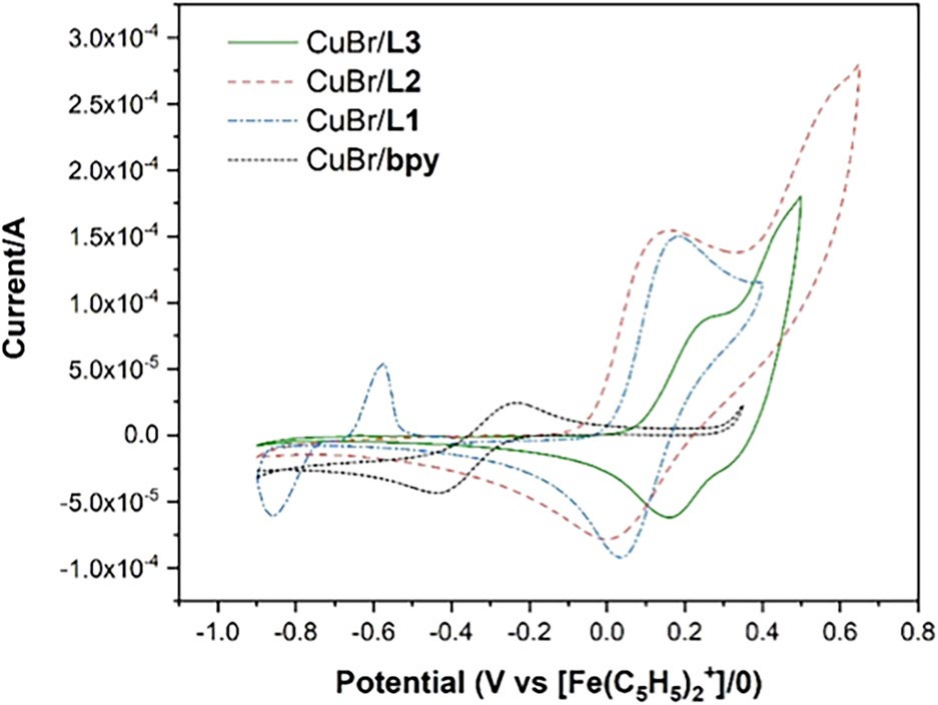
Overlaid CV spectra of CuBr/**Ln** (**Ln** = **L1**–**L3**, bpy) in CH_3_CN under aerobic conditions in the range of -0.9–0.4 V. Scan rate = 100 mV/s.

To gain insights into the oxidation activities of these catalyst systems, we investigated the electrochemical properties of the in-situ generated complexes CuBr/**Ln** (**Ln** = **L1**–**L3**) in CH_3_CN using cyclic voltammetry, with [Bu_4_N]PF_6_ as the supporting electrolyte under aerobic conditions. The mononuclear CuBr/**L1** exhibited a quasi-reversible redox wave with an *E*_1/2_ value of 0.053 V vs [Fe(C_5_H_5_)_2_]^+/0^. On the other hand, the cyclic voltammogram of CuBr/**L2** and CuBr/**L3** revealed two pairs of redox waves corresponding to the redox processes which are the one-electron oxidation of Cu(I)Cu(I) to Cu(II)Cu(I) (**A**) and Cu(II)Cu(I) to Cu(II)Cu(II) (**B**). The first oxidative process of CuBr/**L1** and CuBr/**L2** are comparable (CuBr/**L1**: *E*_1/2_ = 0.053 V versus CuBr/**L2**: *E*_1/2_^**A**^ = 0.114 V), while that of CuBr/**L3** are more positive (*E*_1/2_^**A**^ = 0.261 V), as displayed in Fig. 9. The additional oxidative process was observed for CuBr/**L2** and CuBr/**L3** at 0.778 and 0.728 V, respectively (Fig. 9b,c). Upon comparing these oxidative waves with that of the in-situ generated Zn(OTf)_2_/**Ln** (**Ln** = **L1**–**L3**) complexes under identical conditions, we assigned these peaks to ligand-related oxidation events (Fig. S10). The overlaid CV data comparing the first redox waves of CuBr/**Ln**, and CuBr/bpy are shown in Fig. 10, showing the first oxidative process of CuBr/**Ln** to be more positive than that of CuBr/bpy. Furthermore, the reduction potential *E*_pc_^**A**^, corresponding to the the reduction process of Cu(II)-**Ln**-Cu(I) to Cu(I)-**Ln**-Cu(I), is most positive with the ligand **L3** (*E*_pc_^**A**^ = 0.280 V compared to 0.045 V for **L2**, Table 4). This suggests a more facile reduction of the mixed-valence dinuclear copper complexes in the presence of **L3**.

**Table 4:** Electrochemical data (in V) and *K*_c_ for CuBr/**Ln** (**Ln** = **L1**–**L3**) generated in situ in CH_3_CN relative to the Fc^0/+^ couple in 5 mmol L^−1^ [nBu_4_Br]PF_6_/CH_3_CN in air.

CuBr/**Ln**	*E*_pc_^**A**^	*E*_pa_^**A**^	*E*_pc_^**B**^	*E*_pa_^**B**^	*E*_1/2_^**A**^	*E*_1/2_^**B**^	Δ*E*_pa_^a^	*K*_*c*_^b^
CuBr/**L1**	− 0.040	0.145	–	–	0.053	–	–	N/A
CuBr/**L2**	0.045	0.183	0.659	0.576	0.114	0.618	0.393	4.39 × 10^6^
CuBr/**L3**	0.280	0.242	0.429	0.452	0.261	0.441	0.210	3.54 × 10^3^

^a^Δ*E*_pa_ = *E*_pa_^**B**^ – *E*_pa_^**A**^. ^b^*K*_*c*_ = $\exp \{ nF(\Delta E_{{{\text{pa}}}} )/RT\}$, where *n* = 1, *F*/*RT* = 38.92 V^−1^ at 298 K^31^.

We further determined the stability of the mixed-valence Cu(I)Cu(II) species and electronic communication (coupling) between copper centers via the comproportionation constants (*K*_c_) for CuBr/**L2** and CuBr/**L3** calculated using the expression^31^:

$${\text{K}}_{{\text{c}}} = \frac{{[{\text{Cu}}({\text{I}}){\text{Cu}}({\text{II}})]^{2} }}{{\left[ {{\text{Cu}}({\text{I}}){\text{Cu}}({\text{I}})} \right][{\text{Cu}}({\text{II}}){\text{Cu}}({\text{II}})]}} = \exp \{ nF\left( {\Delta E_{ox} } \right)/RT\}$$

where Δ*E*_ox_ is the separation between the two redox potentials for the successive oxidation processes. We found that the *K*_c_ value of CuBr/**L3** is significantly smaller than that of CuBr/**L2** (3.54 × 10^3^ vs. 4.39 × 10^6^, Table 4), suggesting lower stability of the mixed-valence Cu(I)-**L3**-Cu(II) species and less electronic communication between the copper centers. Less stable Cu(I)-**L3**-Cu(II) species are also a result of weaker σ interactions between Cu–N_amine_ based on smaller CuBr-**L3** binding constants (Table 1), larger bridging π systems, and longer Cu···Cu distance (i.e., 10.83 Å for **4** cf. 8.12 Å for **2**).

**Figure 11:**
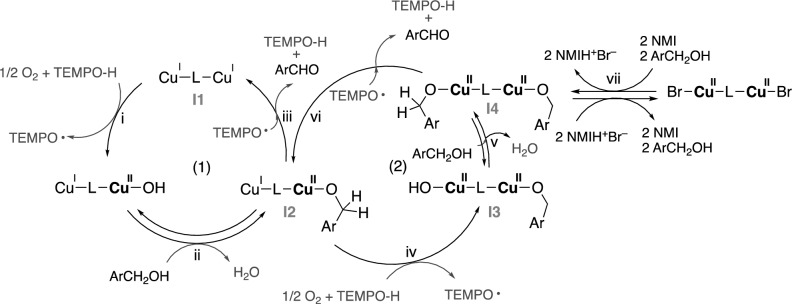
Proposed mechanism involving Cu(I)Cu(I)/Cu(I)Cu(II)/Cu(II)Cu(II) catalytic species.

Based on catalytic results, characterization data, and established mechanisms for mononuclear Cu-catalyzed alcohol oxidation^32,33^, we propose a mechanism for the aerobic alcohol oxidation catalyzed by dinuclear copper catalysts, as shown in Fig. 11. Cycle (1), which involves Cu(I)Cu(I) and Cu(I)Cu(II) species, is believed to be the primary catalytic cycle, as the CuBr-based catalyst system exhibits significantly higher activity than related Cu(II) species (vide supra). Meanwhile, the Cu(II)-**Ln**-Cu(II) catalyst can also contribute to the catalytic oxidation activity, since the CuBr_2_/**L3** catalyst was found to be active, although with much lower oxidation activity (entry 8, Table 2). The higher catalytic performance of CuBr/**L3** compared to CuBr/**L2** is attributed to the lower stability of the mixed-valence Cu(I)-**L3**-Cu(II) species **I2**. This instability is expected to facilitate a more facile reduction from **I2** to **I1** and the aldehyde product (step iii) consistent with the more positive *E*_pc_^**A**^ value of CuBr/**L3** (0.280 V) compared to that of CuBr/**L2** (0.045 V), as outlined in Table 4.

## Conclusion

In this study, we synthesized a set of of amine-bis(triazole) ligands, containing a single metal binding site **L1** and dual metal binding sites **L2** and **L3** with 1,3-phenylene and 1,5-naphthalene linkers, respectively. Using these ligands, the corresponding mononuclear and multinuclear Cu(II) complexes were successfully generated. X-ray crystallography revealed the formation of one-dimensional coordination polymers and dinuclear Cu(II) complexes with the **L2** and **L3** ligands. Data obtained from DFT calculations, UV–vis titrations, and the crystal structure of (μ-**L3**)(CuBr_2_)_2_ supported the proposed formulations of the mononuclear and dinuclear CuBr complexes: CuBr(**L1**) and (μ-**Ln**)(CuBr)_2_ (**Ln** = **L2** and **L3**). Catalytic studies on Cu-catalyzed aerobic oxidation of benzyl alcohol to benzaldehyde demonstrated that the in-situ generated CuBr/**L3** catalyst, combined with TEMPO and NMI, exhibited the highest activity. Moreover, dinuclear copper catalysts supported by **L2** and **L3** ligands generally showed greater activity than the related mononuclear copper catalyst CuBr/**L1**. The superior oxidation activity of CuBr/**L3** was attributed to the lower stability and higher reducibility of the mixed-valence Cu(I)-**L3**-Cu(II) intermediate compared to Cu(I)-**L2**-Cu(II), as evidenced by cyclic voltammetry (CV) data. In summary, this work highlights the superior oxidation activities of dinuclear Cu catalysts and demonstrates the impact of aromatic linkers in amine-bis(triazolyl) chelates. Specifically, the extended π-conjugated ligand system of the 1,5-naphthalene linker resulted in weaker Cu–L binding and longer Cu···Cu distances compared to the 1,3-phenylene linker, as indicated by UV–vis and X-ray crystallographic data. This weaker Cu–L binding most likely contributes to lower stability of mixed-valence Cu(I)-**L3**-Cu(II) intermediates and consequently higher oxidation activity.

### Supplementary Information

Supplementary Information.

## Data Availability

Crystallographic data for the structures reported in this article have been deposited at the Cambridge Crystallographic Data Center, under the deposition numbers CCDC 2307371 (**1**), 2307372 (**2**), 2307373 (**3**), and 2307512 (**4**) and can be obtained free of charge via http://www.ccdc.cam.ac.uk/structures/. All other relevant data such as experimental, ^1^H NMR, ^13^C{^1^H} NMR spectra (Figs. S1–S3, S5–S7), ESI–MS (Figs. S4, S8), CV (Figs. S9–S11), UV–vis titration data (Figs. S13–S18 and Tables S1–S3), FT-IR (Fig. S19), and DFT calculation detail (Fig. S22 and ESI pages 20–42) are included in this article and its supplementary information.
