# Supplementary material for: Effects of the ligand linkers on stability of mixed-valence Cu(I)Cu(II) and catalytic aerobic alcohol oxidation activity
Source: Sci Rep. 2024 Jul 5;14:15497. doi: 10.1038/s41598-024-66227-2 (PMC11226613; doi:10.1038/s41598-024-66227-2)
Supplement: Supplementary file 1 — Supplementary Information. [file 41598_2024_66227_MOESM1_ESM.docx]

**Supplementary Material**

**Effects of Ligand Structures on Aerobic Alcohol Oxidation Activity of Multinuclear Copper Catalysts**

Attawit Jehdaramarn,*^a^* Teera Chantarojsiri,*^a^* Thanapat Worakul,*^a^* Panida Surawatanawong,*^a,b^* Kittipong Chainok,*^c^* Preeyanuch Sangtrirutnugul**^a^*

* Corresponding author. Email: preeyanuch.san@mahidol.edu

*^a^*Center of Excellence for Innovation in Chemistry (PERCH-CIC), Department of Chemistry, Faculty of Science, Mahidol University, Bangkok, Thailand.

*^b^*Center of Sustainable Energy and Green Materials, Mahidol University, Salaya, Nakhon Pathom 73170, Thailand

*^c^*Thammasat University Research Unit in Multifunctional Crystalline Materials and Applications (TU-MCMA), Faculty of Science and Technology, Thammasat University, Pathum Thani 12121, Thailand

**Table of Contents**

| **Contents** | **Page** |
| --- | --- |

**Figure S1.** ^1^H and ^13^C{^1^H} spectra NMR of compound **DP1** in CDCl_3_. 4

**Figure S2.** ^1^H and ^13^C{^1^H} spectra NMR of compound **TP2** in CDCl_3_ 5

**Figure S3.** ^1^H and ^13^C{^1^H} spectra NMR of compound **TP3** in CDCl_3_ 6

**Figure S4.** ESI-MS spectra of compounds **TP2** (a) and **TP3** (b) 7

**Figure S5.** ^1^H NMR and ^13^C{^1^H} NMR spectra of **L1** in DMSO-*d*_6_.. 8

**Figure S6.** ^1^H NMR and ^13^C{^1^H} NMR spectra of **L2** in DMSO-*d*_6_. 9

**Figure S7.** ^1^H NMR and ^13^C{^1^H} NMR spectra of **L3** in DMSO-*d*_6_. 10

**Figure S8.** ESI-MS spectra of **L2** (a) and **L3** (b). 11

**Figure S9.** FT-IR spectra of **TP2** and **L2** 12

**Figure S10.** FT-IR spectra of **TP3** and **L3** 12

**X-ray Crystallography Detail**. 13

**Table S1.** Crystal data of complexes **1**–**4**. 14

**Figure S11.** Overlaid CV of CuBr/**Ln** and **Ln** where **Ln** = **L1** (a), **L2** (b), and **L3** (c)……………………………………………………………………………………………………………………………………………………15

**Figure S12.** Overlaid CV data of CuBr/**Ln** and Zn(OTf)_2_/**Ln** where **Ln** = **L1** (a), **L2** (b), and **L3** (c). 15

**Figure S13.** Square root scan rate *vs*. current of CuBr/**Ln** where **Ln** = **L1** (a), **L2** (b), **L3** (c). 16

**Figure S14.** Oxidation profile of benzyl alcohol conversion to benzaldehyde by CuBr/**L3** and CuBr_2_/**L3**. 16

**Figure S15.** Absorbance spectra of CuBr at 0.0–10.0 equivalents in 1.0 x 10^-4^ M **L1**(aq.). 17

**Figure S16.** Binding isotherms from UV-Vis titrations of **L1** with CuBr.. 17

**Table S2.** Summary of BindFit analysis from UV-Vis titrations between **L1** and CuBr 17

**Figure S17.** Absorbance spectra of CuBr at 0.0–10.0 equivalents in 1.0 x 10^-4^ M **L2** (aq.). 18

**Figure S18.** Binding isotherms from UV-Vis titrations of **L2** with CuBr. 18

**Table S3.** Summary of BindFit analysis from UV-Vis titrations between **L2** and CuBr. 18

**Figure S19.** Absorbance spectra of CuBr at 0.0–10.0 equivalents in 1.0 x 10^-4^ M **L3**(aq.). 19

**Figure S20.** Binding isotherms from UV-Vis titrations of **L3** with CuBr.. 19

**Table S4.** Summary of BindFit analysis from UV-Vis titrations between **L3** and CuBr. 19

**Table S5.** Examples of GC-MS data from aerobic oxidation of various alcohols by CuBr/**L3**/NMI/TEMPO in CH_3_CN. 20-22

**Figure S21.** FT-IR spectra of CuBr, free ligand **L3**, and off-white solids 23

**Figure S22.** ^1^H NMR spectra of off-white solids and **L3** 23

**Figure S23.** ESI-MS spectra of off-white solids 24

**Figure S24.** Optimized geometries of **L1** and **L3’**. 25

**References** 26

**Cartesian Coordinates** 27-47


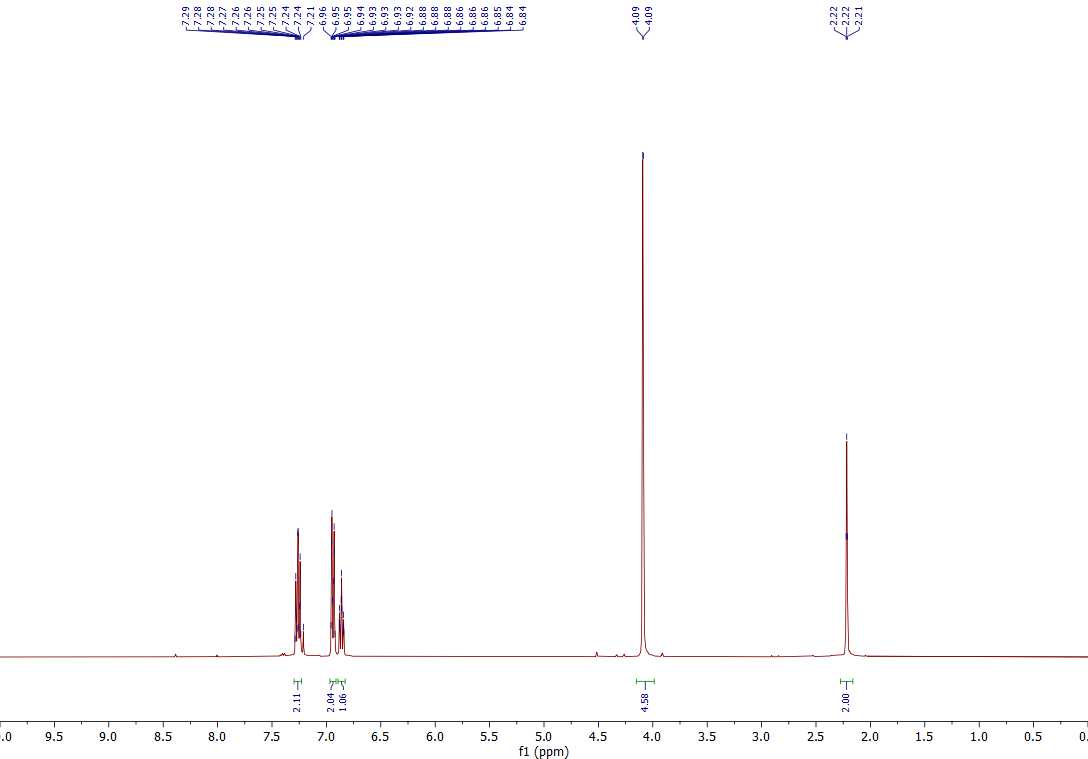

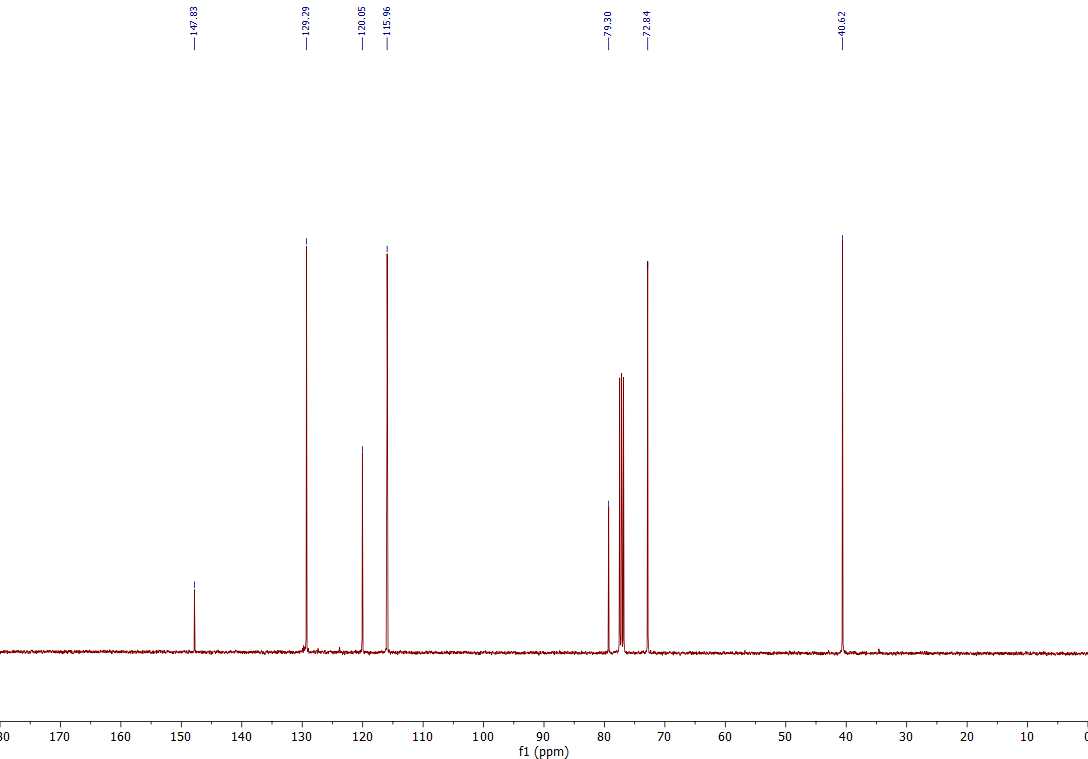


**Figure S1.** ^1^H and ^13^C{^1^H} spectra NMR of compound **DP1** in CDCl_3_


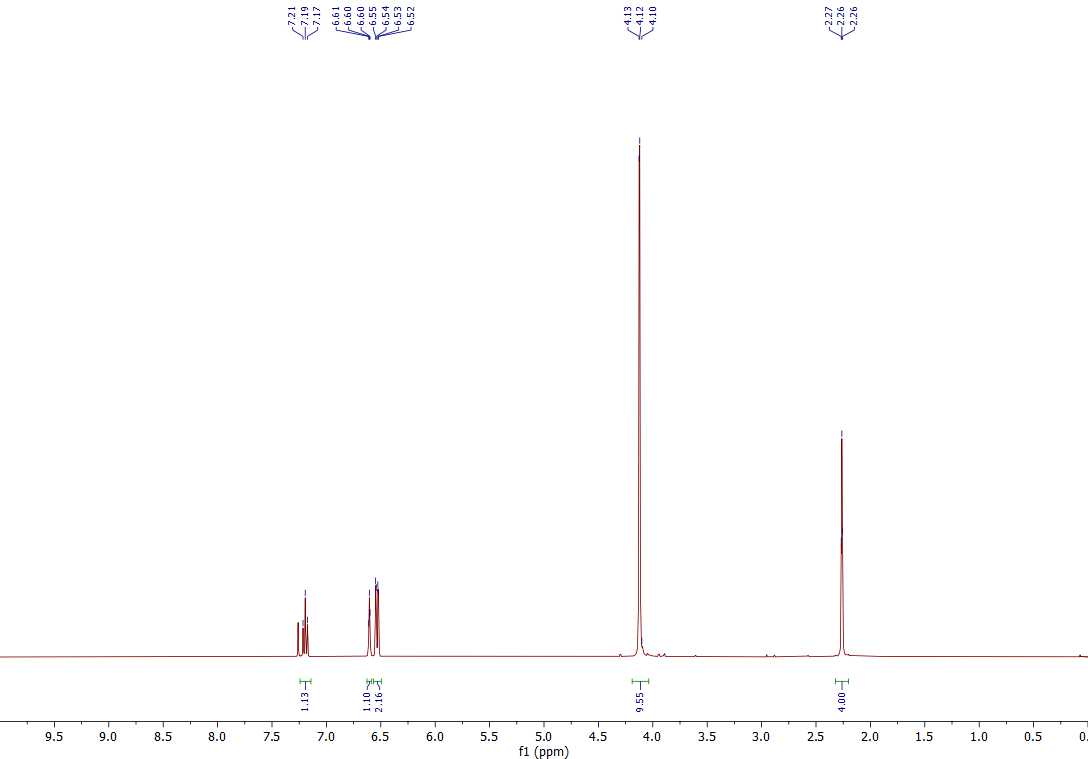

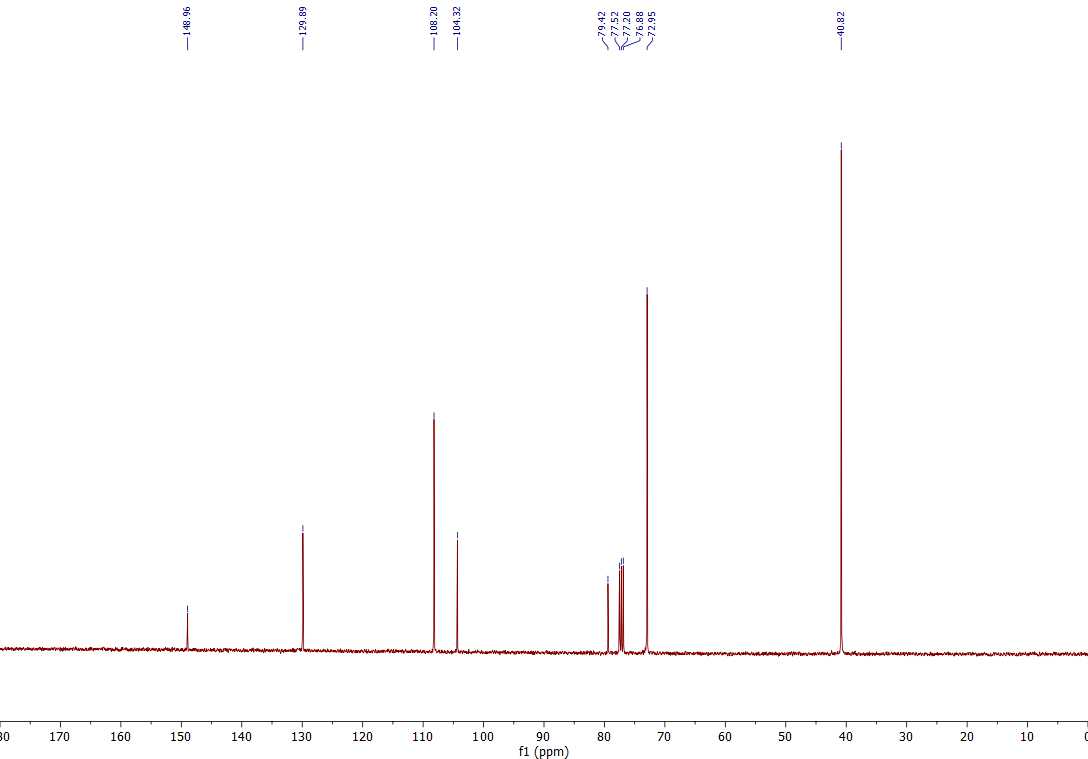


**Figure S2.** ^1^H and ^13^C{^1^H} spectra NMR of compound **TP2** in CDCl_3_


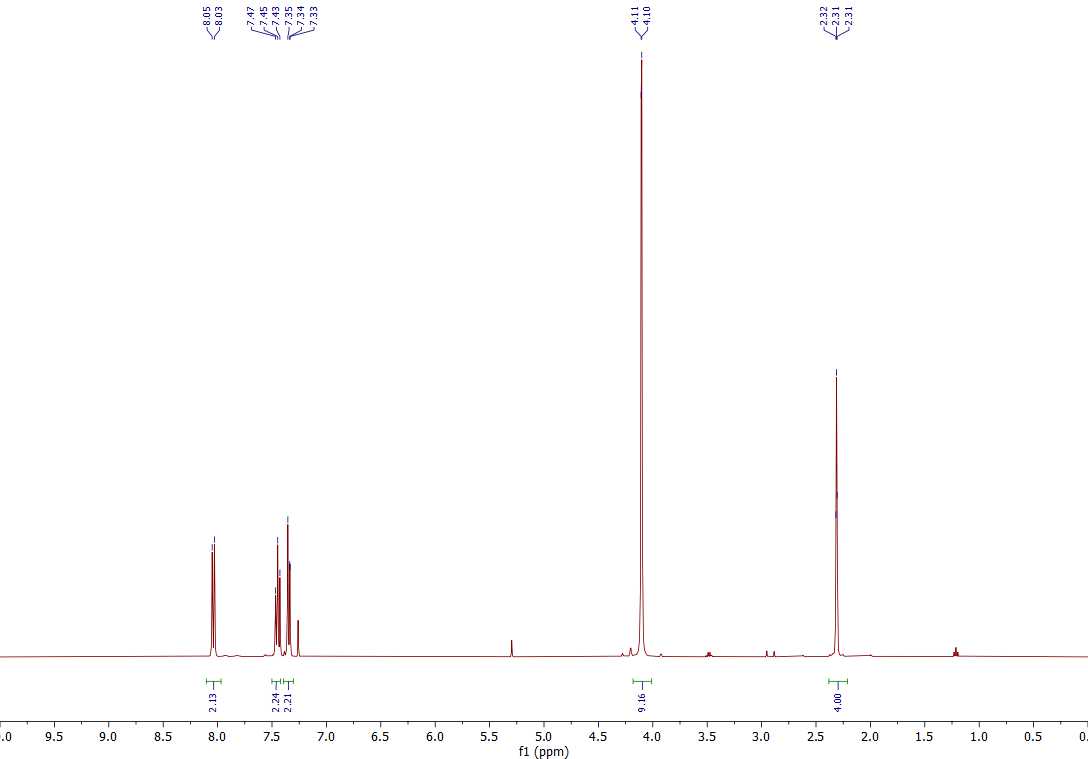

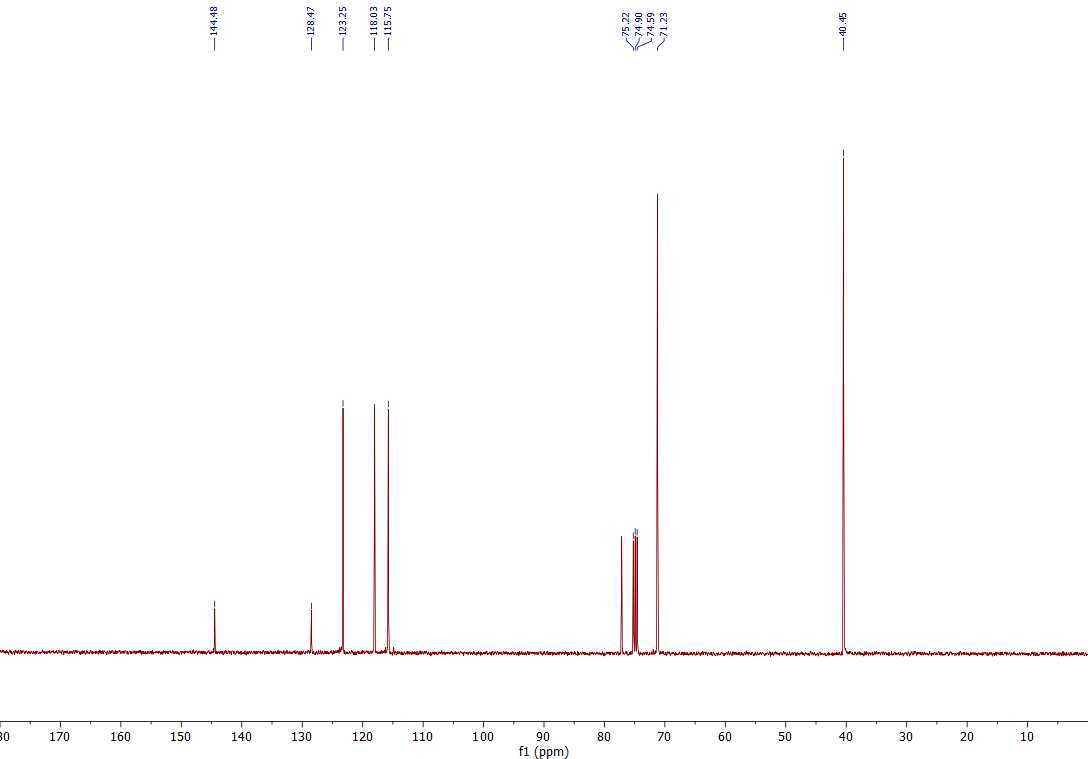


**Figure S3.** ^1^H and ^13^C{^1^H} spectra NMR of compound **TP3** in CDCl_3_

(a)

(b)

**Figure S4.** ESI-MS spectra of compounds **TP2** (a) and **TP3** (b)


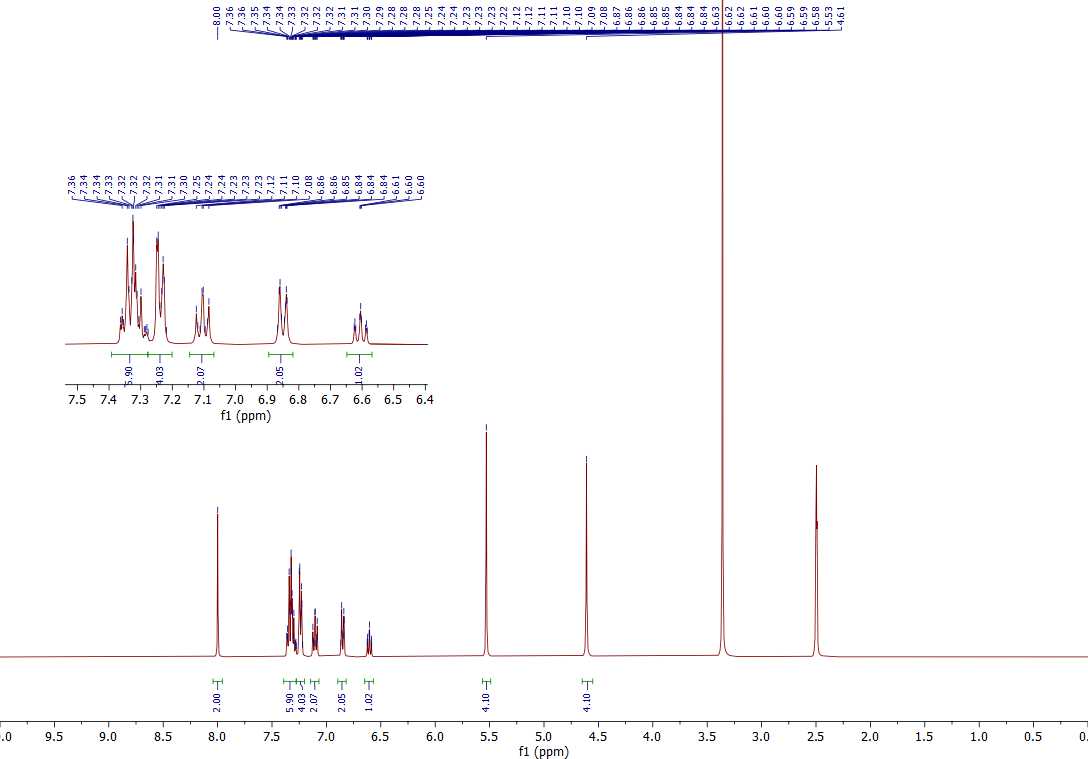

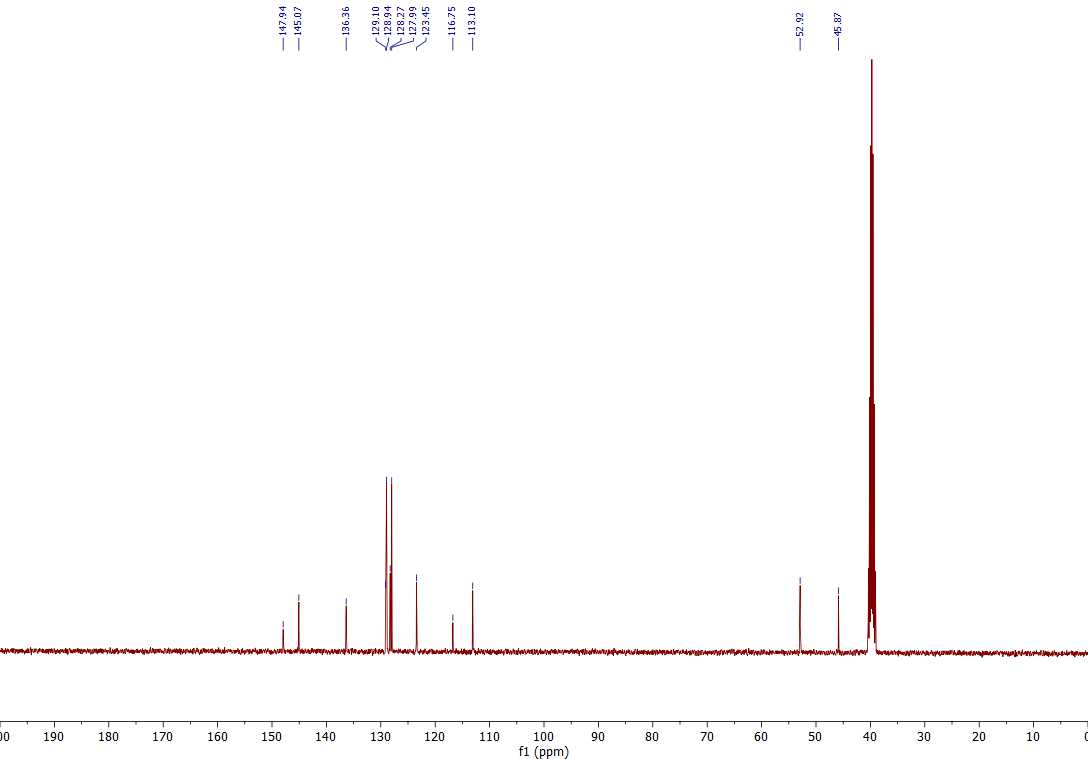


**Figure S5.** ^1^H NMR and ^13^C{^1^H} NMR spectrum of **L1** in DMSO-*d*_6_.


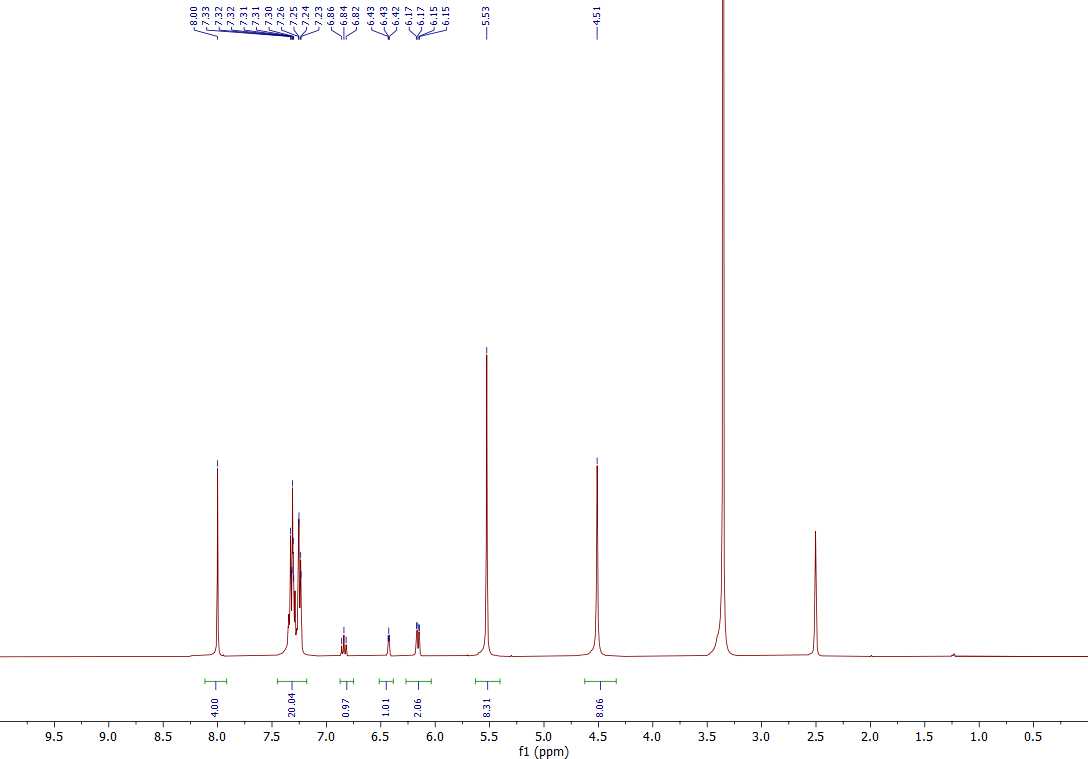


_
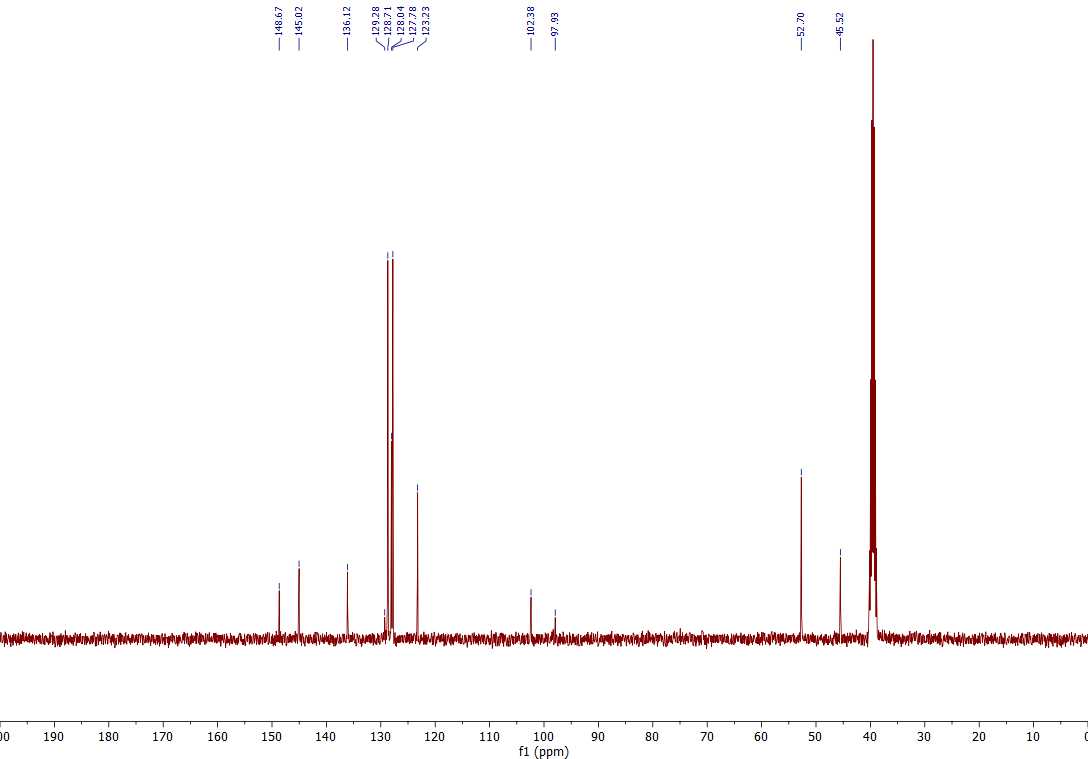
_

**Figure S6.** ^1^H NMR and ^13^C{^1^H} NMR spectrum of **L2** in DMSO-*d*_6_.

_
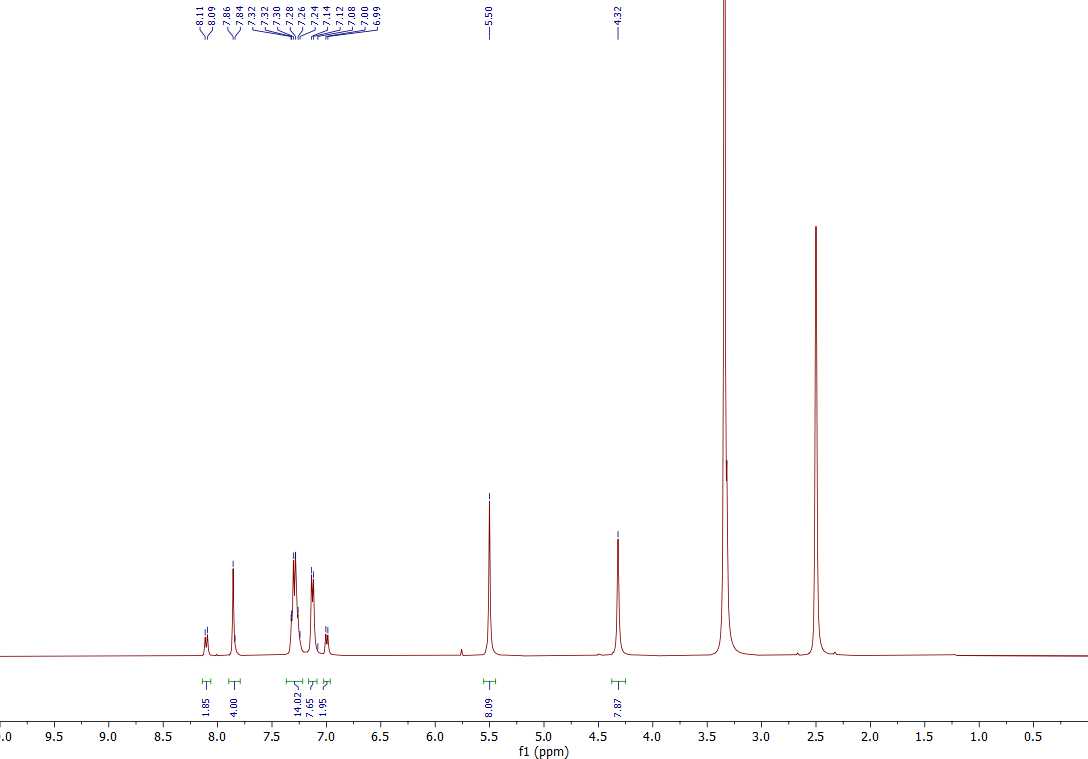
_

_
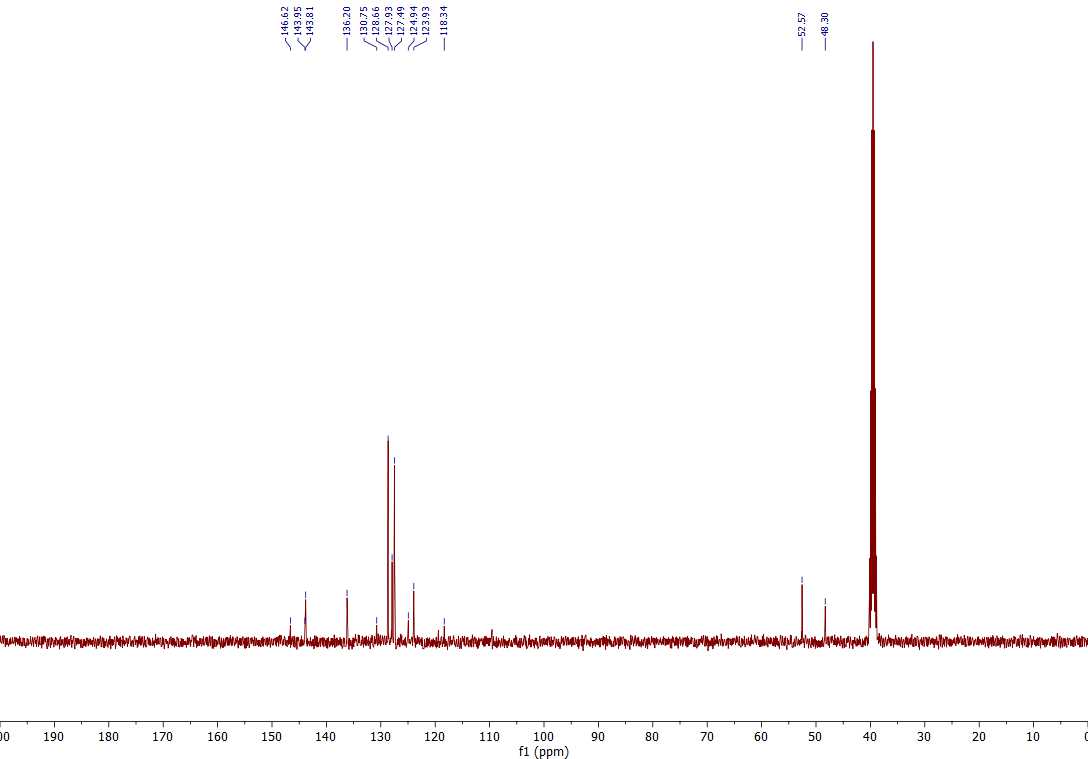
_

**Figure S7.** ^1^H NMR and ^13^C{^1^H} NMR spectrum of **L3** in DMSO-*d*_6_.

(a)

(b)

**Figure S8.** ESI-MS spectra of **L2** (a) and **L3** (b)

**
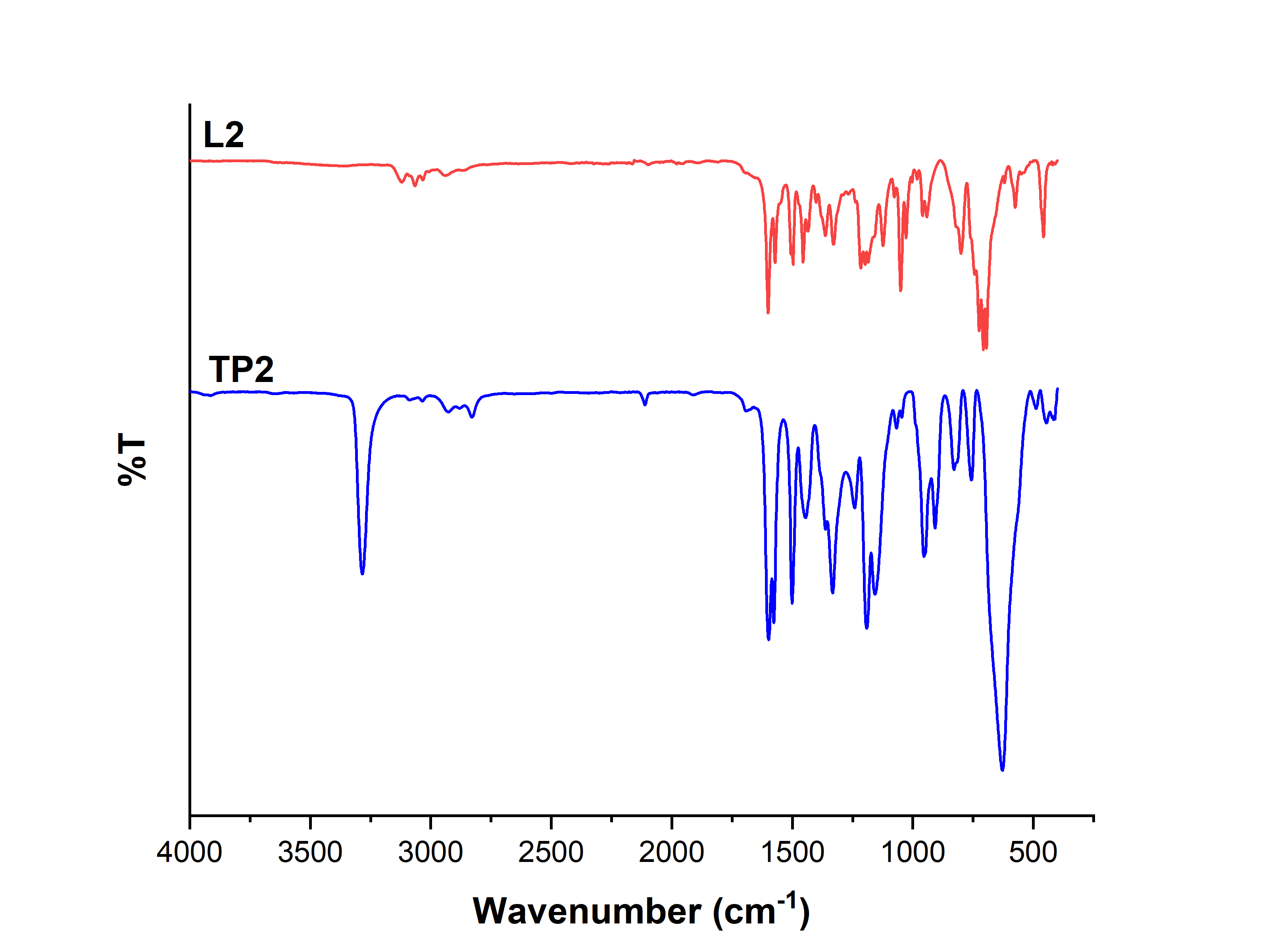
**

**Figure S9.** FT-IR spectra of **TP2** and **L2**

**
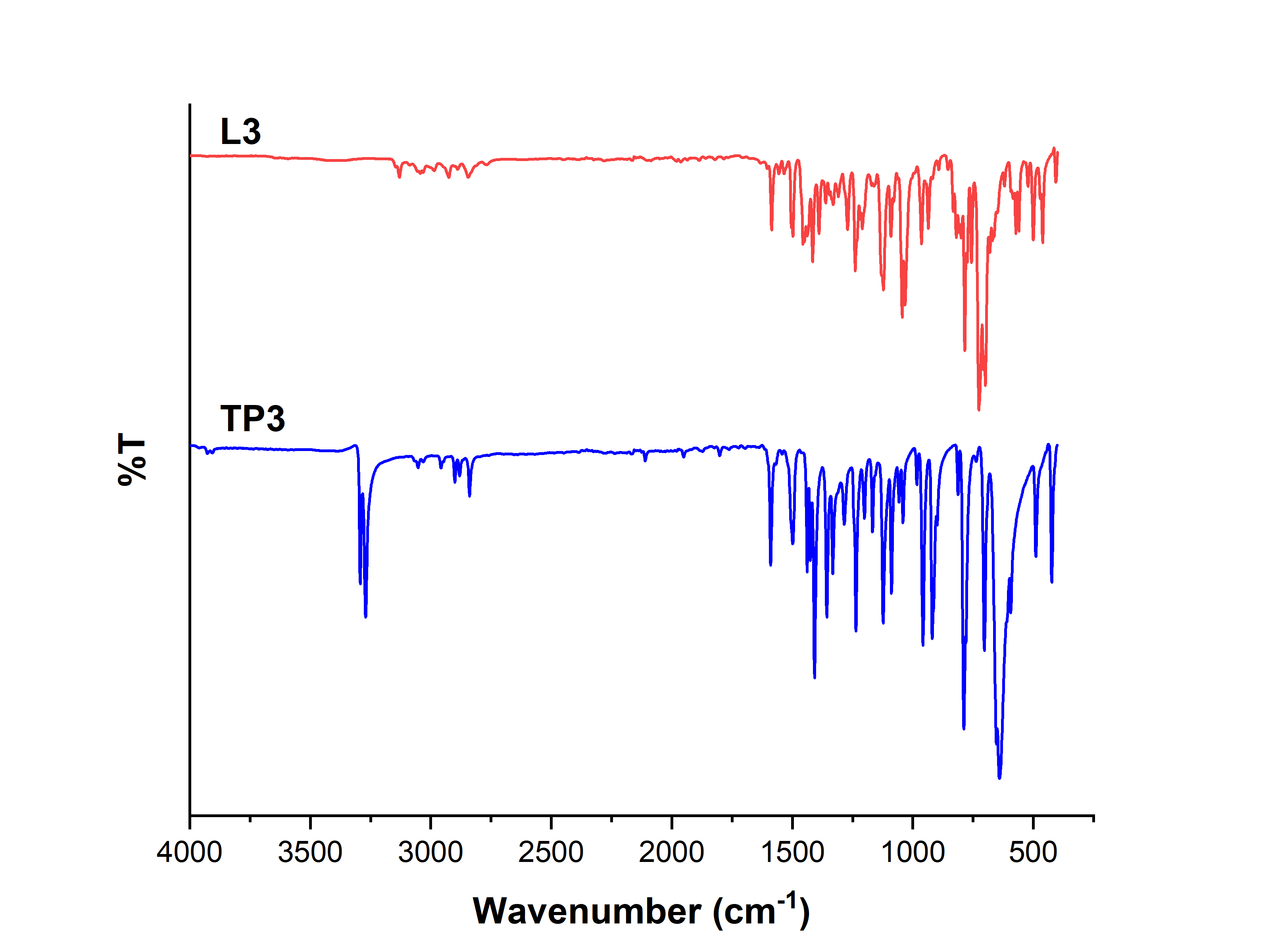
**

**Figure S10.** FT-IR spectra of **TP3** and **L3**

**X-Ray Crystallography Detail**

The crystals of complexes **1**–**4** were mounted on MiTeGen micromounts using Paratone oil, obtained from Hampton Research. The Bruker D8 QUEST CMOS PHOTON II diffractometer (λ_Mo_ = 0.71073 Å) was employed to obtain X-ray diffraction data for **1** and **4** at a temperature of 296 K, while the Bruker D8 Venture CMOS PHOTON I diffractometer (λ_Cu_ = 1.54178 Å) was utilised at temperatures of 100 K and 296 K for complexes **2** and **3**, respectively. The data were obtained through the use of ω and ϕ scans. The determination of the total number of runs and images was derived from the strategic calculation performed by the APEX4 programme. The refinement of unit cell indexing was carried out *via* the SAINT software. The process of data reduction involved the utilisation of SAINT for data integration and scaling, while absorption correction was carried out using SADABS.^[1]^ The integrity of the symmetry was assessed using the utilisation of PLATON.^[2]^ The structure was determined using the ShelXT structure solution programme, which used a combination of Patterson and dual-space recycling approaches.^[3]^ The structure refinement process was conducted using the intrinsic phasing with the ShelXL software ^[4]^ implemented within the OLEX2 programme.^[5]^ The details of crystallographic parameters, data collection and refinements for **1**–**4** are listed in **Table S1**. Crystallographic data (excluding structure factors) for the structures in this paper have been deposited with the Cambridge Crystallographic Data Centre, CCDC, 12 Union Road, Cambridge CB21EZ, UK. Copies of the data can be obtained free of charge on quoting the depository numbers CCDC-2307371-2307373 for **1**–**3** and CCDC-2307512 for **4**, respectively. E-Mail: deposit@ccdc.cam.ac.uk, http://www.ccdc.cam.ac.uk).

References

[1] Bruker, *APEX4, SAINT and SADABS*, Bruker AXS Inc., Madison, Wisconsin, USA, **2021**.

[2] A. L. Spek, *Acta Crystallogr., Sect. C: Struct. Chem.* **2015***, 71,* 9−18.

[3] G. M. Sheldrick, *Acta Crystallogr., Sect. A Found. Adv*. **2015**, *71*, 3−8.

[4] G. M. Sheldrick, *Acta Crystallogr., Sect. A Found. Adv*. **2015**, *71*, 3−8.

[5] O. V. Dolomanov, L. J. Bourhis, R. J. Gildea, J. A. K. Howard, H. Puschmann, *J. Appl. Cryst*. **2009**, *42*, 339−341.

**Table S1.** Crystal data and details of structure refinement for **1**–**4**.

|  | **1** | **2** | **3** | **4** |
| --- | --- | --- | --- | --- |
| Chemical formula | C_54_H_58_CuN_16_O_8_ | C_52_H_53_Cu_2_N_21_O_12_ | C_50_H_46_CuN_16_O_6_ | C_50_H_46_CuN_14_Br_4_ |
| Fw | 1122.70 | 1291.23 | 1030.57 | 1289.73 |
| Crystal system | Triclinic | Triclinic | Monoclinic | Orthorhombic |
| Space group | *P*-1 | *P*-1 | *P*2_1_/*n* | *Pbca* |
| Temperature (*K*) | 296(2) | 100(2) | 296(2) | 296(2) |
| *a* (Å) | 9.789(3) | 13.2815(15) | 12.9521(5) | 17.3512(6) |
| *b* (Å) | 10.362(3) | 14.5779(16) | 27.2542(9) | 13.5680(4) |
| *c* (Å) | 14.339(4) | 16.2984(19) | 14.5597(6) | 21.2409(7) |
| *α* (°) | 69.364(11) | 90.809(4) | 90 | 90 |
| *β* (°) | 89.783(12) | 102.228(4) | 100.350(2) | 90 |
| *γ* (°) | 81.522(11) | 108.331(4) | 90 | 90 |
| *V* (Å^3^) | 1096.68(4) | 2916.4(6) | 5055.9(3) | 5000.6(3) |
| *Z* | 1 | 2 | 4 | 4 |
| *D*_calc_ (g cm^−3^) | 1.387 | 1.470 | 1.354 | 1.713 |
| Radiation | Mo *Kα*  (λ = 0.71073 Å) | Cu *Kα*  (λ = 1.54178 Å) | Cu *Kα*  (λ = 1.54178 Å) | Mo *Kα*  (λ = 0.71073 Å) |
| 2*θ* for data collection (°) | 6.04–53.32 | 7.20–136.47 | 6.49–145.13 | 5.41–52.53 |
| Reflections collected | 13004 | 112052 | 58990 | 6204 |
| Unique Reflections | 5417 | 10506 | 9896 | 4268 |
| GOF on *F*^2^, *S* | 1.051 | 1.049 | 1.029 | 1.027 |
| *R*_1_, *wR*_2_ (*I* > 2*σ*(*I*)) | 0.0467, 0.1175 | 0.0462, 0.1357 | 0.0844, 0.2211 | 0.0446, 0.0934 |
| *R*_1_, *wR*_2_ (all data) | 0.0606, 0.1260 | 0.0475, 0.1368 | 0.1233, 0.2647 | 0.0784, 0.1068 |
| Δ*ρ*_max_, Δ*ρ*_min_ (e Å^−3^) | 0.39, −0.31 | 0.68, −0.94 | 0.69, −0.30 | 0.88, –0.79 |
| CCDC No. | 2307371 | 2307372 | 2307373 | 2307512 |


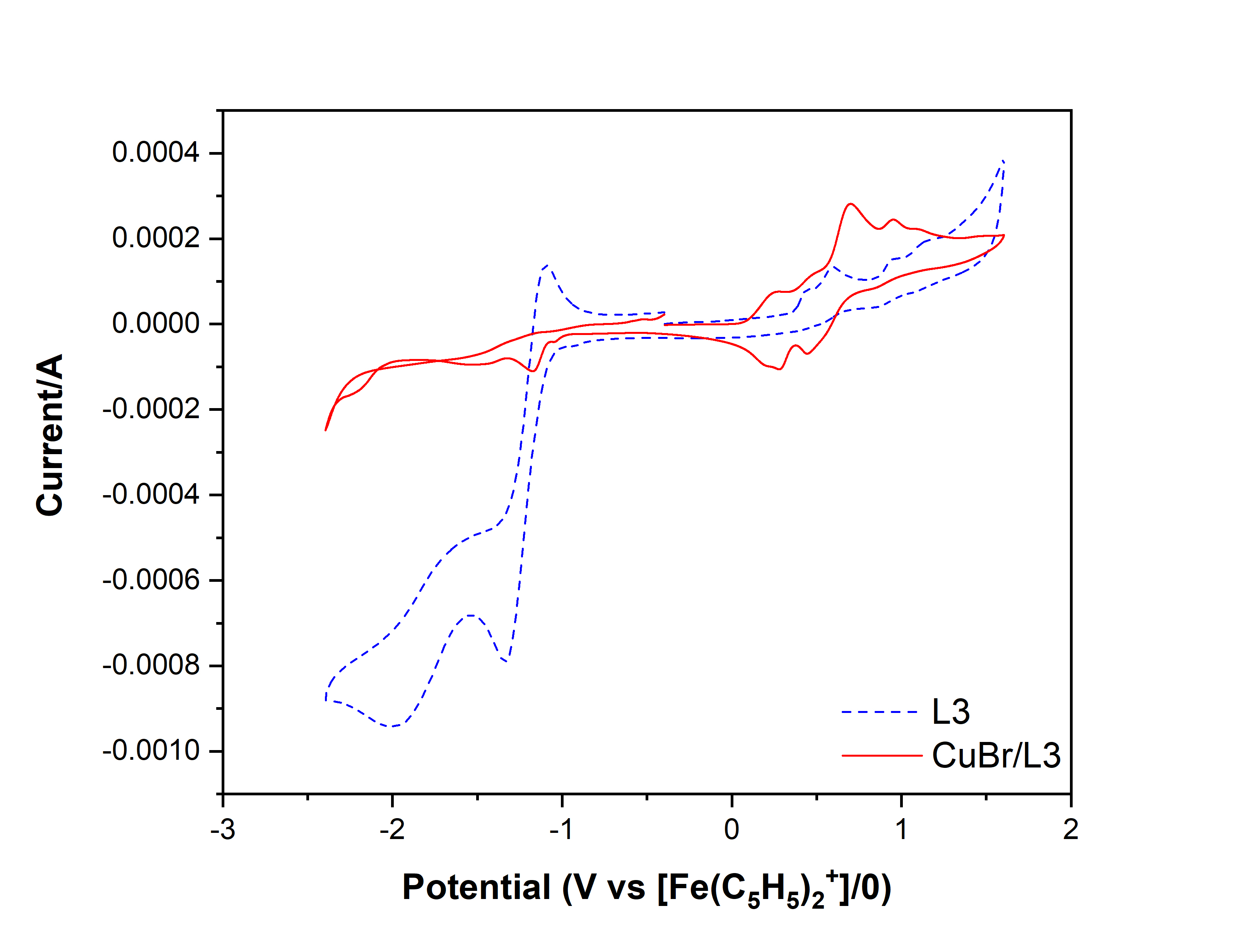

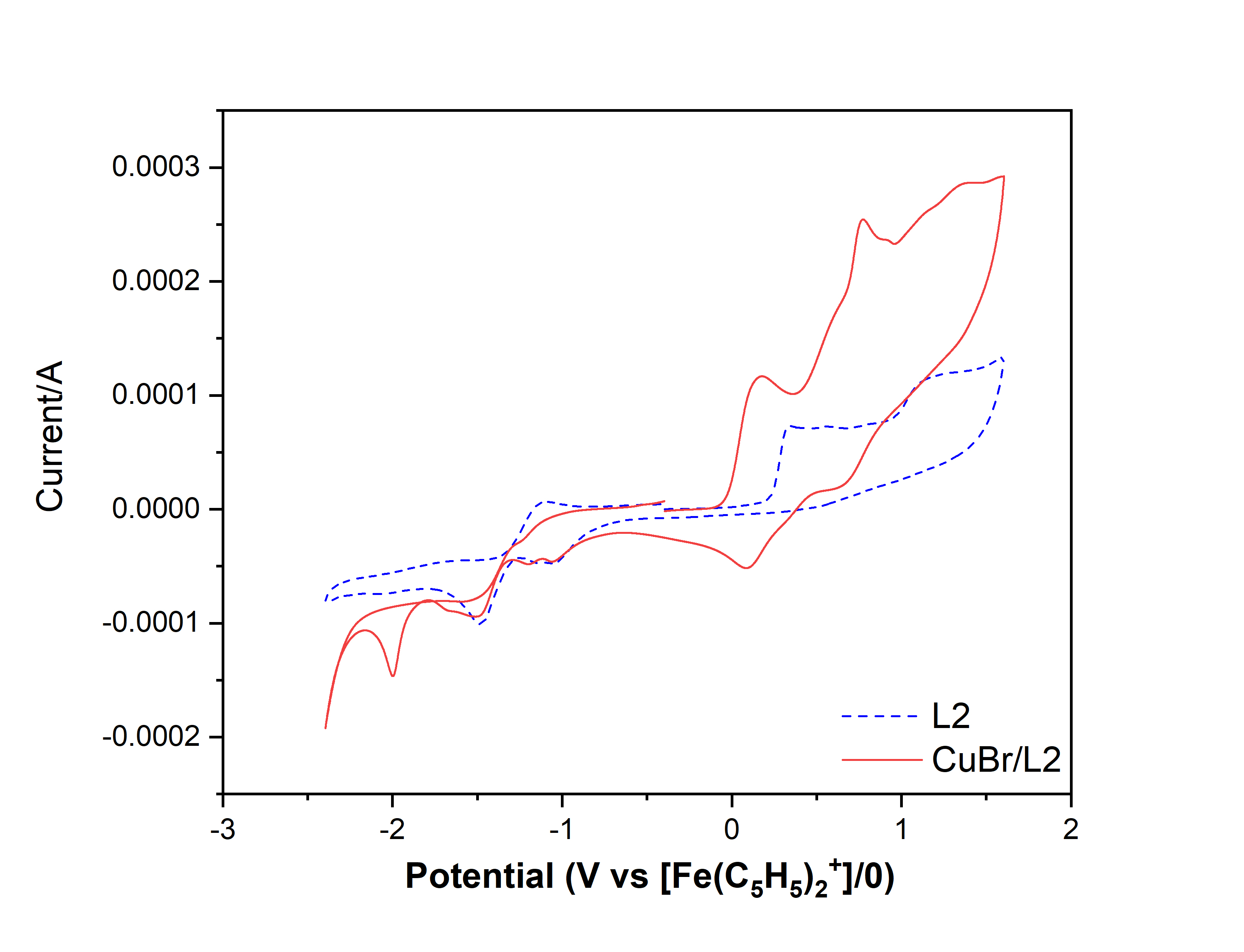

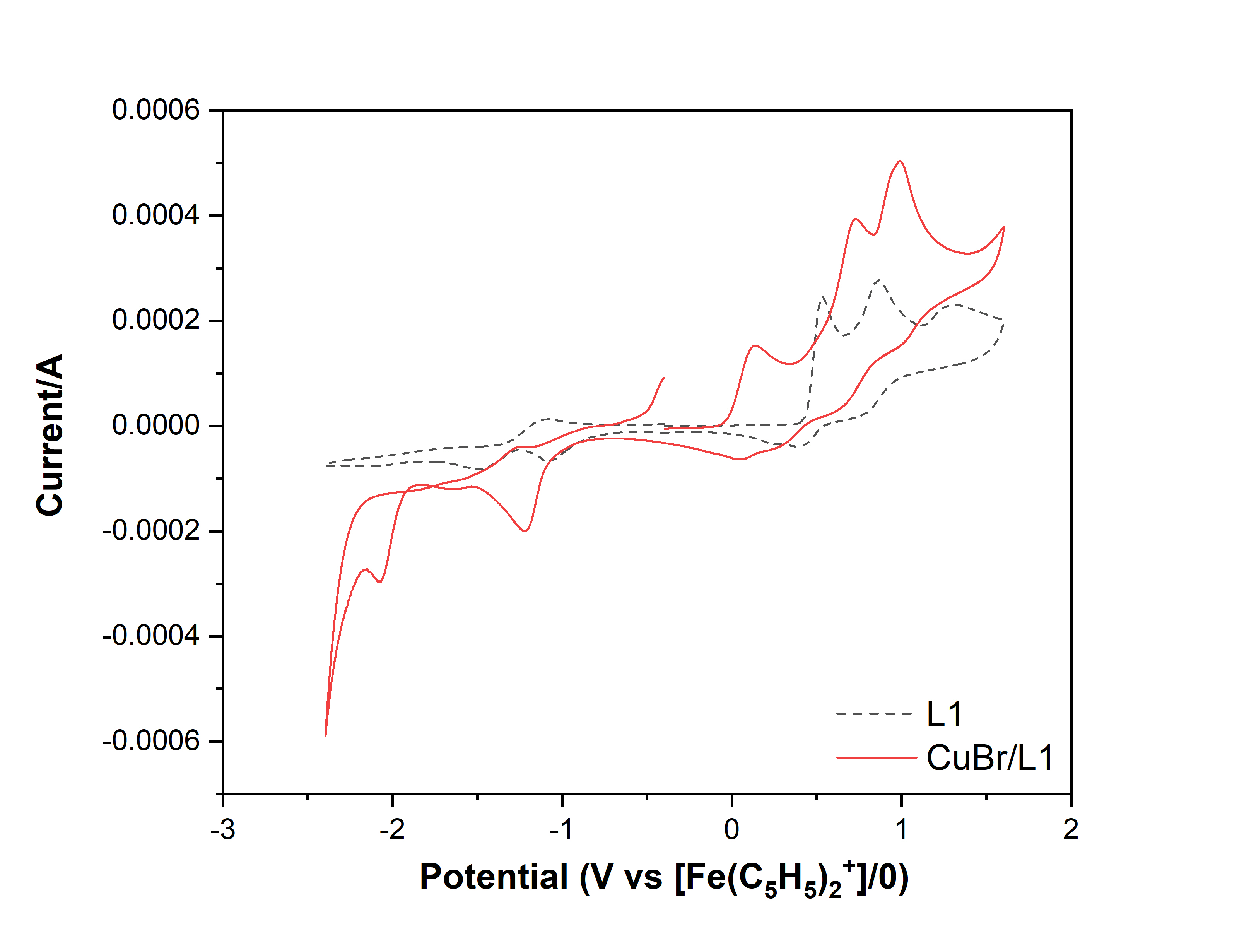


(a)

(b)

(c)

**Figure S11.** Overlaid CV of CuBr/**Ln** and **Ln** where **Ln** = **L1** (a), **L2** (b), and **L3** (c)


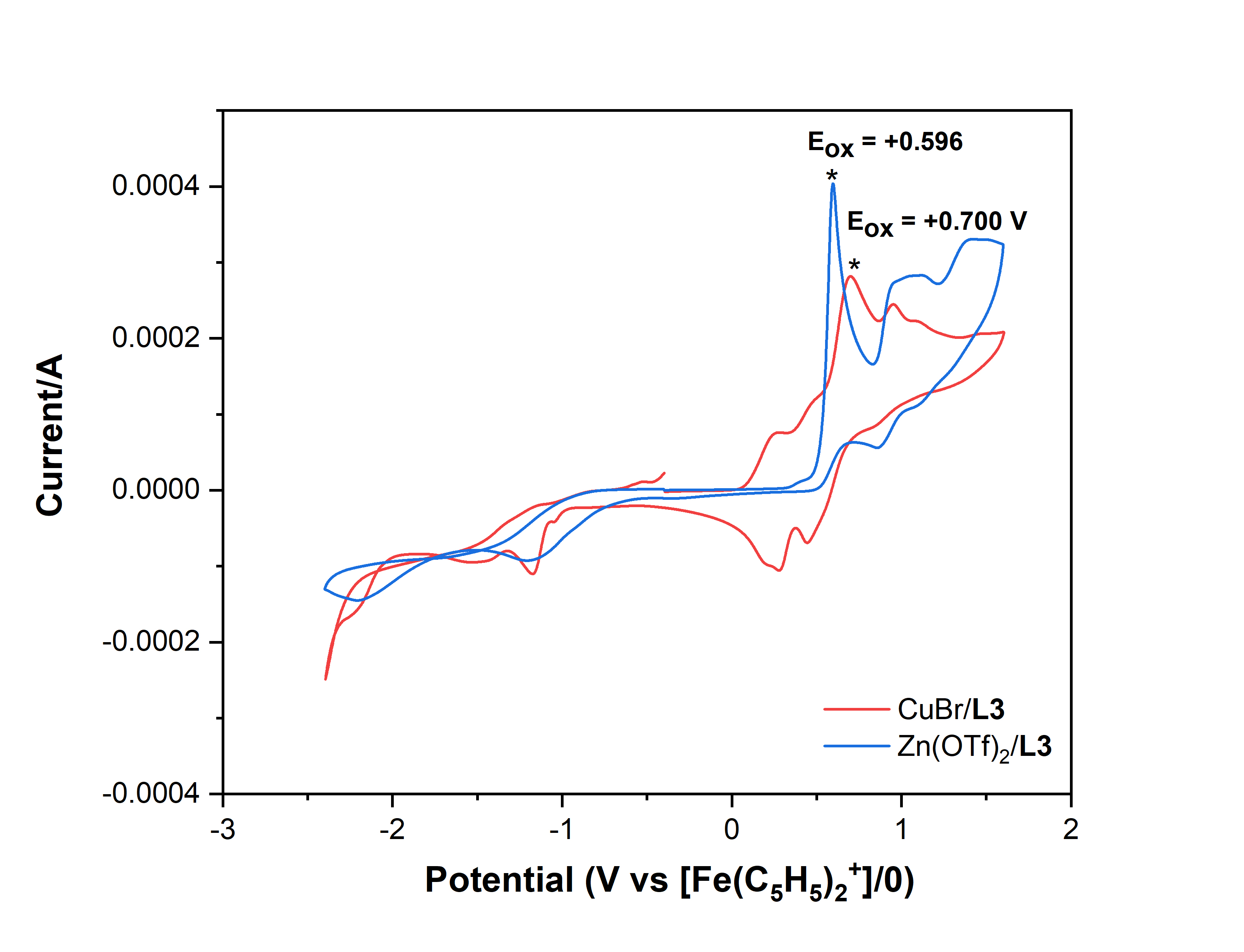

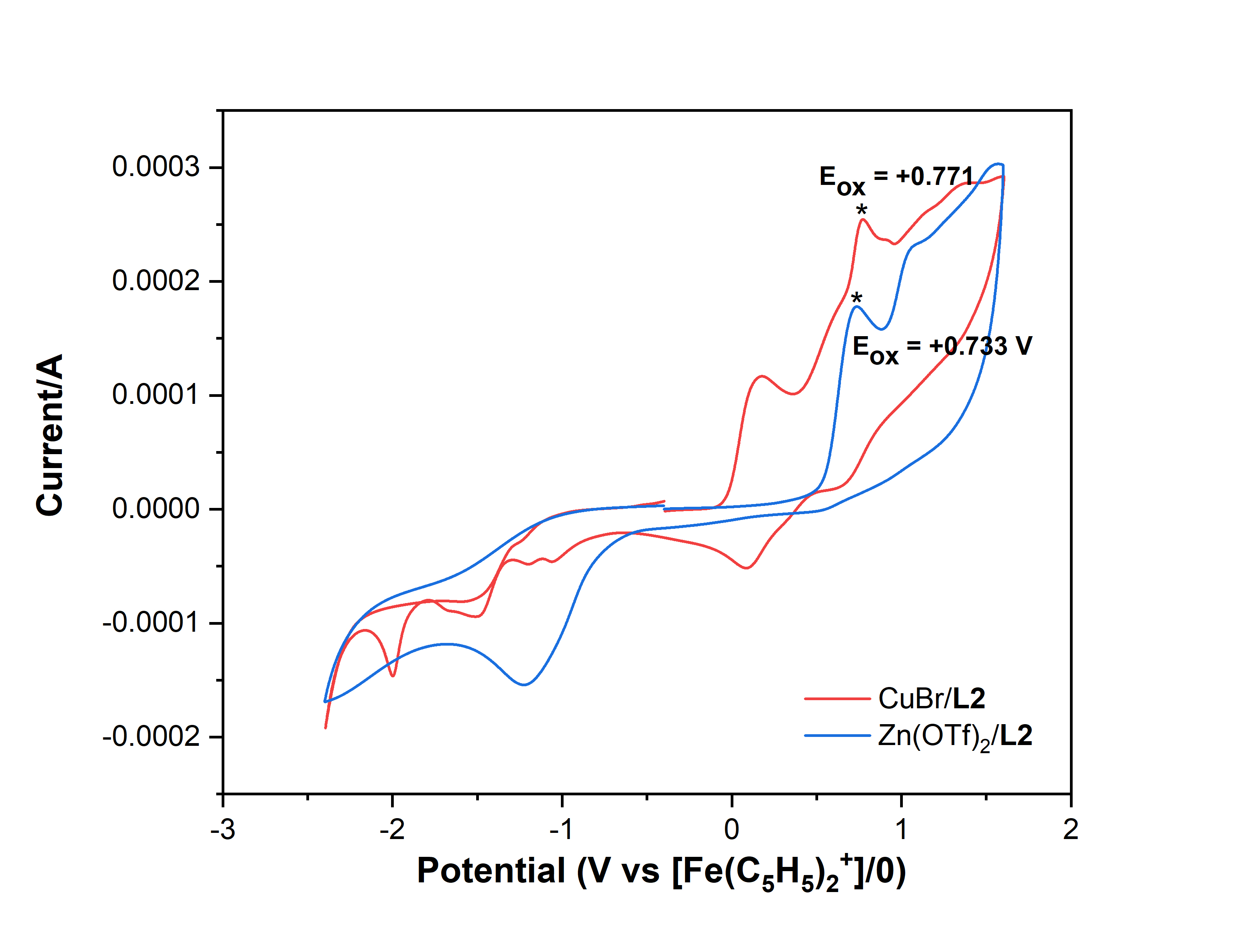

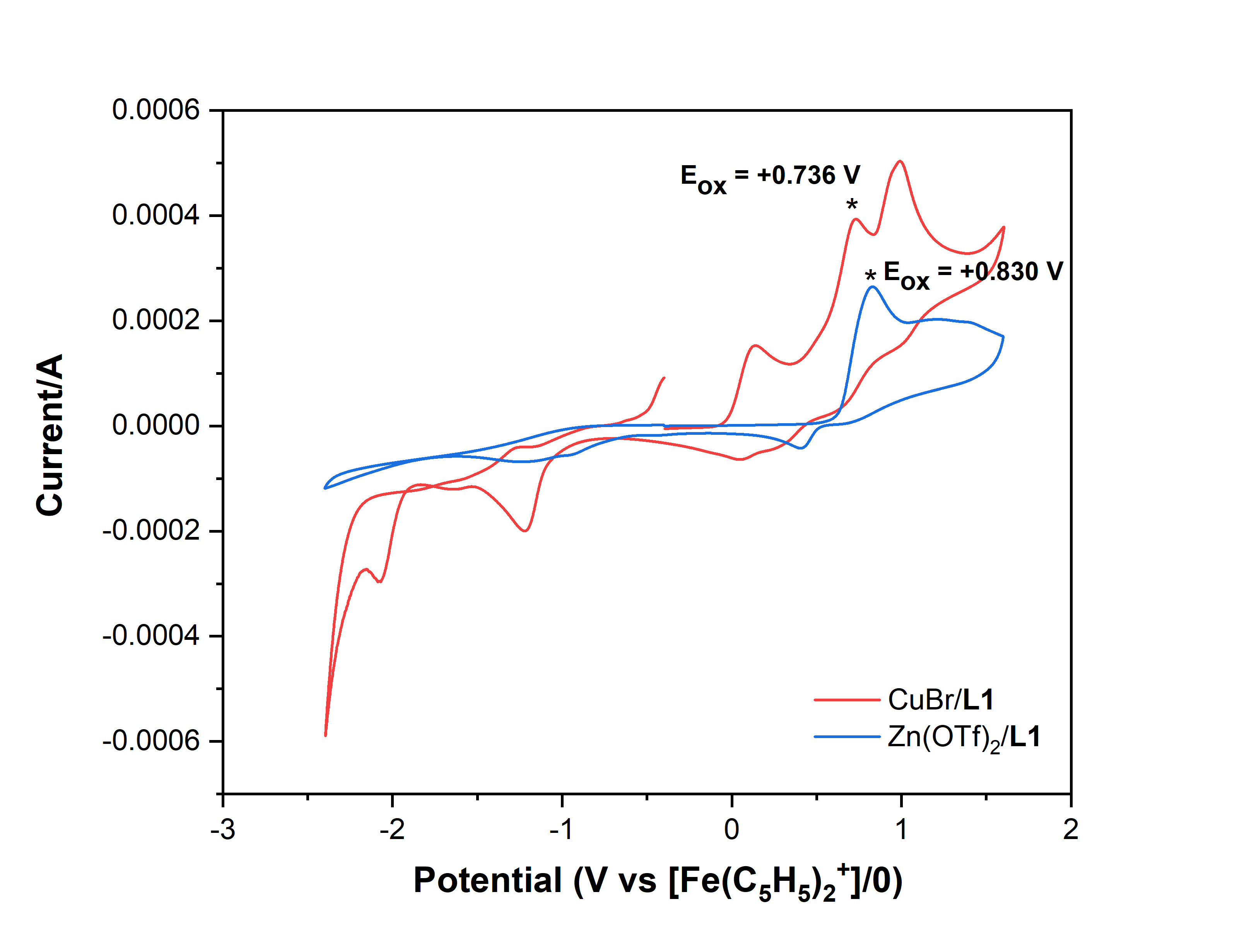


(a)

(b)

(c)

**Figure S12.** Overlaid CV data of CuBr/**Ln** and Zn(OTf)_2_/**Ln** where **Ln** = **L1** (a), **L2** (b), and **L3** (c)

__

**Figure S13.** Square root scan rate *vs*. current of CuBr/**Ln** where **Ln** = **L1** (a), **L2** (b), **L3** (c)


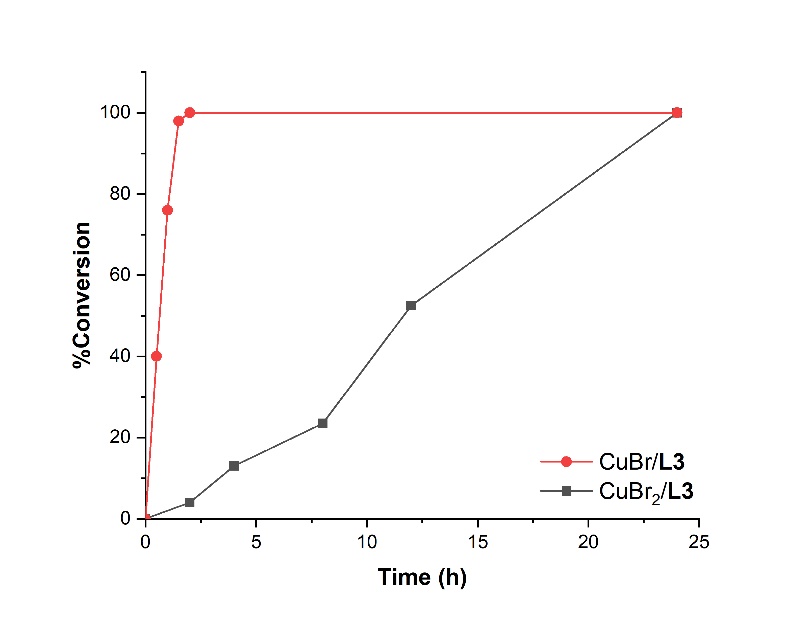


**Figure S14.** Oxidation profile of benzyl alcohol conversion to benzaldehyde by CuBr/**L3** and CuBr_2_/**L3**

**UV-Vis spectrophotometry titrations**

**
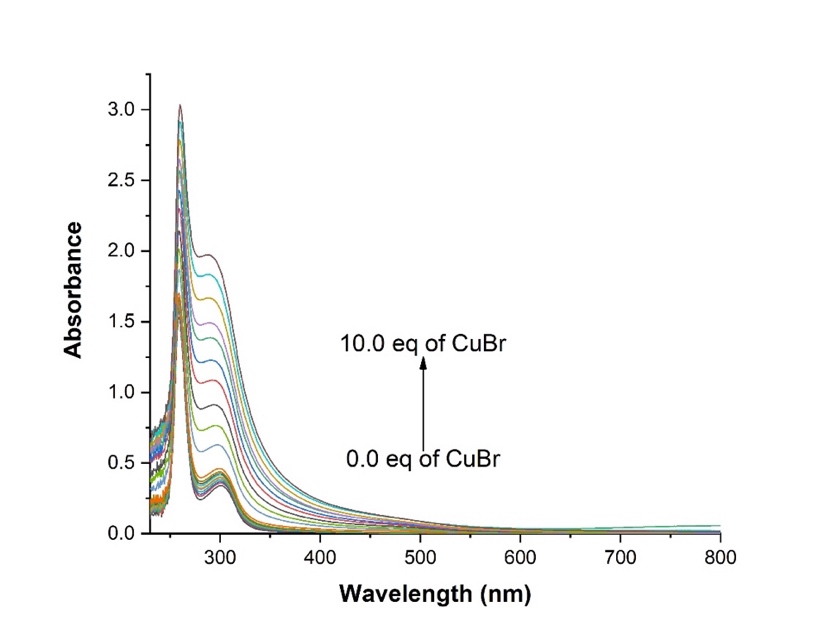
**

**Figure S15.** Absorbance spectra of CuBr at 0.0–10.0 equivalents in 1.0 x 10^-4^ M **L1** (aq.)


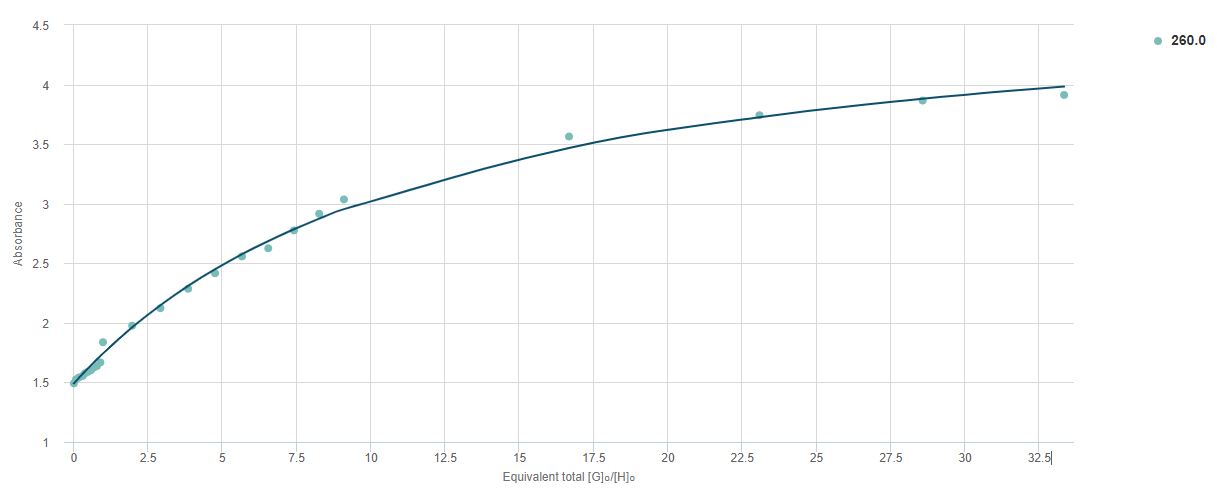

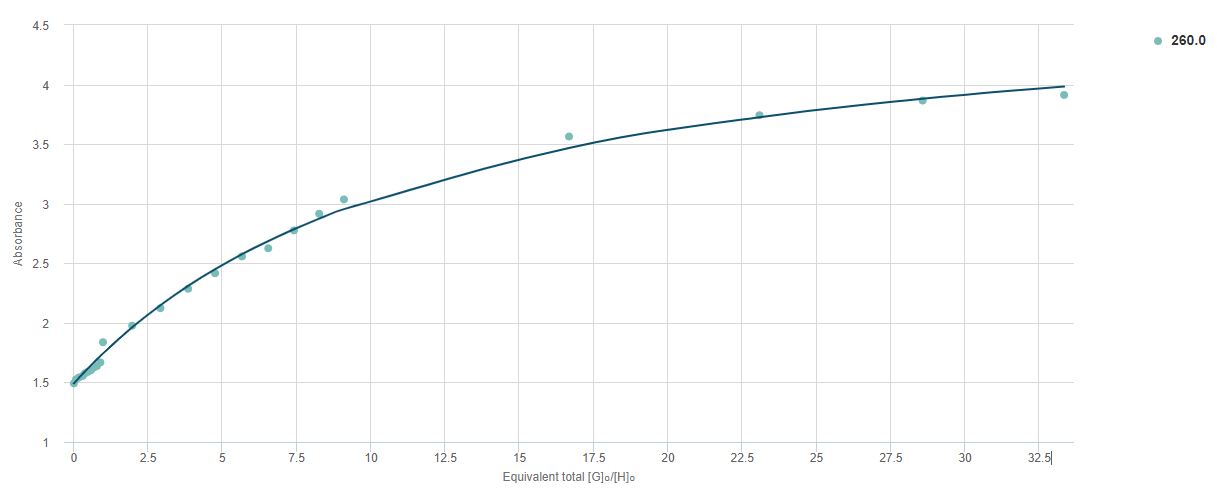


**Figure S16.** Binding isotherms from UV-Vis titrations of **L1** with CuBr. The solid symbols represent experimental data at wavelength of 260 nm and solid lines represent the fitted binding isotherm. The graph was generated from the online program “BindFit” using UV 1:1, the Nelder-Mead method.^7^

**Table S2.** Summary of BindFit analysis from UV-Vis titrations between **L1** and CuBr

| **Stoichiometry** |  | **K** | **K error**  **(%)** |  |  | **Covariance** |
| --- | --- | --- | --- | --- | --- | --- |
| 1:1 |  | 899.92 | 4.0972 |  |  | 3.1914e-3 |
| **Stoichiometry** | **Mode** | **K_11_** | **K_12_** | **K_11_ error(%)** | **K_12_ error(%)** | **Covariance** |
| 1:2 | Full | 539.85 | 303.73 | 3.4474 | 17.5155 | 1.8786e-3 |
|  | Non-Cooperative | 507.17 |  | 3.2191 |  | 1.8902e-3 |
|  | Additive | 486.97 | -53.96 | 3.4938 | -7.8090 | 1.9410e-3 |
|  | Statistical | 1982.45 |  | 4.1783 |  | 2.9815e-3 |
| **Stoichiometry** | **Mode** | **K_11_** | **K_12_** | **K_11_ error(%)** | **K_12_ error(%)** | **Covariance** |
| 2:1 | Full | 416.01 | -1914.52 | 5.8667 | -3.3698 | 1.8762e-3 |
|  | Non-Cooperative | 1843.60 |  | 8.3476 |  | 2.5508e-3 |
|  | Additive | 1643.35 | -1223.60 | 12.6660 | -26.8204 | 2.3653e-3 |
|  | Statistical | 826.25 |  | 3.9026 |  | 3.2947e-3 |

**Result for (L1:CuBr) stoichiometry 1:1 ratio model:**

http://app.supramolecular.org/bindfit/view/9adde80d-0685-4b8c-afa3-73c3fa8fcb35

**
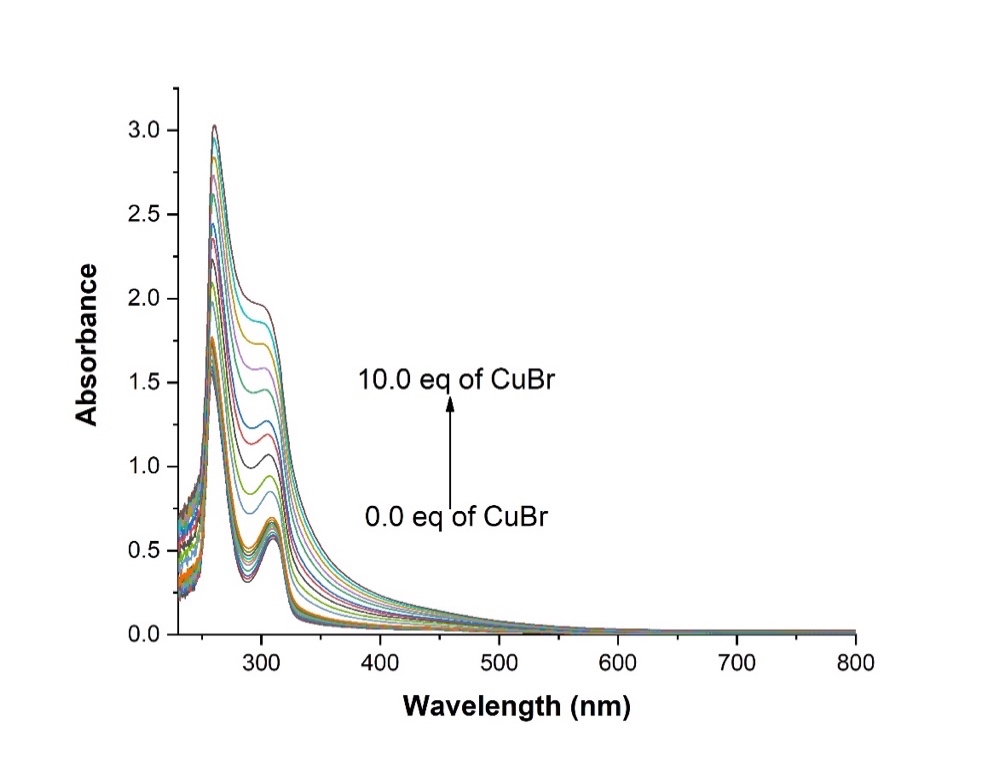
**

**Figure S17.** Absorbance spectra of CuBr at 0.0–10.0 equivalents in 1.0 x 10^-4^ M **L2** (aq.)


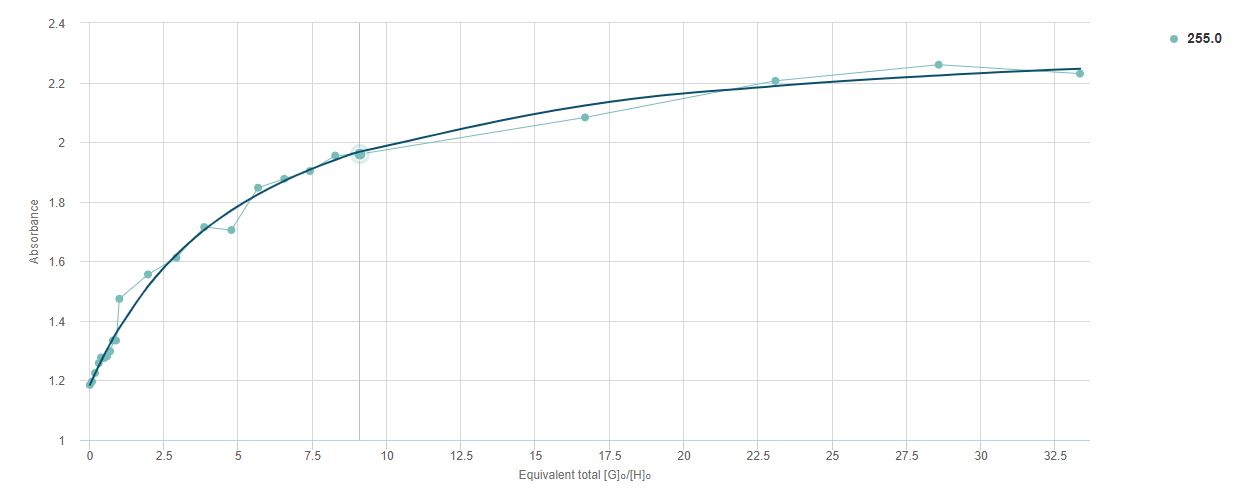

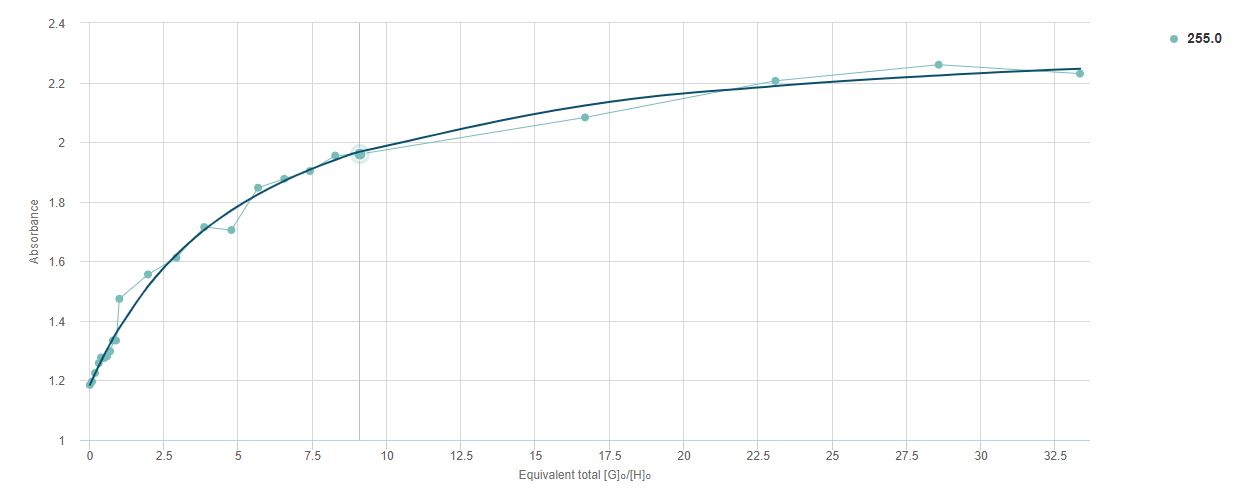


**Figure S18.** Binding isotherms from UV-Vis titrations of **L2** with CuBr. The solid symbols represent experimental data at wavelength of 255 nm and solid lines represent the fitted binding isotherm. The graph was generated from the online program “BindFit” using UV 2:1, the Nelder-Mead method.^7^

**Table S3.** Summary of BindFit analysis from UV-Vis titrations between **L2** and CuBr

| **Stoichiometry** |  | **K** | **K error**  **(%)** |  |  | **Covariance** |
| --- | --- | --- | --- | --- | --- | --- |
| 1:1 |  | 2217.56 | 7.5071 |  |  | 7.5329e-3 |
| **Stoichiometry** | **Mode** | **K_11_** | **K_12_** | **K_11_ error(%)** | **K_12_ error(%)** | **Covariance** |
| 1:2 | Full |  |  |  |  |  |
|  | Non-Cooperative | 3931.99 |  | 8.5868 |  | 7.9446e-3 |
|  | Additive | 2682.90 | 31.98 | 9.8731 | 52.4564 | 7.2527e-3 |
|  | Statistical | 5277.05 |  | 8.7400 |  | 8.1800e-3 |
| **Stoichiometry** | **Mode** | **K_11_** | **K_12_** | **K_11_ error(%)** | **K_12_ error(%)** | **Covariance** |
| 2:1 | Full |  |  |  |  |  |
|  | Non-Cooperative | 2009.46 |  | 7.1231 |  | 7.3474e-3 |
|  | Additive | 548.53 | 8343.84 | 41.2815 | 43.4962 | 7.2121e-3 |
|  | Statistical | 1870.05 |  | 6.4709 |  | 7.3537e-3 |

**Result for (L2:CuBr) stoichiometry statistical model in 1:2 ratio:**

http://app.supramolecular.org/bindfit/view/7b6d17c4-df89-4091-8f7a-50c4c938dd58

**
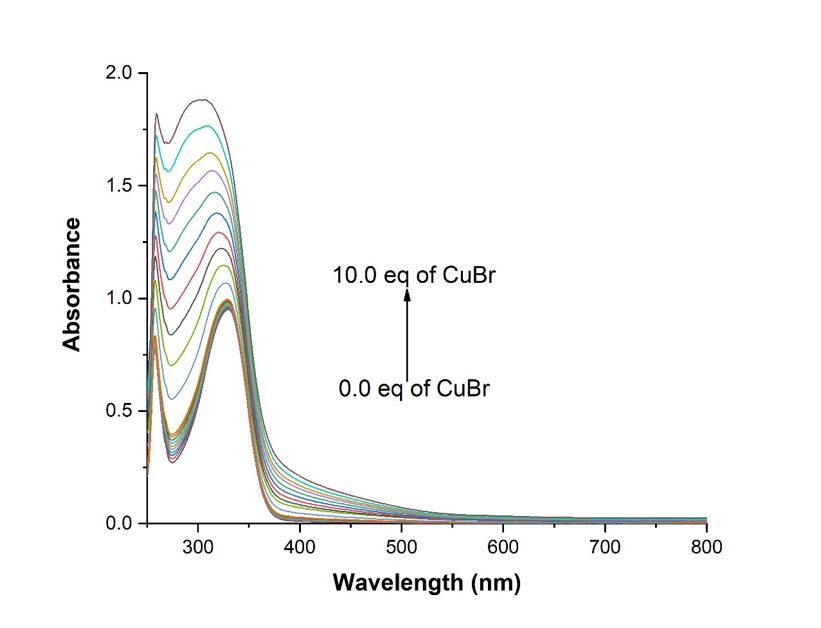
**

**Figure S19.** Absorbance spectra of CuBr at 0.0–10.0 equivalents in 1.0 x 10^-4^ M **L3** (aq.)


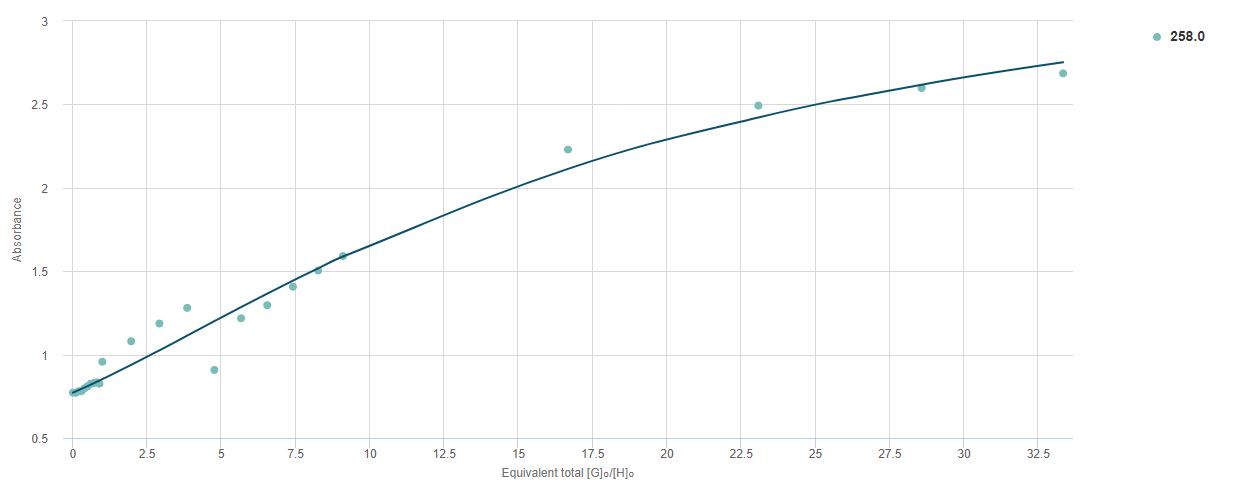

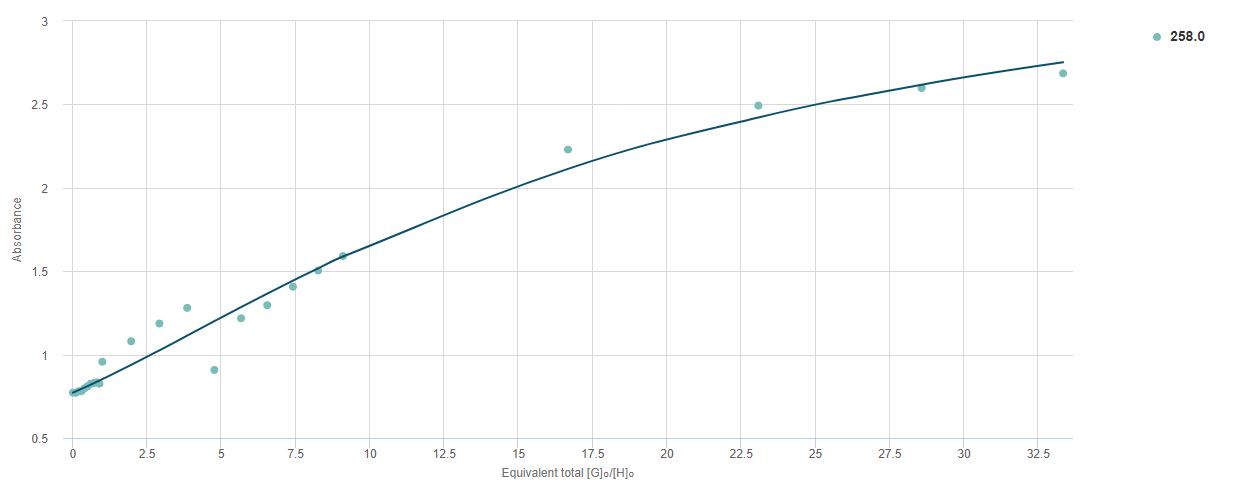


**Figure S20.** Binding isotherms from UV-Vis titrations of **L3** with CuBr. The solid symbols represent experimental data at wavelength of 258 nm and solid lines represent the fitted binding isotherm. The graph was generated from the online program “BindFit” using UV 2:1, the Nelder-Mead method.^7^

**Table S4.** Summary of BindFit analysis from UV-Vis titrations between **L3** and CuBr

| **Stoichiometry** |  | **K** | **K error**  **(%)** |  |  | **Covariance** |
| --- | --- | --- | --- | --- | --- | --- |
| 1:1 |  | 238.98 | 8.5213 |  |  | 2.5578e-2 |
| **Stoichiometry** | **Mode** | **K_11_** | **K_12_** | **K_11_ error(%)** | **K_12_ error(%)** | **Covariance** |
| 1:2 | Full | -195.38 | -351.77 | -9.9747 | -15.1702 | 2.0046e-2 |
|  | Non-Cooperative | 1592.77 |  | 9.3234 |  | 2.3750e-2 |
|  | Additive | 571.66 | 719.71 | 40.4320 | 46.6436 | 2.2460e-2 |
|  | Statistical | 496.16 |  | 8.6760 |  | 2.5535e-2 |
| **Stoichiometry** | **Mode** | **K_11_** | **K_12_** | **K_11_ error(%)** | **K_12_ error(%)** | **Covariance** |
| 2:1 | Full | 1140.51 | -4978.31 | 15.4087 | -3.9917 | 1.8825e-2 |
|  | Non-Cooperative | 735.62 |  | 25.8192 |  | 2.3980e-2 |
|  | Additive | 835.20 | -1711.81 | 25.2889 | -32.5196 | 2.3046e-2 |
|  | Statistical | 232.03 |  | 8.3516 |  | 2.5599e-2 |

**Result for (L3:CuBr) stoichiometry statistical model in 1:2 ratio:**

http://app.supramolecular.org/bindfit/view/1347330e-e589-4f7a-9aaa-7cc1d634beeb

"BindFit v0.5 | Supramolecular," can be found at http://supramolecular.org.

**Table S5.** Examples of GC-MS data from aerobic oxidation of various alcohols by CuBr/**L3**/NMI/TEMPO in CH_3_CN


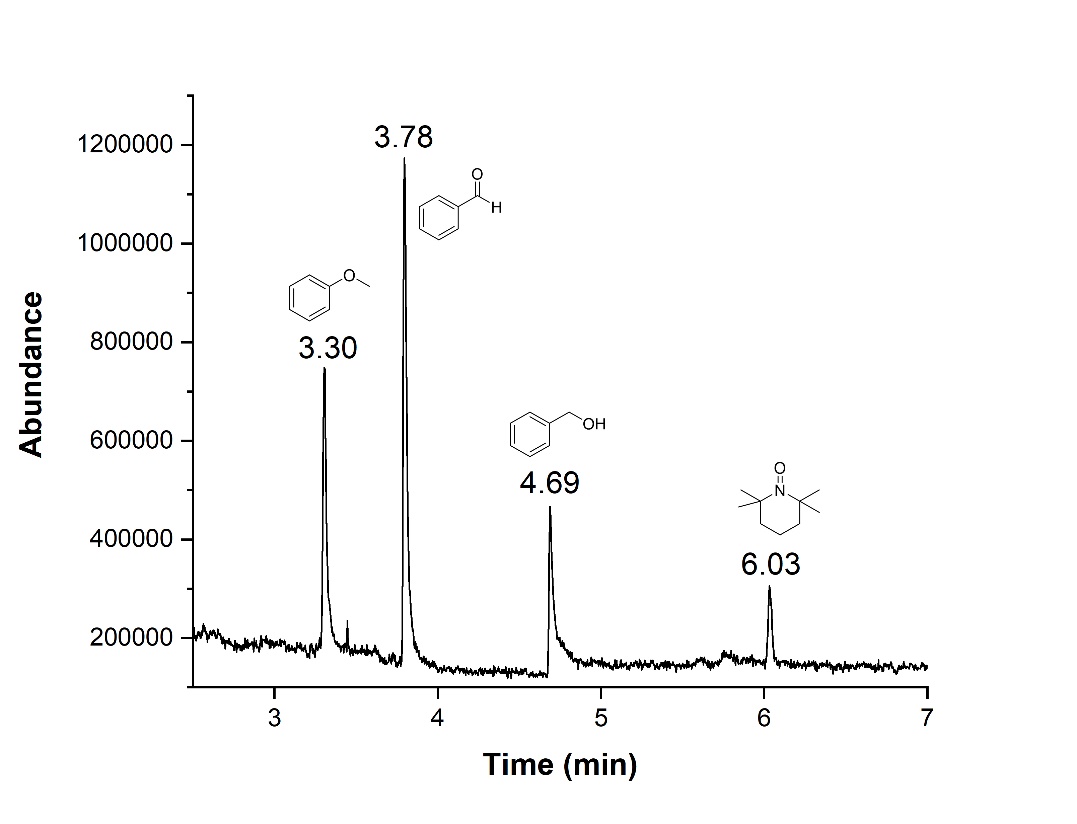


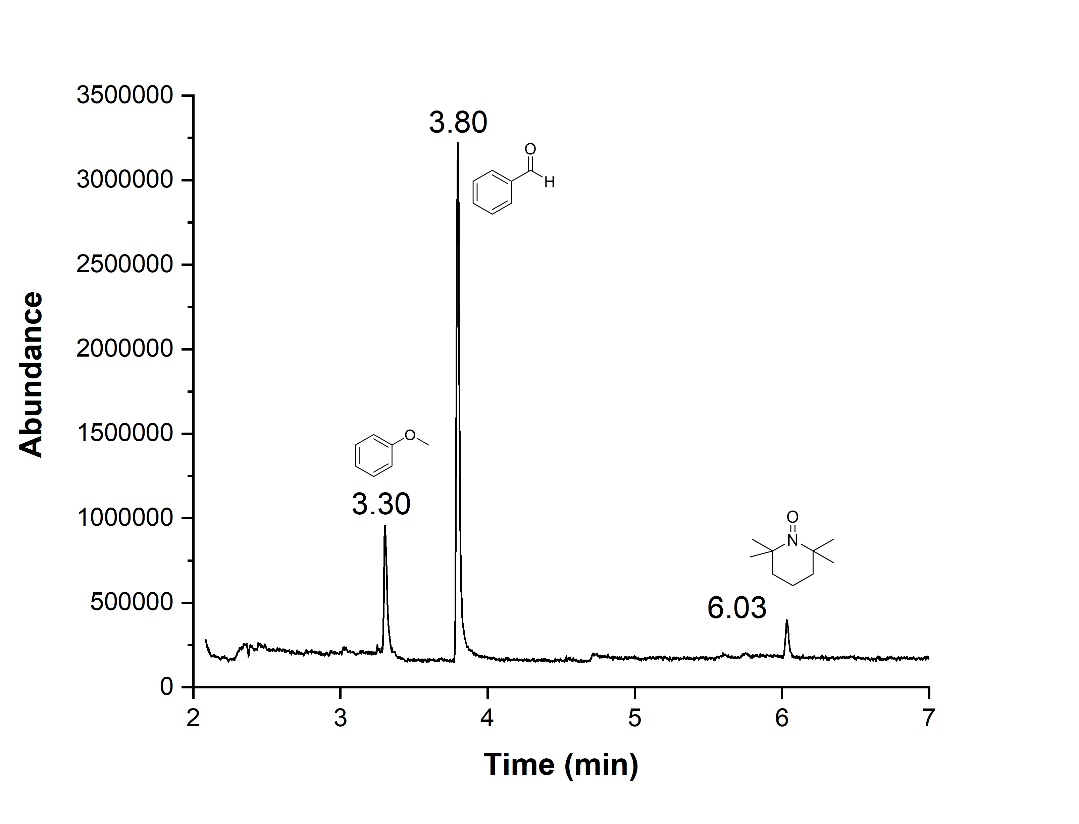
**Catalytic conditions:** Benzyl alcohol (1.0 mmol), 2.5 mol% CuBr / 1.25 mol% **L3** / 5 mol% TEMPO / 10 mol% NMI, anisole (0.10 mmol), ambient temperature, time = 30 min

**Catalytic conditions:** Benzyl alcohol (1.0 mmol), 2.5 mol% CuBr / 1.25 mol% **L3** / 5 mol% TEMPO / 10 mol% NMI, anisole (0.10 mmol), ambient temperature, time = 90 min


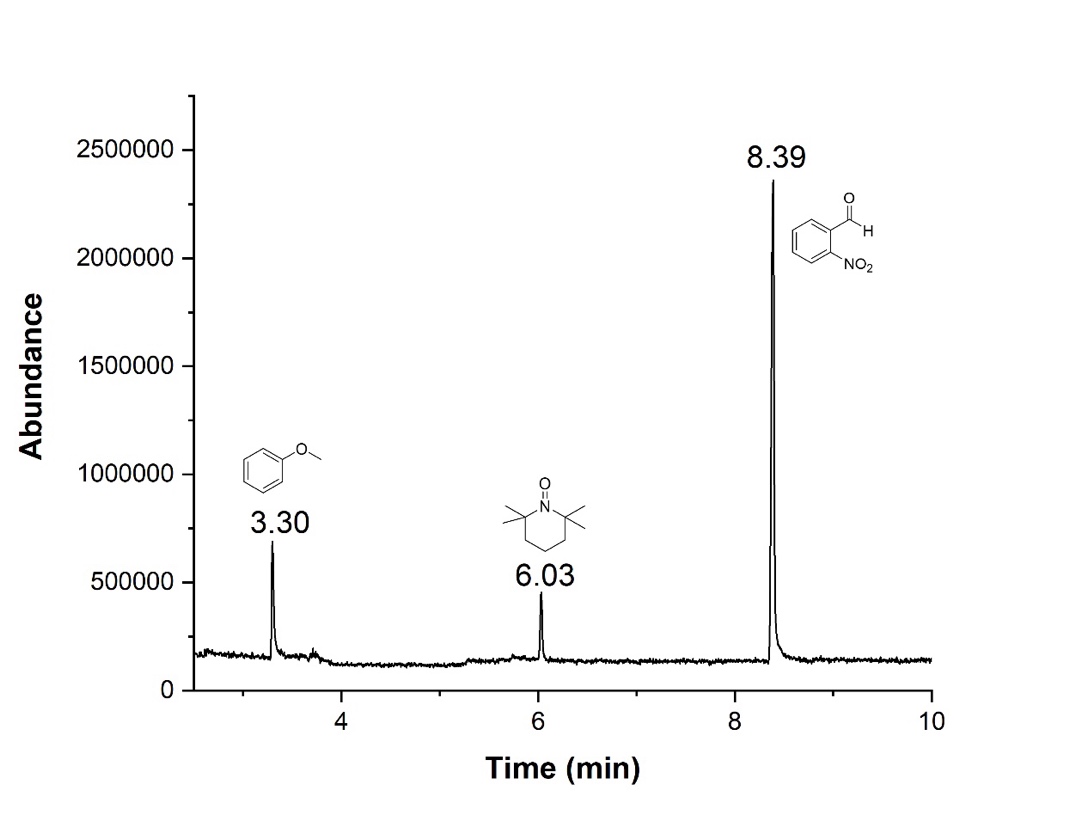


**Catalytic conditions:** 2-nitrobenzyl alcohol (1.0 mmol), 2.5 mol% CuBr / 1.25 mol% **L3** / 5 mol% TEMPO / 10 mol% NMI, anisole (0.10 mmol), ambient temperature, time = 2 h

**
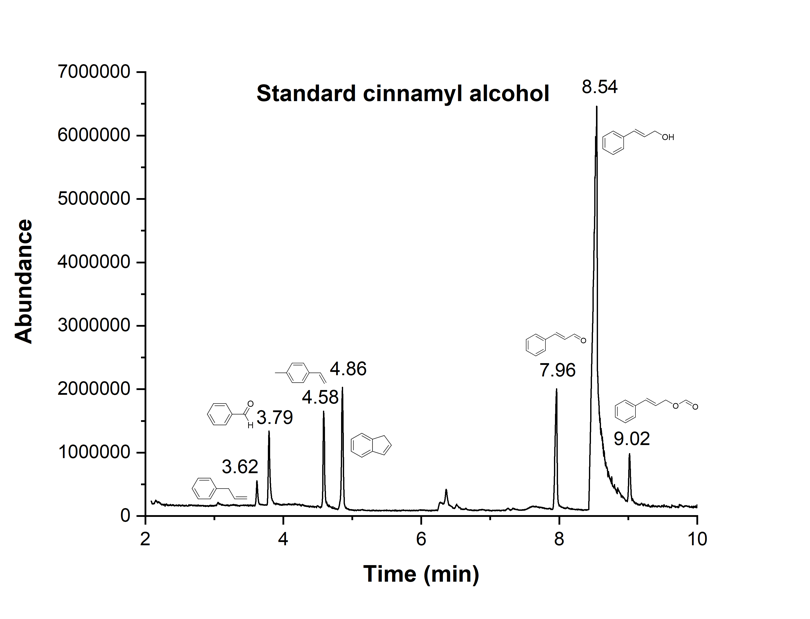
**

**
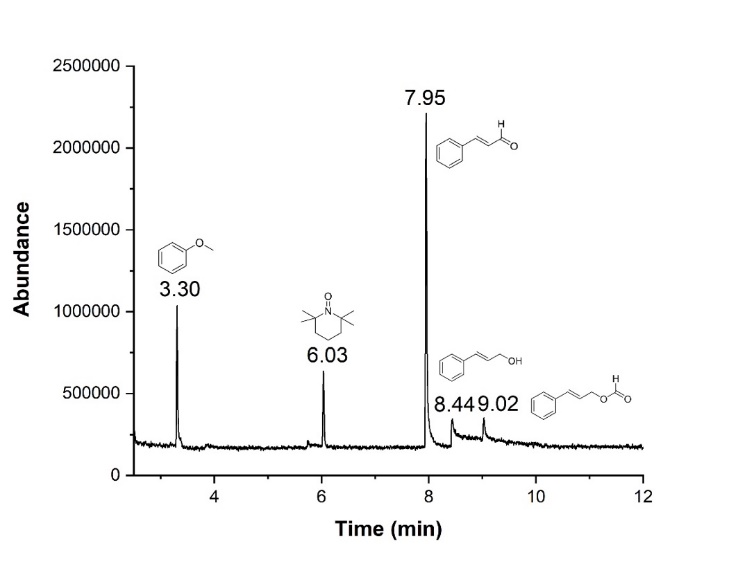
**

**Catalytic conditions:** cinnamyl alcohol (1.0 mmol), 2.5 mol% CuBr / 1.25 mol% **L3** / 5 mol% TEMPO / 10 mol% NMI, anisole (0.10 mmol), ambient temperature, time = 2 h. The GC-MS spectrum of the cinnamyl alcohol substrate is shown on the right.

**
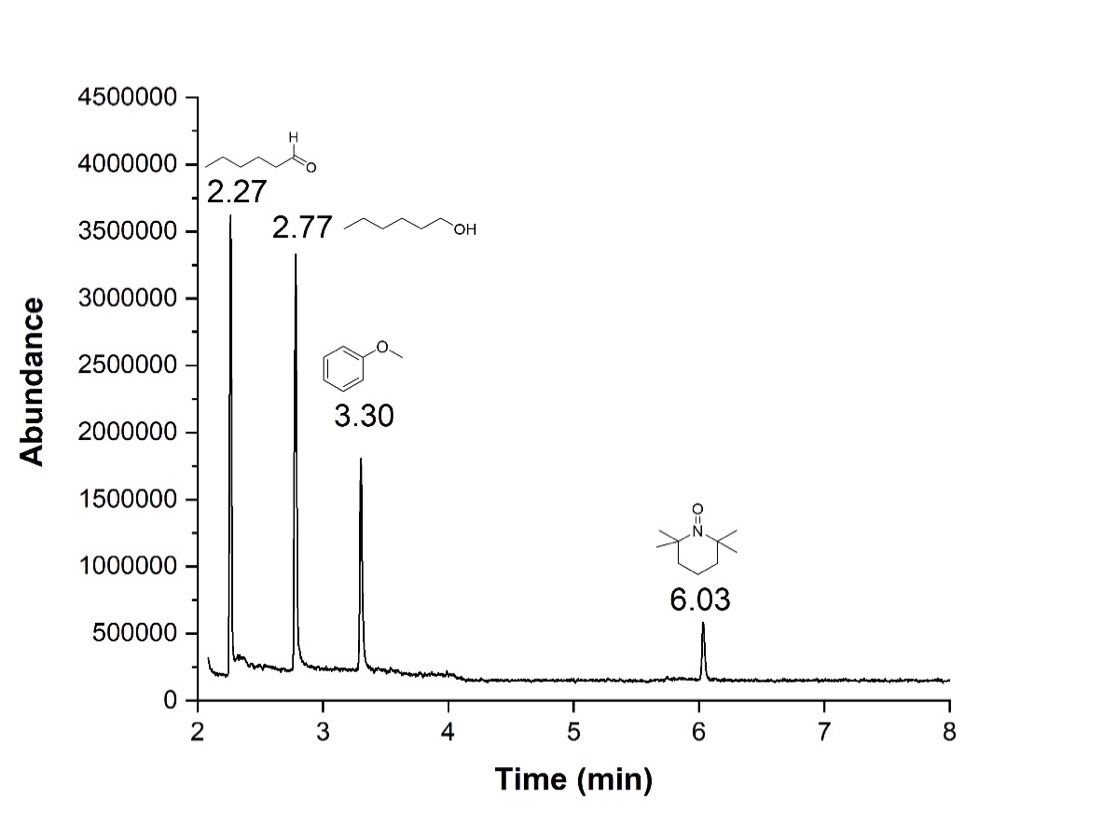
**

**Catalytic conditions:** 1-hexanol (1.0 mmol), 2.5 mol% CuBr / 1.25 mol% **L3** / 5 mol% TEMPO / 10 mol% NMI, anisole (0.10 mmol), temperature = 60 ^o^C, time = 24 h

**
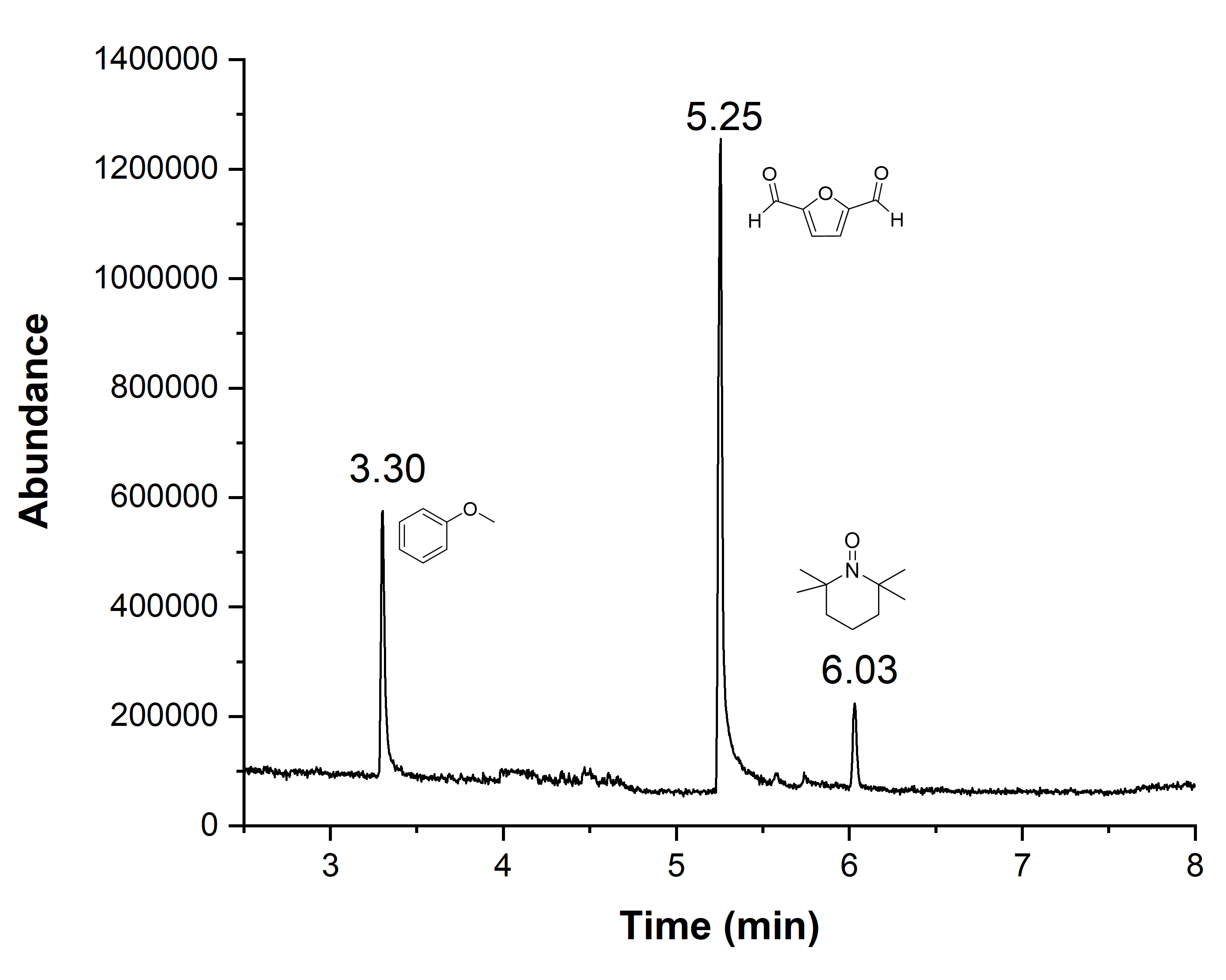
**

**Catalytic conditions:** furfuryl alcohol (1.0 mmol), 2.5 mol% CuBr / 1.25 mol% **L3** / 5 mol% TEMPO / 10 mol% NMI, anisole (0.10 mmol), ambient temperature, time = 2 h


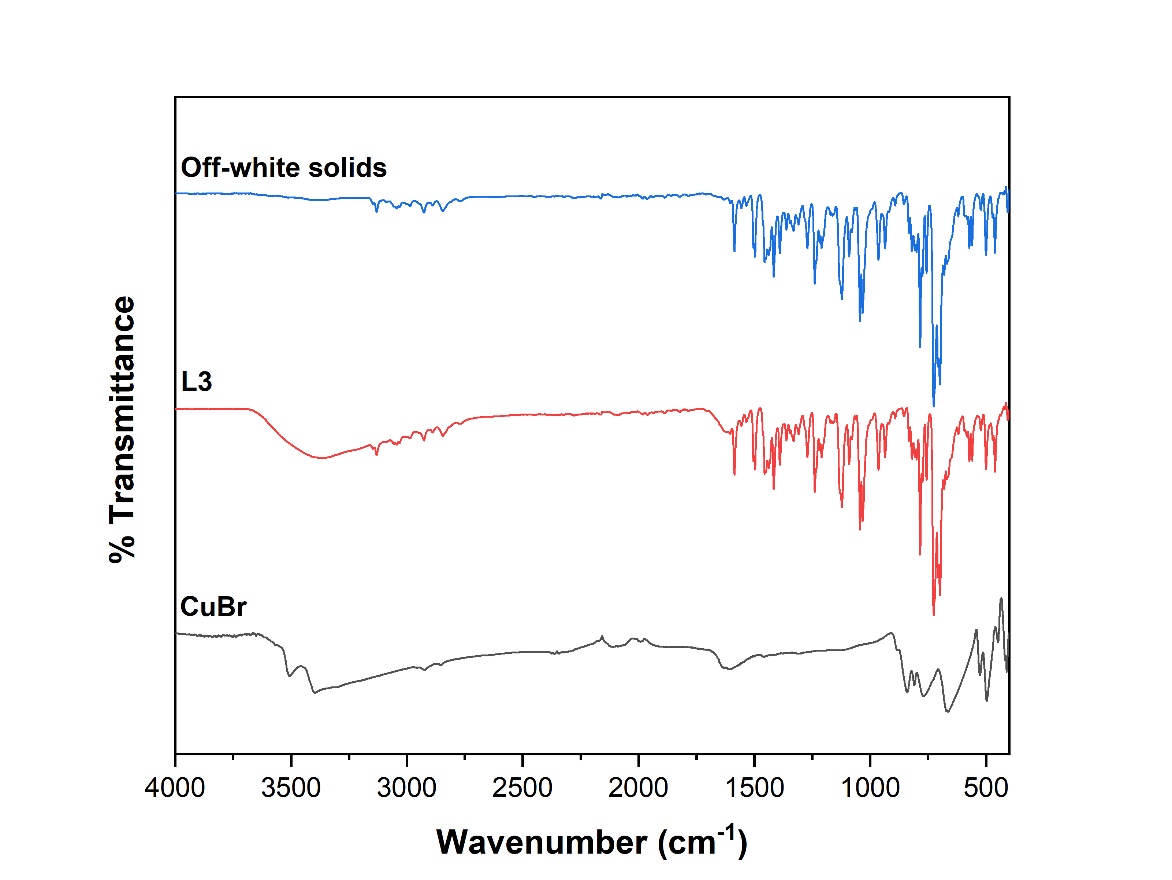


**Figure S21.** FT-IR spectra of CuBr, free ligand **L3,** and off-white solids


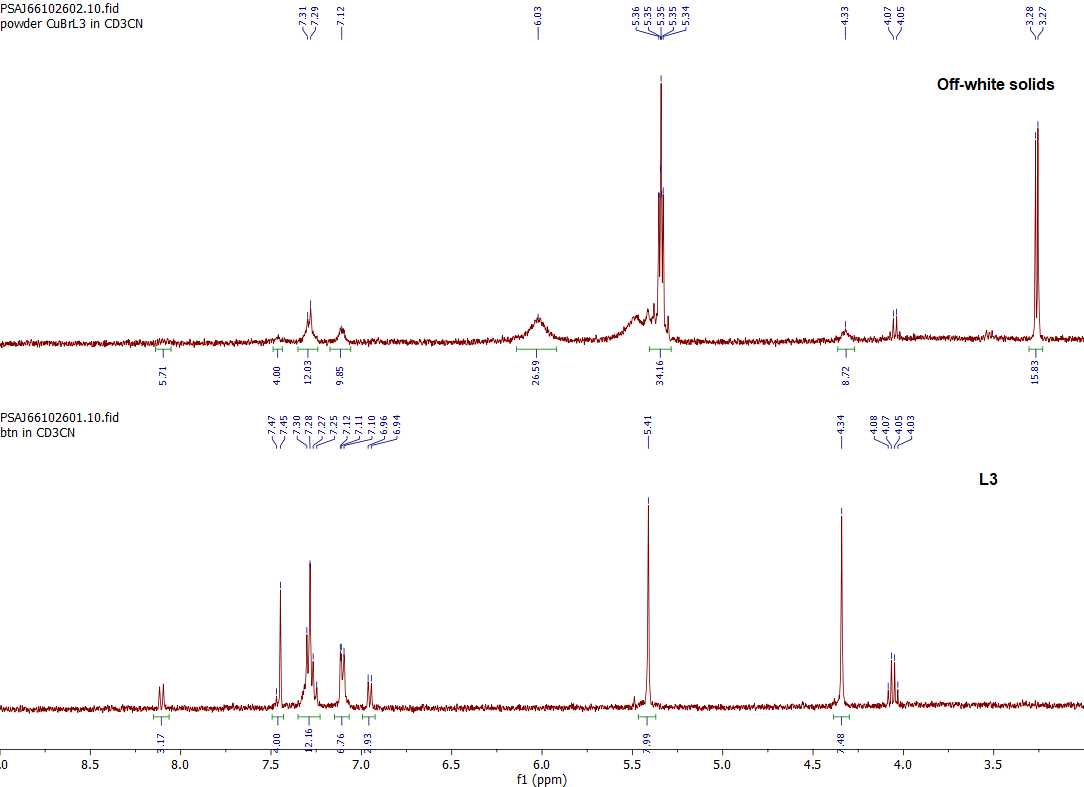


**Figure S22.** ^1^H NMR spectra of off-white solids and **L3**


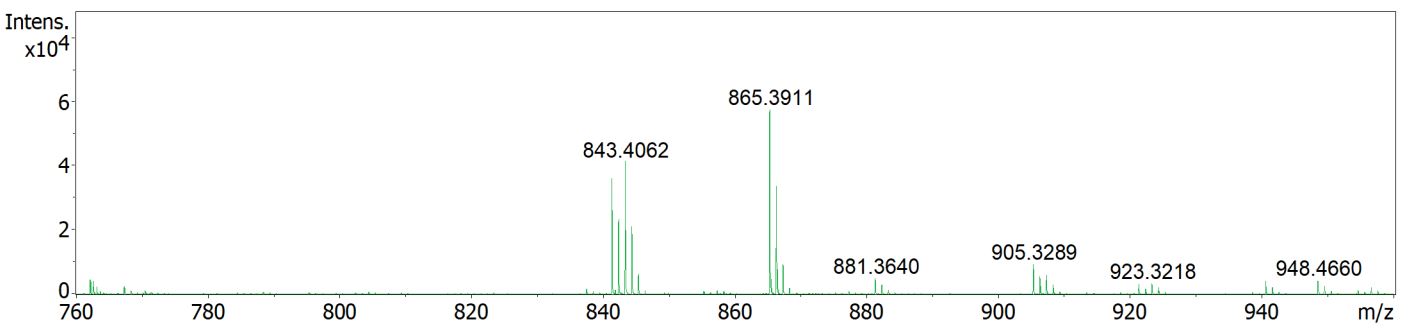


[**L3**+H^+^]

[**L3**+Na^+^]

[**L3**+Cu^+^]

**Figure S23.** ESI-MS spectra of off-white solids

**Theoretical study on Cu(I)Br complexes of L1–L3**

**Computational details**

CREST^1^ program was used to explore the lowest-energy conformations of each intermediate. All geometry optimizations and frequency calculations were performed using Gaussian16.^2^ The input geometries produced by CREST were optimized in the gas-phase using ωB97XD^3^ functional and def2-SVP^4^ basis set. Frequency calculations at the same level were performed to ensure that the optimized geometries corresponded to either energy minima with no imaginary frequency or transition states with one imaginary frequency. To take into account of the solvent effect, the single-point energy calculations were carried out with the SMD^5^ continuum solvation model with the solvent parameters of dimethylsulfoxide (ε = 46.826) using ωB97XD functional and def2-TZVPP basis set. Finally, the quasi-harmonic approximation implemented in the GoodVibes^6^ program was used to correct the free energies.

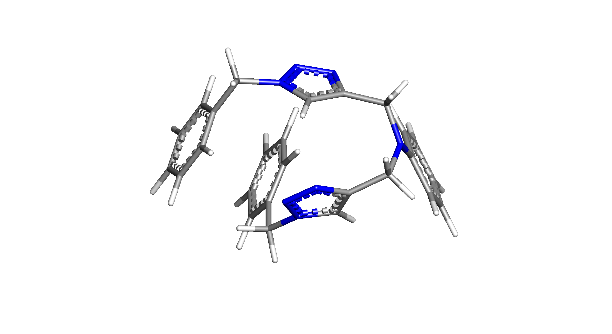
 **
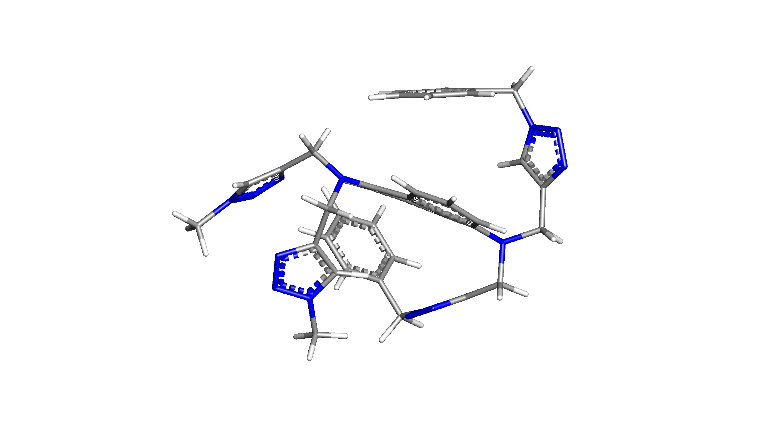
**

L1 L3’

**Figure S24.** Optimized geometries of **L1** and **L3’**

**References**

1. Pracht, P.; Bohle, F.; Grimme, S., Automated exploration of the low-energy chemical space with fast quantum chemical methods. *Physical Chemistry Chemical Physics* **2020,** *22* (14), 7169-7192.

2. Frisch, M. J.; Trucks, G. W.; Schlegel, H. B.; Scuseria, G. E.; Robb, M. A.; Cheeseman, J. R.; Scalmani, G.; Barone, V.; Petersson, G. A.; Nakatsuji, H.; Li, X.; Caricato, M.; Marenich, A. V.; Bloino, J.; Janesko, B. G.; Gomperts, R.; Mennucci, B.; Hratchian, H. P.; Ortiz, J. V.; Izmaylov, A. F.; Sonnenberg, J. L.; Williams; Ding, F.; Lipparini, F.; Egidi, F.; Goings, J.; Peng, B.; Petrone, A.; Henderson, T.; Ranasinghe, D.; Zakrzewski, V. G.; Gao, J.; Rega, N.; Zheng, G.; Liang, W.; Hada, M.; Ehara, M.; Toyota, K.; Fukuda, R.; Hasegawa, J.; Ishida, M.; Nakajima, T.; Honda, Y.; Kitao, O.; Nakai, H.; Vreven, T.; Throssell, K.; Montgomery Jr., J. A.; Peralta, J. E.; Ogliaro, F.; Bearpark, M. J.; Heyd, J. J.; Brothers, E. N.; Kudin, K. N.; Staroverov, V. N.; Keith, T. A.; Kobayashi, R.; Normand, J.; Raghavachari, K.; Rendell, A. P.; Burant, J. C.; Iyengar, S. S.; Tomasi, J.; Cossi, M.; Millam, J. M.; Klene, M.; Adamo, C.; Cammi, R.; Ochterski, J. W.; Martin, R. L.; Morokuma, K.; Farkas, O.; Foresman, J. B.; Fox, D. J. *Gaussian 16 Rev. C.01*, Wallingford, CT, 2016.

3. Chai, J.-D.; Head-Gordon, M., Long-range corrected hybrid density functionals with damped atom–atom dispersion corrections. *Physical Chemistry Chemical Physics* **2008,** *10* (44), 6615-6620.

4. Weigend, F.; Ahlrichs, R., Balanced basis sets of split valence, triple zeta valence and quadruple zeta valence quality for H to Rn: Design and assessment of accuracy. *Physical Chemistry Chemical Physics* **2005,** *7* (18), 3297-3305.

5. Marenich, A. V.; Cramer, C. J.; Truhlar, D. G., Universal Solvation Model Based on Solute Electron Density and on a Continuum Model of the Solvent Defined by the Bulk Dielectric Constant and Atomic Surface Tensions. *The Journal of Physical Chemistry B* **2009,** *113* (18), 6378-6396.

6. Luchini, G.; Alegre-Requena, J. V.; Funes-Ardoiz, I.; Paton, R. S., GoodVibes: automated thermochemistry for heterogeneous computational chemistry data [version 1; peer review: 2 approved with reservations]. *F1000Research* (2020, 9(Chem Inf Sci)), 291.

7. BindFit v0.5 | Supramolecular, http://app.supramolecular.org/bindfit/, (acessed November 2023).

**Cartesian Coordinates**

**L1**

58

N -0.707834 -1.495373 -2.332568

N 0.578510 -1.597273 -2.409307

N 0.994156 -2.285546 -1.348179

N -0.553359 -1.856228 2.362341

N 0.479606 -1.101298 2.551633

N 0.105031 0.159711 2.339515

N -3.178450 -1.345365 0.121134

C -0.045951 -2.638143 -0.563201

C -1.151132 -2.120410 -1.208752

C -2.619431 -2.236797 -0.877691

C -2.909996 -1.675523 1.512190

C -1.617699 -1.088980 2.008038

C -1.201925 0.225454 2.007776

C 2.412752 -2.550170 -1.152533

C 3.168979 -1.383740 -0.561432

C 3.603588 -0.341382 -1.385910

C 4.288684 0.743919 -0.848283

C 4.556304 0.791304 0.519498

C 4.130548 -0.246833 1.345903

C 3.428818 -1.325732 0.809538

C 1.078384 1.229142 2.290428

C 1.213248 1.835967 0.907383

C 1.805195 3.096374 0.774744

C 1.963020 3.675559 -0.481489

C 1.519954 3.004310 -1.621851

C 0.928346 1.750159 -1.497449

C 0.782222 1.167148 -0.239888

C -3.492300 -0.024269 -0.213275

C -3.127798 0.535927 -1.452639

C -3.467576 1.849953 -1.767076

C -4.165013 2.652341 -0.868366

C -4.525084 2.109367 0.364233

C -4.198578 0.797324 0.690247

H 0.071208 -3.176870 0.372898

H -3.178604 -2.109422 -1.815835

H -2.815423 -3.265127 -0.537834

H -3.755527 -1.371773 2.145532

H -2.838425 -2.767888 1.607288

H -1.698167 1.155562 1.746770

H 2.819863 -2.817824 -2.136681

H 2.487701 -3.431118 -0.501927

H 3.379173 -0.373962 -2.454751

H 4.607407 1.561482 -1.497560

H 5.097154 1.641453 0.940787

H 4.342721 -0.219514 2.417411

H 3.072042 -2.120260 1.469104

H 2.028694 0.780575 2.611093

H 0.803479 2.002674 3.023348

H 2.143510 3.633341 1.665672

H 2.424380 4.661762 -0.569514

H 1.633365 3.462612 -2.606718

H 0.581710 1.202067 -2.375635

H 0.321185 0.180632 -0.165416

H -2.536231 -0.034149 -2.168788

H -3.158343 2.252964 -2.734350

H -4.418891 3.683720 -1.119321

H -5.076501 2.713224 1.089199

H -4.506485 0.411820 1.662389

**L3’**

90

N 0.026991 -3.169295 1.781740

N -1.051072 -3.461674 1.129680

N -0.701632 -4.051765 -0.009532

N 6.258540 -0.361509 0.585296

N 6.443962 0.669316 -0.173255

N 5.473575 0.681635 -1.087894

N 3.127369 -2.158705 0.888385

C 0.640068 -4.145780 -0.100610

C 1.105631 -3.570912 1.064785

C 2.516660 -3.335174 1.504307

C 4.584682 -2.202941 0.899206

C 5.152738 -1.029556 0.170286

C 4.635309 -0.361784 -0.916763

C -1.715643 -4.423930 -0.985312

C -2.058831 -3.306503 -1.943267

C -2.873401 -2.249698 -1.524430

C -3.201038 -1.216360 -2.396885

C -2.703647 -1.227349 -3.700623

C -1.884697 -2.272022 -4.126404

C -1.564591 -3.309398 -3.249619

C 5.260474 1.844544 -1.930600

C 4.057608 2.628119 -1.453571

C 2.882306 2.673615 -2.204614

C 1.761630 3.349274 -1.721408

C 1.809096 3.979035 -0.481033

C 2.978788 3.929175 0.277955

C 4.096535 3.256313 -0.203905

C 2.539901 -0.920187 1.308079

C 2.980509 -0.256463 2.431580

C 2.413210 0.979657 2.806217

C 1.358022 1.500230 2.099616

C 0.831130 0.820590 0.970387

C 1.487840 -0.359630 0.516008

C 1.117370 -0.919097 -0.736600

C -0.312491 1.311721 0.245373

C -0.642650 0.728419 -0.961079

C 0.102522 -0.360440 -1.467066

N -1.059378 2.371646 0.808647

N -4.043863 1.144263 2.258153

N -4.647038 0.000296 2.255241

N -3.723016 -0.945244 2.098771

N -3.494039 2.087326 -1.459169

N -4.756807 1.816060 -1.428245

N -5.288999 2.473793 -0.399840

C -2.487443 -0.404281 1.997058

C -2.707446 0.955843 2.097195

C -1.720900 2.091335 2.085422

C -1.768562 3.291610 -0.073048

C -3.180215 2.918709 -0.432382

C -4.347649 3.186563 0.248777

C -4.097418 -2.342529 2.125864

C -6.649178 2.212262 0.011890

H 1.142683 -4.584530 -0.957426

H 2.527283 -3.274379 2.610693

H 3.124724 -4.207811 1.220146

H 5.030607 -2.221024 1.914569

H 4.890826 -3.144812 0.412991

H 3.736304 -0.500728 -1.509516

H -2.598867 -4.730064 -0.408987

H -1.344538 -5.304398 -1.526982

H -3.246605 -2.230291 -0.499035

H -3.833586 -0.392800 -2.061964

H -2.955550 -0.411282 -4.380863

H -1.494723 -2.283689 -5.146595

H -0.929148 -4.132100 -3.590129

H 5.130043 1.514514 -2.971036

H 6.183951 2.435202 -1.867580

H 2.835712 2.170524 -3.174303

H 0.845462 3.368297 -2.315340

H 0.927896 4.492918 -0.092387

H 3.016112 4.410436 1.257388

H 5.006718 3.196509 0.398946

H 3.799997 -0.671098 3.022003

H 2.822710 1.522185 3.660841

H 0.940224 2.470565 2.368270

H 1.665555 -1.788498 -1.097121

H -0.181063 -0.788638 -2.431027

H -1.513949 1.075946 -1.517491

H -1.588805 -1.006790 1.880669

H -2.249817 3.000649 2.406197

H -0.959393 1.889661 2.851486

H -1.789062 4.277813 0.421560

H -1.177538 3.405442 -0.991557

H -4.562202 3.753433 1.148367

H -3.215631 -2.940109 1.861224

H -4.908219 -2.515727 1.405955

H -7.125468 3.143982 0.341882

H -6.646275 1.482326 0.834701

H -4.445821 -2.617719 3.130724

H -7.190072 1.805194 -0.849805

[Cu(L1)Br]_2_

120

Cu 0.872391 0.755170 -1.498216

N -0.935031 1.453508 -2.203353

N -1.979183 0.806399 -1.802626

N -3.004643 1.642598 -1.852554

N 2.661215 1.784327 -1.752672

N 3.650348 1.073886 -1.305812

N 4.547603 1.904102 -0.805573

N 0.626367 4.127337 -1.813936

C -2.617400 2.860778 -2.295625

C -1.268219 2.733268 -2.518701

C -0.233797 3.741193 -2.915558

C 2.046484 4.223341 -2.058267

C 2.915034 3.104931 -1.550658

C 4.135852 3.188793 -0.921662

C -4.248326 1.303766 -1.189127

C -4.424618 2.123830 0.070176

C -3.319429 2.415549 0.879447

C -3.490658 3.127523 2.063436

C -4.762867 3.548168 2.454967

C -5.865057 3.265745 1.647837

C -5.693298 2.562748 0.454560

C 5.698765 1.383142 -0.096750

C 5.413650 1.081828 1.360139

C 6.491462 0.826561 2.217432

C 6.268259 0.510344 3.554581

C 4.962399 0.452213 4.051185

C 3.890360 0.701524 3.199256

C 4.112544 1.010008 1.856398

C 0.093257 4.715931 -0.664007

C 0.835086 4.739197 0.530268

C 0.314701 5.322605 1.678893

C -0.958715 5.891758 1.680390

C -1.699595 5.878026 0.502085

C -1.180478 5.310103 -0.658906

Br 0.465203 1.274837 1.022958

Cu 0.022350 -1.071057 0.236646

N -1.805798 -1.528018 1.144806

N -2.782000 -0.685889 1.234974

N -3.671627 -1.191926 2.075970

N 1.437477 -2.437073 1.172494

N 2.569902 -1.936354 1.545575

N 3.486588 -2.880633 1.413415

N -0.827175 -4.143519 0.558270

C -3.274387 -2.404534 2.529983

C -2.059293 -2.614813 1.926848

C -1.157676 -3.811933 1.922805

C 0.505896 -4.588539 0.230375

C 1.603543 -3.731193 0.791106

C 2.937064 -4.029494 0.949451

C -4.965037 -0.563425 2.225251

C -5.933194 -0.942332 1.121425

C -5.586774 -1.825798 0.095979

C -6.501914 -2.122845 -0.915088

C -7.768752 -1.545242 -0.910263

C -8.122437 -0.668155 0.116803

C -7.209792 -0.369690 1.124350

C 4.887665 -2.548056 1.556004

C 5.549346 -2.244123 0.226785

C 6.947259 -2.204605 0.158075

C 7.587162 -1.902363 -1.041049

C 6.833504 -1.643984 -2.189560

C 5.443136 -1.676742 -2.121898

C 4.800052 -1.968378 -0.918013

C -1.882749 -4.373949 -0.344744

C -1.769968 -3.980385 -1.688375

C -2.825707 -4.175911 -2.570944

C -4.024701 -4.748556 -2.140148

C -4.152461 -5.122288 -0.805178

C -3.092423 -4.943474 0.083665

Br 1.086593 -1.655902 -2.048696

H -3.304277 3.698138 -2.366621

H 0.401574 3.282036 -3.684827

H -0.734099 4.608877 -3.388261

H 2.202778 4.298191 -3.147073

H 2.432445 5.164926 -1.633618

H 4.719098 4.029362 -0.557548

H -4.172389 0.234418 -0.947243

H -5.090969 1.444370 -1.881311

H -2.315805 2.074033 0.608955

H -2.613769 3.349469 2.674768

H -4.894754 4.105455 3.385368

H -6.862245 3.602111 1.941611

H -6.557975 2.340362 -0.176465

H 6.510243 2.118955 -0.190555

H 6.013134 0.471678 -0.623920

H 7.514262 0.870415 1.831914

H 7.116189 0.313947 4.214952

H 4.786926 0.207347 5.101225

H 2.858581 0.639927 3.550275

H 3.239925 1.161322 1.215765

H 1.801094 4.237112 0.577809

H 0.907816 5.303356 2.595927

H -1.369761 6.334357 2.589851

H -3.878304 -3.008822 3.199925

H -0.230304 -3.562752 2.454287

H -1.634332 -4.635757 2.490349

H 0.687767 -5.637100 0.547359

H 0.607534 -4.568803 -0.864231

H 3.523637 -4.920770 0.748517

H -4.805641 0.524176 2.239643

H -5.364911 -0.853136 3.207964

H -4.597700 -2.289410 0.060449

H -6.206080 -2.812829 -1.707905

H -8.482507 -1.777792 -1.703473

H -9.114850 -0.211929 0.131139

H -7.484266 0.330187 1.918314

H 5.395337 -3.383062 2.060801

H 4.934538 -1.674013 2.221229

H 7.540097 -2.409609 1.054196

H 8.678525 -1.876385 -1.082979

H 7.334224 -1.413955 -3.132759

H 4.831515 -1.455726 -2.998594

H 3.706953 -1.946839 -0.897938

H -0.867662 -3.463134 -2.023377

H -2.714558 -3.848601 -3.607149

H -4.851363 -4.896928 -2.838790

H -3.213754 -5.257758 1.121471

H -1.774299 5.350682 -1.570651

H -2.698603 6.319649 0.477211

H -5.084235 -5.565327 -0.445066

[Cu(L1)Br]

60

Cu -0.247164 -1.050014 0.223974

N 1.076364 -0.593214 1.953544

N 2.140111 -1.307415 2.126744

N 3.157561 -0.476105 2.302404

N -2.019547 -0.177279 0.610419

N -3.070914 -0.798274 1.044572

N -4.032733 0.100004 1.174160

N 0.067086 1.750351 0.289385

C 2.748696 0.814051 2.241615

C 1.394657 0.728312 2.028742

C 0.366986 1.766787 1.719001

C -1.278990 2.124809 -0.080100

C -2.289979 1.147848 0.448963

C -3.602589 1.333316 0.817184

C 4.516259 -0.989328 2.237313

C 5.056497 -0.917760 0.823116

C 4.306896 -1.449147 -0.232387

C 4.787125 -1.377153 -1.536700

C 6.022319 -0.781586 -1.798693

C 6.771672 -0.249774 -0.750484

C 6.287219 -0.314238 0.556752

C -5.371089 -0.316260 1.560713

C -6.335152 -0.289733 0.396058

C -7.551724 0.387599 0.493652

C -8.441890 0.397750 -0.580942

C -8.115205 -0.264394 -1.761786

C -6.897753 -0.939759 -1.866031

C -6.012360 -0.954802 -0.792550

C 1.121795 2.287649 -0.518098

C 2.084026 1.423546 -1.047455

C 3.160467 1.938791 -1.767317

C 3.270308 3.311590 -1.984912

C 2.299830 4.175089 -1.476610

C 1.234143 3.665962 -0.736608

Br 0.857667 -2.240703 -1.429533

H 3.432561 1.654491 2.309475

H -0.552154 1.519931 2.270415

H 0.706799 2.762360 2.064462

H -1.576701 3.143950 0.246632

H -1.327179 2.119388 -1.180275

H -4.244754 2.208647 0.839723

H 4.464332 -2.027206 2.593924

H 5.142290 -0.415192 2.934096

H 3.326944 -1.906394 -0.058713

H 4.172492 -1.784362 -2.342693

H 6.399301 -0.727341 -2.822641

H 7.736013 0.223276 -0.948889

H 6.875482 0.111678 1.374836

H -5.719053 0.334374 2.375210

H -5.255920 -1.332270 1.962655

H -7.810429 0.912065 1.417773

H -9.391763 0.929426 -0.494218

H -8.809024 -0.254551 -2.604976

H -6.636966 -1.460457 -2.789633

H -5.059186 -1.484387 -0.876238

H 1.969807 0.345293 -0.908533

H 3.913284 1.252774 -2.161108

H 4.111607 3.710230 -2.556196

H 2.376693 5.251066 -1.649364

H 0.486455 4.346723 -0.320532

[Cu(L1)_2_]^+^

117

Cu 0.232812 -0.623058 0.224828

N 0.167680 -0.536144 3.517409

N -1.217486 1.411995 1.863261

N -2.022578 1.871958 0.963925

N -3.220575 1.340898 1.178151

N 1.732679 0.368978 1.187860

N 2.666577 0.785454 0.382896

N 3.555501 1.434886 1.098811

C 2.090984 -3.570208 2.602914

C 1.132423 -4.323210 1.927324

C -0.153589 -3.806517 1.799390

C -0.483941 -2.559445 2.325952

C 0.481021 -1.791380 3.003420

C 1.778728 -2.321771 3.130952

C -1.197672 -0.157293 3.802914

C -1.889496 0.557831 2.675988

C -3.197498 0.515795 2.249251

C -4.330588 1.688920 0.309560

C 1.185944 0.466110 3.681147

C 2.024383 0.740415 2.460382

C 3.218665 1.428291 2.407351

C 4.775898 1.948396 0.478901

N 0.015915 0.677760 -2.305515

N 1.613324 -1.409568 -1.308321

N 2.511175 -2.268658 -0.936980

N 3.608056 -2.014990 -1.627653

N -1.588938 -1.031143 -0.549832

N -2.619486 -1.530742 0.058196

N -3.598911 -1.612107 -0.821798

C -2.247047 3.644095 -2.112197

C -1.499318 4.499605 -1.311415

C -0.258936 4.069697 -0.837790

C 0.225071 2.811428 -1.163486

C -0.510899 1.951726 -1.992198

C -1.760729 2.381154 -2.456227

C 1.379189 0.601987 -2.796893

C 2.126951 -0.579340 -2.249812

C 3.425313 -0.974990 -2.473582

C 4.852383 -2.671232 -1.264844

C -0.871940 -0.322511 -2.848482

C -1.891228 -0.793087 -1.855327

C -3.201920 -1.167183 -2.036842

C -4.869455 -2.220626 -0.451936

H 1.386019 -5.297509 1.507538

H -0.921696 -4.374747 1.269754

H -1.492840 -2.174389 2.177292

H 2.549992 -1.760910 3.661158

H -1.196133 0.487785 4.697803

H -1.771757 -1.051263 4.084672

H -4.071020 -0.029590 2.592855

H -3.886455 1.865567 -0.680868

H 1.873344 0.231970 4.517510

H 0.684766 1.406284 3.949813

H 3.826449 1.898375 3.174829

H 5.155501 2.737340 1.143720

H -1.882346 5.487901 -1.050652

H 0.329259 4.716240 -0.182304

H 1.171029 2.463453 -0.749462

H -2.367080 1.744878 -3.101706

H 1.408312 0.583791 -3.904661

H 1.919984 1.508151 -2.493631

H 4.208070 -0.594997 -3.121174

H 4.589788 -3.693533 -0.960441

H 5.482115 -2.733568 -2.161905

H -1.381515 -0.003768 -3.778172

H -0.260935 -1.196033 -3.119497

H -3.852816 -1.171258 -2.906185

H -5.680485 -1.538382 -0.744063

H -3.222430 3.962228 -2.488274

H 3.103662 -3.961102 2.727358

H -4.990113 0.812695 0.239669

H -4.854399 -2.285136 0.644990

H 5.516836 1.136237 0.462467

C -4.238099 -5.818708 -1.504667

C -5.395377 -6.107841 -2.230265

C -6.379910 -5.135055 -2.383750

C -4.069212 -4.561740 -0.932477

C -5.055561 -3.580486 -1.084272

C -6.208352 -3.872549 -1.814736

H -3.465170 -6.579932 -1.381771

H -5.527992 -7.095252 -2.676761

H -7.285913 -5.355953 -2.951336

H -3.162211 -4.336463 -0.364544

H -6.983735 -3.111785 -1.940266

C 5.580573 -1.115285 2.131353

C 6.899397 -0.682614 1.973465

C 7.545790 -0.868657 0.753077

C 4.912681 -1.734096 1.078325

C 5.565166 -1.942136 -0.143574

C 6.880113 -1.498569 -0.300831

H 5.068399 -0.976578 3.086184

H 7.424822 -0.205160 2.803224

H 8.576374 -0.533282 0.620976

H 3.876901 -2.061451 1.200818

H 7.400137 -1.657813 -1.249573

C 5.183481 1.898364 -2.001877

C 4.533156 2.477135 -0.911469

C 3.662340 3.550023 -1.126261

C 4.958356 2.376006 -3.293351

C 4.072558 3.431558 -3.502090

C 3.424916 4.020062 -2.414569

H 5.871296 1.064907 -1.833507

H 3.151981 4.014558 -0.278094

H 5.479208 1.923922 -4.140505

H 3.891621 3.803688 -4.512427

H 2.728939 4.846299 -2.571032

C -6.486434 2.841453 0.949182

C -5.102399 2.900948 0.777869

C -4.430501 4.103878 1.024256

C -7.197352 3.970451 1.357415

C -6.524339 5.165006 1.602139

C -5.139651 5.228448 1.436371

H -7.020014 1.904957 0.762549

H -3.346446 4.147781 0.890984

H -8.279822 3.913504 1.488573

H -7.078470 6.048935 1.924378

H -4.609319 6.163507 1.628762

[Cu(L3')Br]_2_

184

Cu 0.741279 2.224965 1.142153

N 1.011536 0.469000 2.199497

N 0.539220 -0.627311 1.702347

N 1.159543 -1.632048 2.304071

N 1.783174 3.834451 2.253965

N 1.173909 4.917054 1.875420

N 2.060369 5.894638 1.861553

N 3.818464 1.750959 3.098374

C 2.044302 -1.181410 3.221469

C 1.932087 0.187673 3.160069

C 2.691795 1.253933 3.878767

C 4.092733 3.159325 3.127735

C 3.092071 4.108657 2.505023

C 3.281139 5.446844 2.235388

C 0.980818 -2.979835 1.813937

C 1.665087 -3.231797 0.484509

C 2.162824 -2.201602 -0.317997

C 2.725059 -2.492128 -1.562241

C 2.786043 -3.804698 -2.021630

C 2.296199 -4.838584 -1.220942

C 1.744779 -4.552472 0.025469

C 1.687853 7.206808 1.373942

C 1.825286 7.349073 -0.126839

C 1.857045 8.632809 -0.685131

C 1.935597 8.799079 -2.065608

C 1.990516 7.680783 -2.902411

C 1.956120 6.403637 -2.348392

C 1.867871 6.235853 -0.966092

C 4.438828 0.918884 2.141485

C 4.412498 1.234826 0.801814

C 4.971195 0.361832 -0.155303

C 5.509870 -0.845967 0.216928

C 5.560026 -1.209185 1.590808

C 5.083501 -0.288754 2.570824

Br 1.781289 2.521471 -1.130661

C 5.285787 -0.571539 3.949989

C 6.119846 -2.460924 2.030045

C 6.247631 -2.714928 3.381835

C 5.861816 -1.752254 4.342557

N 6.550985 -3.389305 1.054164

N 8.882201 -5.797898 -0.185061

N 9.474756 -5.766587 -1.336810

N 9.508695 -4.495198 -1.742237

N 6.073246 -3.489924 -2.052371

N 6.471811 -4.077925 -3.132375

N 6.505597 -5.390926 -2.894107

C 8.929754 -3.685244 -0.832553

C 8.519798 -4.539990 0.167082

C 7.696411 -4.224153 1.380207

C 5.479859 -4.058745 0.318435

C 5.850482 -4.418095 -1.086842

C 6.119757 -5.654065 -1.627166

C 10.001994 -4.151114 -3.055397

C 7.042224 -6.306948 -3.873074

H 2.687504 -1.836100 3.802297

H 1.998445 2.087529 4.059292

H 3.000314 0.863919 4.867159

H 4.215257 3.476535 4.182191

H 5.070819 3.326794 2.653646

H 4.153038 6.092281 2.283486

H -0.098826 -3.162527 1.722919

H 1.368602 -3.663056 2.582213

H 2.096829 -1.158822 -0.005704

H 3.117369 -1.675046 -2.169280

H 3.239777 -4.023511 -2.990136

H 2.353141 -5.873679 -1.565714

H 1.356740 -5.363942 0.647835

H 2.312226 7.947316 1.893699

H 0.646847 7.374601 1.683112

H 1.815534 9.510227 -0.033086

H 1.961027 9.805102 -2.490917

H 2.056377 7.810376 -3.985104

H 1.975363 5.511433 -2.977277

H 1.804060 5.217654 -0.574177

H 3.895145 2.134907 0.467347

H 4.921611 0.641226 -1.209782

H 5.897362 -1.528616 -0.540997

H 5.005180 0.180235 4.687476

H 6.025117 -1.958628 5.402946

H 6.660431 -3.667239 3.719533

H 8.792741 -2.619072 -0.983508

H 7.413026 -5.187284 1.851766

H 8.312677 -3.677427 2.108201

H 5.164095 -4.982170 0.845834

H 4.605986 -3.394438 0.296199

H 6.126712 -6.653177 -1.202970

H 10.501456 -5.036538 -3.464947

H 10.720748 -3.324116 -2.987786

H 8.024701 -6.671675 -3.537181

H 7.145093 -5.760727 -4.817686

H 9.160657 -3.856807 -3.700808

H 6.361658 -7.156855 -4.014164

Cu -0.718118 2.232762 -1.124686

N -1.020190 0.485281 -2.187757

N -0.561456 -0.620081 -1.698161

N -1.192207 -1.613094 -2.308380

N -1.729119 3.857690 -2.244246

N -1.094209 4.929838 -1.878390

N -1.959618 5.926061 -1.865550

N -3.803178 1.812034 -3.071966

C -2.070282 -1.145380 -3.223747

C -1.942242 0.221747 -3.151963

C -2.690085 1.301715 -3.862634

C -4.059323 3.224029 -3.093642

C -3.033727 4.157605 -2.487753

C -3.192340 5.501674 -2.227343

C -1.028615 -2.966409 -1.828413

C -1.714319 -3.220296 -0.500085

C -2.204561 -2.190810 0.307927

C -2.768302 -2.483712 1.550923

C -2.838415 -3.798166 2.003569

C -2.356528 -4.831327 1.197216

C -1.803472 -4.542695 -0.047905

C -1.555927 7.233226 -1.389543

C -1.689181 7.391167 0.110057

C -1.693183 8.679777 0.657876

C -1.767478 8.858912 2.037002

C -1.846020 7.748909 2.882896

C -1.839169 6.466842 2.339266

C -1.755035 6.285962 0.958374

C -4.433578 0.982031 -2.119747

C -4.397198 1.286907 -0.777704

C -4.966007 0.415314 0.174662

C -5.524781 -0.781118 -0.204851

C -5.585610 -1.132770 -1.581277

C -5.098507 -0.212096 -2.555966

Br -1.749746 2.573218 1.144559

C -5.310826 -0.480793 -3.936476

C -6.166684 -2.372145 -2.028093

C -6.303922 -2.613288 -3.381288

C -5.906935 -1.649127 -4.335940

N -6.608382 -3.301580 -1.057919

N -8.965902 -5.688269 0.170724

N -9.553114 -5.659306 1.325249

N -9.569945 -4.390974 1.741224

N -6.120143 -3.432626 2.045340

N -6.521227 -4.022525 3.123387

N -6.573853 -5.333319 2.876455

C -8.984975 -3.580627 0.835771

C -8.589650 -4.431997 -0.172628

C -7.767482 -4.116329 -1.386563

C -5.544923 -3.993466 -0.331942

C -5.914633 -4.357139 1.072492

C -6.197932 -5.593008 1.605835

C -10.052458 -4.052155 3.059751

C -7.118771 -6.248425 3.851681

H -2.720188 -1.788116 -3.810420

H -1.986000 2.125700 -4.045294

H -3.010741 0.919420 -4.850180

H -4.197823 3.546466 -4.144673

H -5.025800 3.402814 -2.600390

H -4.050592 6.165251 -2.273966

H 0.048960 -3.162230 -1.739792

H -1.425018 -3.639429 -2.601296

H -2.131684 -1.147113 0.000420

H -3.155212 -1.667367 2.162373

H -3.293052 -4.018618 2.971281

H -2.420610 -5.867770 1.536632

H -1.421437 -5.353701 -0.674565

H -2.162591 7.984133 -1.915342

H -0.511362 7.373015 -1.700575

H -1.633285 9.550769 -0.001315

H -1.771150 9.868677 2.454103

H -1.908734 7.888705 3.964507

H -1.877310 5.580446 2.975462

H -1.713117 5.263432 0.574806

H -3.864770 2.176440 -0.438527

H -4.908744 0.685948 1.231063

H -5.920337 -1.463391 0.549247

H -5.021864 0.272415 -4.669242

H -6.077834 -1.844402 -5.397232

H -6.732910 -3.556289 -3.724859

H -8.834461 -2.517467 0.994889

H -7.500473 -5.078887 -1.868743

H -8.378988 -3.553461 -2.106222

H -5.246374 -4.917953 -0.867436

H -4.660500 -3.343335 -0.309053

H -6.220399 -6.589027 1.174958

H -10.566379 -4.932722 3.461821

H -10.756350 -3.211677 3.003356

H -8.109331 -6.594415 3.519673

H -7.206219 -5.708471 4.801441

H -9.203772 -3.779707 3.705178

H -6.451293 -7.110480 3.980989

[Cu(L3')Br]

92

Cu 3.306816 -1.253061 0.962981

N 2.301919 -3.031495 0.870291

N 1.514814 -3.251371 1.878395

N 0.351817 -3.647169 1.390106

N 4.656756 -1.011717 -0.642803

N 5.683770 -0.366421 -0.173102

N 6.077517 0.485026 -1.098621

N 2.161984 -1.761873 -2.160215

C 0.375645 -3.696265 0.038136

C 1.653262 -3.308137 -0.295645

C 2.307978 -3.113794 -1.627326

C 3.303517 -1.161968 -2.793883

C 4.379191 -0.581085 -1.904054

C 5.295060 0.403658 -2.200419

C -0.786153 -3.744614 2.281885

C -1.438555 -2.402377 2.559780

C -0.770934 -1.193265 2.348669

C -1.381713 0.012644 2.693104

C -2.654532 0.022893 3.256006

C -3.328427 -1.181697 3.461202

C -2.725798 -2.386805 3.109688

C 7.066123 1.498763 -0.767899

C 6.408334 2.814436 -0.406968

C 6.915499 4.018477 -0.900561

C 6.315426 5.229055 -0.552889

C 5.199344 5.239567 0.282206

C 4.687553 4.037321 0.771884

C 5.290070 2.828903 0.433866

C 1.045337 -0.967908 -1.810971

C 1.192244 0.218007 -1.123625

C 0.063506 0.960879 -0.718621

C -1.207603 0.498546 -0.952501

C -1.404335 -0.719935 -1.658705

C -0.269583 -1.422808 -2.160418

Br 2.717172 0.652599 2.179777

C -0.463963 -2.538946 -3.020760

H -0.488756 -3.960553 -0.564565

H 3.380011 -3.314654 -1.486377

H 1.922720 -3.881406 -2.323525

H 3.779247 -1.916621 -3.446508

H 2.953193 -0.364785 -3.467037

H 5.434973 1.042570 -3.067186

H -0.413199 -4.187262 3.217063

H -1.504752 -4.447989 1.838502

H 0.240207 -1.157271 1.937432

H -0.837275 0.942323 2.519229

H -3.130131 0.970986 3.513882

H -4.333070 -1.182034 3.890170

H -3.259173 -3.327946 3.273920

H 7.749138 1.614744 -1.620886

H 7.637689 1.090420 0.077279

H 7.785979 4.014610 -1.563173

H 6.718753 6.166209 -0.942967

H 4.725674 6.186909 0.549312

H 3.811774 4.021086 1.424222

H 4.864418 1.902091 0.833427

H 2.182854 0.570793 -0.833281

H 0.218565 1.888332 -0.164575

H -2.066628 1.066831 -0.592796

C -2.719358 -1.244590 -1.924737

C -2.856508 -2.366401 -2.720564

C -1.728371 -2.990602 -3.299255

N -3.837300 -0.575840 -1.381875

H 0.404474 -3.007091 -3.483438

H -1.872604 -3.840968 -3.969857

H -3.848432 -2.776989 -2.915998

N -7.192918 0.624423 -1.576865

N -7.586254 1.843200 -1.381997

N -6.552132 2.649284 -1.632893

N -4.009816 1.735670 0.734440

N -4.815591 2.603121 1.253487

N -5.945359 1.969553 1.575116

C -5.467556 1.938438 -2.002801

C -5.890793 0.628322 -1.953732

C -5.080485 -0.619995 -2.136519

C -3.984503 -0.682940 0.069569

C -4.612924 0.520070 0.701205

C -5.865170 0.660861 1.252521

C -6.645312 4.069689 -1.387214

C -7.090785 2.700804 2.063999

H -4.497886 2.386645 -2.195412

H -5.724895 -1.479337 -1.860811

H -4.823511 -0.735733 -3.199136

H -4.589853 -1.574089 0.331905

H -2.991259 -0.838139 0.511807

H -6.692670 -0.030444 1.377682

H -7.698933 4.304935 -1.198267

H -6.296502 4.632153 -2.263102

H -7.868762 2.731226 1.286362

H -6.757668 3.716761 2.304902

H -6.034060 4.333879 -0.511114

H -7.491179 2.223662 2.968097

[Cu(L3')_2_]^+^

181

Cu -1.249314 1.020687 -0.071462

N -0.108499 2.548364 -0.937667

N -0.544893 3.751469 -0.719010

N 0.056350 4.561653 -1.562204

N -0.666854 -0.690693 -1.286265

N -1.437638 -1.715215 -1.483805

N -0.705091 -2.802537 -1.297017

N 2.323630 0.523922 -1.645397

C 0.935846 3.883514 -2.333838

C 0.830979 2.570388 -1.925840

C 1.511819 1.352499 -2.504700

C 1.660741 -0.174101 -0.555051

C 0.587071 -1.112564 -0.981098

C 0.570647 -2.486304 -0.989491

C -0.222725 5.998490 -1.590439

C -1.480545 6.392420 -0.855450

C -1.440072 7.463084 0.038920

C -2.593052 7.874461 0.709682

C -3.795881 7.203950 0.502722

C -3.846731 6.139212 -0.399959

C -2.700883 5.745863 -1.083608

C -1.337718 -4.107554 -1.254212

C -2.077529 -4.359575 0.045694

C -2.023486 -5.628694 0.630759

C -2.736208 -5.906552 1.797194

C -3.519665 -4.915639 2.390687

C -3.584109 -3.648732 1.809853

C -2.869529 -3.373065 0.645585

C 3.663970 0.860085 -1.351074

C 4.081787 2.140063 -1.063784

C 5.417123 2.390691 -0.665184

C 6.294833 1.352014 -0.478793

C 5.901012 0.012078 -0.755811

C 4.602886 -0.226618 -1.286731

N -1.391640 -0.131589 1.659190

N -0.693357 -1.224549 1.655327

N -0.872073 -1.826006 2.808867

N -0.721406 2.870397 2.220630

N 0.425926 3.462495 2.288883

N 0.199894 4.763944 2.436734

N -3.447158 1.989510 2.208261

C -1.740726 -1.134281 3.580849

C -2.068626 -0.022978 2.831782

C -2.926609 1.133446 3.261221

C -3.174808 3.401692 2.365226

C -1.715566 3.784757 2.326591

C -1.127712 5.022766 2.466574

C -0.254734 -3.120287 3.081192

C 1.083043 -3.313316 2.404267

C 1.389455 -4.565117 1.865677

C 2.634246 -4.802558 1.278315

C 3.577113 -3.780156 1.199032

C 3.270129 -2.521531 1.720410

C 2.040794 -2.293599 2.334904

C 1.309163 5.681961 2.636041

C 1.974665 6.173882 1.370652

C 2.067116 7.545231 1.115500

C 2.741265 8.017148 -0.011091

C 3.338615 7.115427 -0.889789

C 3.254388 5.744880 -0.637834

C 2.573308 5.272611 0.482737

C -3.767458 1.487118 0.940662

C -4.657323 0.363861 0.808522

C -4.877837 -0.202107 -0.483432

C -4.303556 0.418426 -1.625457

C -3.559781 1.562887 -1.489134

C -3.271582 2.097051 -0.208656

C 4.270228 -1.520237 -1.767437

C -5.431080 -0.105901 1.905515

H 1.546065 4.363925 -3.092970

H 0.720209 0.718514 -2.936736

H 2.139236 1.686826 -3.343019

H 2.416345 -0.758327 -0.020991

H 1.236497 0.531703 0.187352

H 1.329758 -3.226336 -0.750203

H -0.292656 6.281207 -2.652028

H 0.644135 6.521069 -1.163802

H -0.493898 7.979989 0.214684

H -2.548262 8.721262 1.398068

H -4.698299 7.518283 1.031130

H -4.788787 5.616797 -0.580147

H -2.756847 4.916386 -1.791613

H -0.552211 -4.859219 -1.402248

H -2.030290 -4.186240 -2.106359

H -1.423302 -6.413933 0.163677

H -2.679184 -6.902354 2.242625

H -4.080185 -5.132751 3.302862

H -4.203898 -2.863786 2.249122

H -2.936259 -2.379017 0.199596

H 3.361733 2.956870 -1.110061

H 5.734841 3.417880 -0.470064

H 7.320086 1.534803 -0.153405

H -2.043653 -1.465224 4.569701

H -2.300118 1.764285 3.908244

H -3.721319 0.732341 3.914571

H -3.596763 3.734160 3.330277

H -3.728193 3.957279 1.595218

H -1.532959 6.024291 2.572127

H -0.149857 -3.189036 4.174617

H -0.954653 -3.907294 2.764355

H 0.644529 -5.364337 1.904289

H 2.865014 -5.791332 0.876483

H 4.553134 -3.952581 0.742648

H 4.005412 -1.716768 1.641537

H 1.813739 -1.301746 2.732859

H 0.931310 6.529423 3.222487

H 2.040416 5.148384 3.259340

H 1.616576 8.257730 1.812074

H 2.808019 9.091157 -0.195707

H 3.879723 7.479969 -1.765331

H 3.735480 5.039995 -1.319528

H 2.504043 4.198631 0.679793

H -4.513602 0.008959 -2.612314

H -2.696715 3.021605 -0.131251

H -3.164557 2.068061 -2.373358

C 6.790391 -1.099451 -0.568995

C 6.432628 -2.341129 -1.056839

C 5.186680 -2.538365 -1.688062

C -6.357988 -1.101619 1.728588

C -6.517828 -1.723681 0.473001

C -5.778707 -1.317668 -0.620161

H 3.296247 -1.671691 -2.234076

H 4.948408 -3.523675 -2.094661

H 7.116483 -3.183188 -0.954196

H -5.352320 0.389261 2.870912

H -6.989529 -1.415028 2.562401

H -7.273634 -2.502726 0.363182

N 9.467410 -2.799905 -2.484489

N 9.688263 -2.479554 -3.715151

N 9.777020 -1.151426 -3.783842

N 7.394262 -3.102665 1.993761

N 6.676591 -3.842390 2.771567

N 5.995799 -3.042179 3.587942

N 8.010932 -0.875596 0.113388

C 9.611566 -0.595633 -2.562108

C 9.416727 -1.674736 -1.726371

C 9.146415 -1.720136 -0.258034

C 7.865897 -0.670829 1.550023

C 7.182634 -1.792735 2.287672

C 6.270496 -1.743359 3.321305

C 10.020266 -0.503517 -5.052112

C 5.081506 -3.599544 4.558266

H 9.633596 0.476186 -2.389805

H 9.023214 -2.774223 0.043313

H 10.019045 -1.336161 0.294147

H 8.874372 -0.515812 1.965206

H 7.305479 0.257239 1.742977

H 5.815701 -0.920841 3.865875

H 10.083768 -1.291280 -5.811010

H 10.965578 0.055500 -5.025948

H 5.322400 -3.232726 5.564921

H 5.200634 -4.688224 4.526621

H 9.195946 0.178976 -5.300156

H 4.044948 -3.338898 4.300343

N -9.387146 -1.588462 -1.207468

N -10.135817 -0.540175 -1.127938

N -9.587316 0.412608 -1.880510

N -4.022918 -5.001113 -2.672652

N -4.110757 -6.159545 -2.101798

N -4.957376 -6.046236 -1.084289

N -5.950450 -1.889720 -1.898577

C -8.451832 -0.030847 -2.466231

C -8.335329 -1.331891 -2.026506

C -7.275387 -2.352479 -2.283607

C -4.847387 -2.663641 -2.439192

C -4.812893 -4.109535 -2.021428

C -5.426739 -4.781884 -0.985671

C -10.219368 1.707980 -1.988996

C -5.300394 -7.199905 -0.284428

H -7.837101 0.587237 -3.113637

H -7.594068 -3.284873 -1.780689

H -7.227836 -2.589869 -3.358754

H -4.878229 -2.632669 -3.541387

H -3.896332 -2.189967 -2.146089

H -6.111834 -4.471329 -0.204207

H -11.098131 1.695566 -1.334777

H -10.536815 1.897967 -3.023315

H -5.278465 -6.932280 0.779540

H -4.552305 -7.975012 -0.484569

H -9.530260 2.499023 -1.663838

H -6.296870 -7.576885 -0.553748

[(CuBr)_2_(L3')]

94

Cu -2.697138 -0.765685 -0.767408

N -1.584425 -2.383488 -0.434790

N -0.588326 -2.670345 -1.217092

N 0.017832 -3.726677 -0.714085

N -4.671970 -1.413396 0.082143

N -5.600224 -1.071765 -0.757405

N -6.668029 -0.737934 -0.059408

N -2.949421 -2.015296 2.352507

C -0.589990 -4.139429 0.421935

C -1.640369 -3.266508 0.598406

C -2.708569 -3.279041 1.653793

C -4.331869 -1.680838 2.577930

C -5.141586 -1.312273 1.354179

C -6.441354 -0.862100 1.269696

C 1.104967 -4.380982 -1.452018

C 2.251736 -3.474435 -1.825898

C 2.090068 -2.494505 -2.812712

C 3.156136 -1.668929 -3.161259

C 4.394827 -1.817974 -2.539327

C 4.565250 -2.799144 -1.563836

C 3.498648 -3.623379 -1.209173

C -7.801191 -0.119516 -0.729999

C -7.751818 1.389846 -0.625116

C -8.882974 2.111843 -0.239114

C -8.834804 3.503829 -0.156011

C -7.651457 4.178412 -0.450274

C -6.518102 3.458498 -0.831422

C -6.565589 2.070449 -0.923011

C -1.997090 -0.984314 2.274853

C -2.308805 0.285622 1.827049

C -1.289951 1.234330 1.579016

C 0.029711 0.885755 1.697124

C 0.381451 -0.393940 2.202333

C -0.638433 -1.288843 2.621977

Br -2.870650 1.332295 -1.754799

C -0.276449 -2.437843 3.375447

C 1.750473 -0.800250 2.320360

C 2.066289 -1.933315 3.041931

C 1.045270 -2.723132 3.619607

N 2.703221 -0.068621 1.581506

N 4.643315 2.490725 1.071373

N 4.631532 3.770534 1.250873

N 4.100745 3.997290 2.444140

N 3.229449 1.420282 -1.431041

N 2.651146 2.172864 -2.314462

N 1.365885 1.853285 -2.310044

C 3.757952 2.836557 3.052158

C 4.114904 1.856827 2.153031

C 3.940539 0.365653 2.206511

C 2.740788 -0.430423 0.164259

C 2.320106 0.601152 -0.837236

C 1.098933 0.876972 -1.413673

C 3.971826 5.351082 2.935609

C 0.446923 2.506563 -3.221118

H -0.237760 -4.982660 1.007615

H -3.645493 -3.571274 1.153334

H -2.463239 -4.093799 2.358393

H -4.824893 -2.535713 3.073244

H -4.372551 -0.852131 3.300603

H -7.189227 -0.613729 2.016736

H 0.653476 -4.826186 -2.351291

H 1.454959 -5.201793 -0.813313

H 1.117429 -2.363979 -3.290889

H 3.015316 -0.889864 -3.913559

H 5.226372 -1.148445 -2.768200

H 5.530292 -2.880192 -1.059530

H 3.635411 -4.384804 -0.435719

H -8.726916 -0.519794 -0.294030

H -7.741907 -0.450416 -1.776071

H -9.812135 1.585859 -0.001225

H -9.724646 4.060813 0.145289

H -7.611718 5.267730 -0.380950

H -5.582306 3.971208 -1.063648

H -5.663583 1.526870 -1.224468

H -3.337103 0.557638 1.585213

H -1.573437 2.216528 1.198366

H 0.829737 1.574424 1.425857

H -1.064522 -3.067806 3.790416

H 1.315209 -3.590061 4.227177

H 3.111152 -2.238497 3.136285

H 3.302989 2.797205 4.037451

H 4.821591 -0.095330 1.723625

H 3.936462 0.054255 3.260535

H 3.769857 -0.726486 -0.099223

H 2.114633 -1.321152 0.013016

H 0.096787 0.477514 -1.267788

H 4.301086 6.020974 2.133796

H 4.604454 5.500580 3.821050

H -0.578721 2.241613 -2.930745

H 0.642263 2.174918 -4.249540

H 2.925081 5.565956 3.188421

H 0.588988 3.591977 -3.155636

Cu 5.121805 1.380373 -0.694800

Br 6.977443 -0.006402 -0.635547
